# Supplementary material for: Chemoenzymatic total synthesis of sorbicillactone A
Source: Commun Chem. 2024 Feb 24;7:39. doi: 10.1038/s42004-024-01126-1 (PMC10894215; doi:10.1038/s42004-024-01126-1)
Supplement: Supplementary file 2 — Supplementary Information [file 42004_2024_1126_MOESM2_ESM.pdf]

Chemoenzymatic Total Synthesis of Sorbicillactone A

Jonas I. Müller[1], Tobias A. M. Gulder[1,2]

1 Chair of Technical Biochemistry, Technical University of Dresden, Bergstraße 66, 01069 Dresden, Germany, E-mail: tobias.gulder@tu-dresden.de
2 Helmholtz Institute for Pharmaceutical Research Saarland (HIPS), Department of Natural Product Biotech-nology, Helmholtz Centre for Infection Research (HZI) and Department of Pharmacy at Saarland University, 66123 Saarbrücken, Germany.

Supplementary Information

Contents

1. General Information 1
1.1 Chemistry 1
1.2 Biochemistry/Molecular Biology 2
2. Experimental Procedures 3
3. Total Synthesis of Sorbicillactone A by Harned et al. 22
4. Supplementary References 22

Supplementary Methods

1. General Information

1.1 Chemistry

Reagents: Solvents for HPLC and MS analysis, such as acetonitrile and methanol, were purchased Scientific and VWR in a purity of over 99% (HPLC-grade). Water was purified using a TKA GenPure water treatment system and deionized. Dry solvents, such as diethyl ether, dichloromethane, 1,4-dioxane, tetrahydrofuran and toluene, for procedures under inert atmosphere were prepared by distillation and drying over molecular sieve (3 Å or 4 Å). Commercial materials and other solvents were purchased at the highest commercially available quality from the providers Acros Organics, Alfa Aesar, Carbolution, Carl Roth, Merck, Sigma Aldrich, VWR, TCI Chemicals, BLD Pharmatech and Thermo Fisher Scientific. Air- and moisture-sensitive reactions were performed under an argon atmosphere using a Schlenk line. Before application, the flasks were repeatedly evacuated (external heating) and refilled with argon.

NMR: ^{1}H and ^{13}C Nuclear Magnetic Resonance Spectra (NMR) were recorded on Bruker AV300 and AV600 spectrometers at 298 K. The chemical shifts are given in δ-values (ppm) and are calibrated on the residual peak of the deuterated solvent ($CDCl_3$: δ_H = 7.26 ppm, δ_C = 77.0 ppm; DMSO-d_6: δ_H = 2.50 ppm, δ_C = 39.5 ppm; MeOD-d_4: δ_H = 3.31 ppm, δ_C = 49.0 ppm; acetone-d_6: δ_H = 2.05 ppm, δ_C = 29.8 ppm). The coupling constants *J* are given in Hertz [Hz]. Following abbreviations were used for the allocation of signal multiplicities: bs – broad signal, s – singlet, d – doublet, dd – doublet of doublets, ddd – doublet of doublet of doublets, t – triplet, dt – doublet of triplets, ddt – doublet of doublet of triplets, q – quartet, pp = pseudopentet, sx – sextet, m – multiplet.

MS: Electrospray-Ionization Mass spectra (ESI-MS) were recorded on an Advion expression LCMS system using a single-quadrupole mass analyzer, a Peak Scientific N118LA nitrogen generator, an Edwards RV12 high vacuum pump and a Jasco PU-1580 Intelligent HPLC Pump. For high resolution mass spectrometry (HRMS), a LTQ FT Ultra with ESI and linear ion trap manufactured by Thermo Fisher Scientific or a Bruker Impact II ultra-high resolution Q-TOF mass spectrometer with ESI were used.

Chromatography: Thin-layer chromatography (TLC) was performed on precoated plates of silica gel F254 (Merck) with UV detection at 254 and 365 nm. For UV-inactive substances, the plates were treated with $KMnO_4$ stain and gently heated. Column chromatography was performed on silica gel 60 Geduran® Si 60 (40–60 µm) (Merck). High Performance Liquid Chromatography was performed on an Azura HPLC device manufactured by Knauer, consisting of the following components: AS 6.1L sampler, P 6.1L pump, DAD 2.1L detector. The system was controlled by ClarityChrom software. A Eurosphere II 100-3 C18 A (150 x 4.6 mm) column with integrated precolumn manufactured by Knauer was used with the following eluent solvents: A = H_2O + 0.05% TFA, B = ACN + 0.05% TFA. The separation method consisted of the following gradient system: 0–1 min 5% B, 1–15 min to 95% B, 15–18 min 95% B, 18–18.5 min to 5% B, 18.5–20 min 5% B with a flowrate of 1 mL/min. For medium pressure liquid chromatography (MPLC) the Reveleris® X2 MPLC system (Grace) was used together with Reveleris® Reverse Phase (RP) C18 columns (Grace) using UV-detection at 220 nm, 254 nm, and 370 nm. Isolation of the chemoenzymatically produced compounds was carried out by semi-preparative HPLC controlled by a Jasco HPLC system consisting of an UV-1575 Intelligent S3 UV/VIS Detector, two PU-2068

Intelligent prep. Pumps, a MIKA 1000 Dynamic Mixing Chamber (1000 µL Portmann Instruments AG Biel-Benken), a LC-NetII/ ADC, and a Rheodyne injection valve. The system was controlled by the Galaxie-Software and the eluent system consisted of: A = H_2O + 0.05% TFA and B = ACN + 0.05% TFA. A Eurosphere II 100-5 C18 A (250 x 16 mm) column with precolumn (30 x 16 mm) provided by Knauer was used as the stationary phase. General HPLC condition: gradient: 0–1 min 95% H_2O + 0.05% TFA (A) / 5% acetonitrile + 0.05% TFA (B), 1–40 min 5% A / 95% B, 40–41 min 5% A / 95% B, 41– 43 min 95% A / 5% B, 43–45 min 95% A / 5% B, flow rate: 10 mL/min, running time: 45 min.

Specific rotation: The specific rotation was measured with a Krüss P3000 polarimeter at 20 °C in MeOH or $CHCl_3$.

Abbreviations: The following abbreviations are used: MeCN = acetonitrile, DCM = dichloromethane, DIPA = diisopropylamine, DCC = *N,N'*-dicyclohexylcarbodiimide, DMP = Dess-Martin periodinane, EtOAc = ethyl acetate, LDA = lithium diisopropylamide, *n*-BuLi = *n*-butyl lithium, THF = tetrahydrofuran, TLC = thin layer chromatography, rt = room temperature, DMF = dimethylformamide, HOBt = 1-dydroxybenzotriazole, DMAP = 4-dimethylaminopyridine, PIFA = (bis(trifluoroacetoxy)iodo)benzene, TMP = 2,2,6,6-tetramethylpiperidine, Pfp = pentafluorophenol

1.2 Biochemistry/Molecular Biology

PD-10 columns and Vivaspin 2 Hydrosart membrane columns (30,000 MWCO) were purchased from VWR. Recombinant production and purification of SorbC was conducted based on previously reported work.[1,2] Final protein concentrations were determined photometrically using the Nanophotometer 330 (Implen) at 280 nm using the extinction coefficient of SUMO-SorbC ε(280 nm) = 52410 $M^{-1}cm^{-1}$. Protein production, enrichment and purification were monitored by SDS-PAGE analysis (BioRad Mini Protean® Tetra System) using Unstained Protein MW Marker (Thermo Scientific). All buffers consisted of 50 mM phosphate at pH 7.5, 250 mM NaCl, and 10% glycerol) with changing concentrations of imidazole (buffer A: 20 mM; buffer B: 250 mM; buffer C: no imidazole). The enzymatic oxidative dearomatization reactions were performed in phosphate buffer (50 mM, pH 8.0).

2. Experimental Procedures

2.1 Heterologous expression of the enzyme SorbC

Starter cultures for SorbC production were prepared from a cryo stock of recombinant *E. Coli* SoluBL21 carrying a pET28(a) vector containing SUMO-SorbC. They were grown at 37°C in 2xYT medium containing 50 µg/ml kanamycin sulfate overnight. These starter cultures were used to inoculate 2xYT (1:100) supplemented with kanamycin. When an OD_{600} = 0.6 was reached, protein production was induced by adding 0.25 mM isopropyl-β-D-thiogalactopyranosid (IPTG), followed by an overnight incubation at 20°C with shaking (180 rpm). The cells were harvested by centrifugation and re-suspended in buffer A. After sonication on ice and subsequent centrifugation (10,000 rpm, 4°C, 30 min), the supernatant was incubated with High Density Nickel Agarose (Jena Bioscience) with moderate shaking for 2 h on ice. This suspension was applied onto a column, which was washed with buffer A. Finally, the protein was eluted with buffer B. A PD-10 column (GE Healthcare) was used to exchange the eluate's buffer to storage buffer C. The resulting purity of SorbC was sufficient for the desired application. Further washing steps resulted in lower yields of SorbC and did not improve catalytic activity. All buffers consisted of 50 mM phosphate at pH 7.5, 250 mM NaCl, and 10% glycerol) with changing concentrations of imidazole (buffer A: 20 mM; buffer B: 250 mM; buffer C: no imidazole). The enzymatic oxidative dearomatization reactions were performed in phosphate buffer (50 mM, pH 8.0).

2.2 2,4-Dihydroxy-3-methylbenzaldehyde (38)

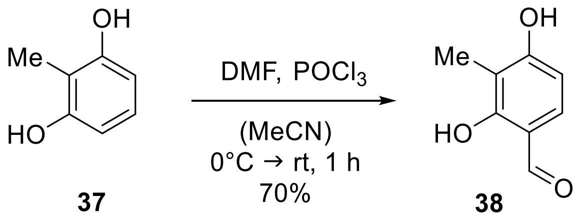

To a solution of DMF (40.4 mL, 38.3 g, 524 mmol, 1.3 eq.) in dry MeCN (150 mL), a solution of $POCl_3$ (41.3 mL, 67.9 g, 443 mmol, 1.1 eq.) in dry MeCN (50.0 mL) was added dropwise over 5 min. After stirring for 30 min at rt, the reaction mixture was cooled to 0 °C. 2-Methylresorcinol (**37**) (5.0 g, 403 mmol, 1.0 eq.) dissolved in dry MeCN (7.50 mL) was added dropwise over 30 min and subsequently stirred at rt for 1 h. The formed aldehyde **38** was filtered off, recrystallized from H_2O, and finally obtained as white crystals (42.9 g, 282 mmol, 70%). ^{1}H-NMR (300 MHz, Acetone-d_6) δ = 11.78 (s, 1H), 9.71 (s, 1H), 7.41 (d, J = 8.5 Hz, 1H), 6.60 (d, J = 8.5 Hz, 1H), 2.06 (s, 3H) ppm. ^{13}C-NMR (75 MHz, Acetone-d_6) δ = 196.0, 163.9, 163.0, 134.1, 115.5, 111.7, 108.8, 7.4 ppm. HRMS (ESI+): m/z = 153.0543 $[M+H]^+$, calc.: 153.0546. The spectroscopic data are in agreement with the literature.[1]

2.3 2,4-Dimethylresorcinol (39)

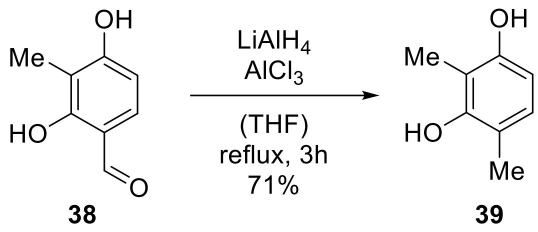

Under an argon atmosphere at 0°C, $AlCl_3$ (17.5 g, 131 mmol, 2.5 eq.) and $LiAlH_4$ (5.0 g, 131 mmol, 2.5 eq.) were carefully added to a solution of aldehyde **38** (8.0 g, 53.6 mmol, 1.0 eq.) in dry THF (500 mL). The resulting suspension was heated under reflux for 3 h. Subsequently, cooling to 0°C allowed quenching of the remaining reagents with 1 M aq. HCl (200 mL) and extraction with Et_2O (3 x 200 ml). The combined organic layers were dried over $MgSO_4$, filtered and the solvent was evaporated under reduced pressure. Purification of the raw product by column chromatography (silica gel, *n*-pentane/EtOAc = 4:1, R_f = 0.52) afforded **39** as white solid (5.20 g, 37.6 mmol, 72%). ^{1}H-NMR (300 MHz, DMSO-d_6) δ = 8.81 (s, 1H), 7.95 (s, 1H), 6.64 (d, *J* = 8.1 Hz, 1H), 6.22 (d, *J* = 8.1 Hz, 1H), 2.04 (s, 3H), 1.97 (s, 3H) ppm. ^{13}C-NMR (75 MHz, DMSO-d_6) δ = 154.1, 153.7, 126.9, 114.6, 110.9, 106.1, 16.2, 9.1 ppm. HRMS (ESI+): m/z = 139.0781 $[M+H]^+$, calc.: 139.0754. The spectroscopic data are in agreement with the literature.[1]

2.4 Sorbicillin (1)

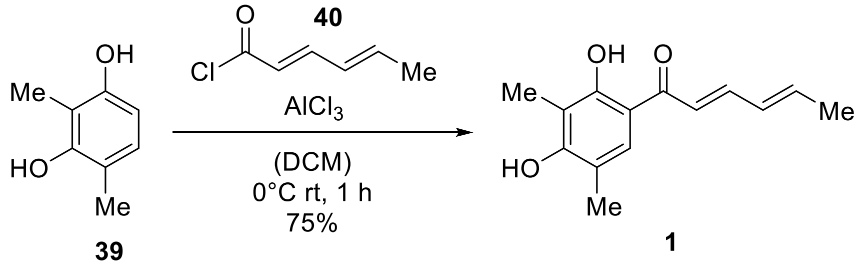

Under an argon atmosphere, 2,4-dimethylresorcinol (**39**) (800 mg, 5.79 mmol, 1.0 eq.) was dissolved in dry DCM (170 mL). At 0°C, first sorbic acid chloride (**40**) (751 mg, 5.79 mmol, 1.0 eq.) followed by $AlCl_3$ (849 mg 6.37 mmol, 1.1 eq.) were added. After stirring at rt for 1 h, 1 M aq. HCl (50.0 mL) was added to the reaction mixture followed by extraction with DCM (3 x 100 mL). The combined organic layers were dried over $MgSO_4$, filtered and the solvent was evaporated under reduced pressure. The raw product was purified by MPLC (column: Reveleris C18- 40g, isocratic, H_2O + 0.05% TFA / MeCN + 0.05% TFA = 50%/50%, flow rate: 30 mL/min, retention time **1**: 15 min, detection wavelength: 220 nm) and afforded **1** as yellow crystals (1.00 g, 4.28 mmol, 74%). ^{1}H-NMR (300 MHz, MeOD) δ = 7.56 (s, 1H), 7.41 (dd, *J* = 14.8, 10.7 Hz, 1H), 7.12 (d, *J* = 14.8 Hz, 1H), 6.56 – 6.18 (m, 2H), 2.19 (s, 3H), 2.08 (s, 3H), 1.91 (d, *J* = 6.6 Hz, 3H) ppm. ^{13}C-NMR (75 MHz, $CDCl_3$) δ = 192.7, 162.7, 158.9, 144.7, 141.3, 130.7, 128.9, 122.0, 114.5, 113.7, 110.5, 19.1, 15.8, 7.7 ppm. HRMS (ESI+): m/z = 233.1171 $[M+H]^+$, calc.: 233.1172. The spectroscopic data are in agreement with the literature.[1]

2.5 General procedure for the peptide coupling of fumaric acid derivatives with amino acids

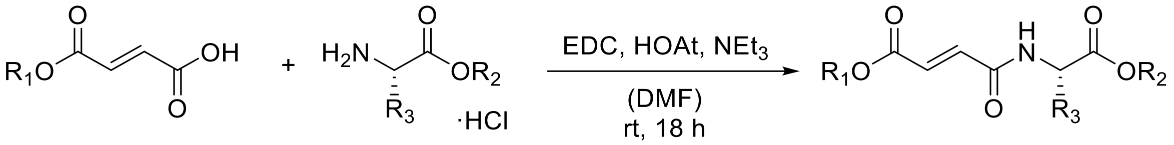

Fumaric acid derivative (1.1 eq.), HOAt (1.2 eq.), NEt_3 (3 eq.) and EDC·HCl (1.2 eq.) were dissolved in DMF (0.2 mmol amino acid/mL). After 5 min, amino acid hydrochloride (1.0 eq.) was added and the reaction mixture was stirred for 18 h at rt. H_2O (5 x volume of DMF) was added and the reaction mixture was extracted with EtOAc (3x). The combined organic layers were washed with brine, dried over $MgSO_4$, filtered and the solvent evaporated under reduced pressure. The crude product was purified by column chromatography. Applied gradients are indicated in the respective section.

2.5.1 Ethyl fumarate-*L*-Ala-O*t*Bu (23)

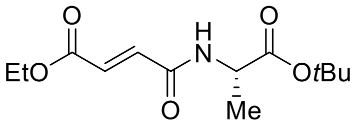

23

23 was synthesized according to the general procedure 2.5. Purification by column chromatography (silica gel, cyclohexane/EtOAc 3:1, R_f = 0.33) afforded **23** as colorless oil (1.45 g, 5.34 mmol, 97%). ^{1}H-NMR (300 MHz, $CDCl_3$) δ = 6.93 (d, *J* = 15.4 Hz, 1H), 6.80 (d, *J* = 15.4 Hz, 1H), 4.54 (pp, *J* = 7.1 Hz, 1H), 4.24 (q, *J* = 7.1 Hz, 2H), 1.47 (s, 9H), 1.41 (d, *J* = 7.1 Hz, 3H), 1.30 (t, *J* = 7.1 Hz, 3H) ppm. ^{13}C-NMR (75 MHz, $CDCl_3$) δ = 172.0, 165.6, 163.1, 136.2, 130.8, 82.5, 61.3, 49.0, 28.0, 18.6, 14.2 ppm. HRMS (ESI+): m/z = 294.1314 $[M+Na]^+$, calc.: 294.1312. $[\alpha]_D^{20}$ = -22 (*c* = 1.76 in MeOH).

2.5.2 Ethyl fumarate-*L*-Ala-OMe (29)

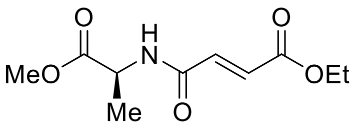

29

29 was synthesized according to the general procedure 2.5. Purification by column chromatography (silica gel, cyclohexane/EtOAc = 1:1, R_f = 0.53) afforded **29** as colorless oil (2.15 g, 9.37 mmol, 90%). ^{1}H-NMR (300 MHz, $CDCl_3$) δ = 7.01 (d, *J* = 15.4 Hz, 1H), 6.79 (d, *J* = 15.4 Hz, 1H), 4.65 (t, *J* = 7.2 Hz, 1H), 4.20 (q, *J* = 7.2 Hz, 2H), 3.73 (s, 3H), 1.41 (d, *J* = 7.2 Hz, 3H), 1.26 (t, *J* = 7.2 Hz, 3H) ppm. ^{13}C-NMR (75 MHz, $CDCl_3$) δ = 173.3, 165.7, 163.3, 136.0, 130.8, 61.3, 52.7, 48.4, 18.2, 14.1 ppm. HRMS (ESI+): m/z = 230.1025 $[M+H]^+$, calc.: 230.1023. $[\alpha]_D^{20}$ = -50 (*c* = 5.12 in MeOH).

2.5.3 Ethyl fumarate-*L*-Abu-OMe (41)

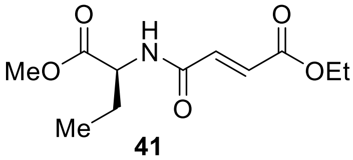

41

41 was synthesized according to the general procedure 2.5. Purification by column chromatography (silica gel, cyclohexane/EtOAc = 2:1, R_f = 0.36) afforded **41** as colorless oil (1.35 g, 5.55 mmol, 85%). ^{1}H-NMR (300 MHz, $CDCl_3$) δ = 7.04 (d, *J* = 15.4 Hz, 1H), 6.80 (d, *J* = 15.4 Hz, 1H), 4.65 (q, *J* = 6.9 Hz, 1H), 4.22 (q, *J* = 7.2 Hz, 2H), 3.74 (s, 3H), 1.99 – 1.84 (m, 1H), 1.83 – 1.64 (m, 1H), 1.27 (t, *J* = 7.2 Hz, 3H), 0.90 (t, *J* = 8.0 Hz, 3H) ppm. ^{13}C-NMR (75 MHz, $CDCl_3$) δ = 172.7, 165.7, 163.5, 136.1, 130.9, 61.3, 53.6, 52.6, 25.6, 14.2, 9.6 ppm. HRMS (ESI+): m/z = 244.1180 $[M+H]^+$, calc.: 244.1179, $[\alpha]_D^{20}$ = -38 (*c* = 1.55 in MeOH).

2.5.4 Ethyl fumarate-Nva-OMe (42)

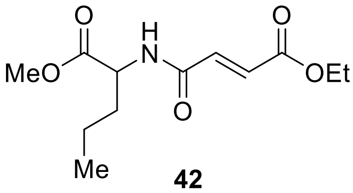

42

42 was synthesized according to the general procedure 2.5. Purification by column chromatography (silica gel, cyclohexane/EtOAc = 2:1, R_f = 0.40) afforded **42** as colorless oil (1.36 g, 5.29 mmol, 82%). ^{1}H-NMR (300 MHz, $CDCl_3$) δ = 7.03 (d, *J* = 15.4 Hz, 1H), 6.80 (d, *J* = 15.4 Hz, 1H), 4.69 (td, *J* = 7.6, 5.1 Hz, 1H), 4.21 (q, *J* = 7.2 Hz, 2H), 3.73 (s, 3H), 1.90 – 1.75 (m, 1H), 1.75 – 1.57 (m, 1H), 1.42 – 1.31 (m, 2H), 1.27 (t, *J* = 7.2 Hz, 3H), 0.89 (t, *J* = 7.3 Hz, 3H) ppm. ^{13}C-NMR (75 MHz, $CDCl_3$) δ = 173.0, 165.7, 163.47, 136.1, 130.9, 61.3, 52.6, 52.4, 34.5, 18.7, 14.2, 13.7 ppm. HRMS (ESI+): m/z = 258.1339 $[M+H]^+$, calc.: 258.1336.

2.5.5 Ethyl fumarate-*L*-Leu-OMe (43)

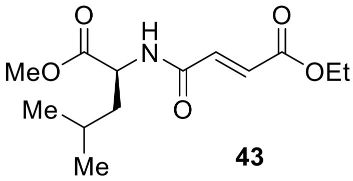

43

43 was synthesized according to the general procedure 2.5. Purification by column chromatography (silica gel, cyclohexane/EtOAc = 3:1, R_f = 0.27) afforded **43** as colorless oil (1.51 g, 5.57 mmol, 86%). ^{1}H-NMR (300 MHz, $CDCl_3$) δ = 7.02 (d, *J* = 15.4 Hz, 1H), 6.86 (d, *J* = 8.4 Hz, 1H, NH), 6.80 (d, *J* = 15.4 Hz, 1H), 4.70 (td, *J* = 8.5, 5.0 Hz, 1H), 4.22 (q, *J* = 7.1 Hz, 2H), 3.72 (s, 3H), 1.72 – 1.46 (m, 3H), 1.28 (t, *J* = 7.2 Hz, 3H), 0.92 (d, *J* = 6.2 Hz, 3H), 0.91 (d, *J* = 6.2 Hz, 3H) ppm. ^{13}C-NMR (75 MHz, $CDCl_3$) δ = 173.4, 165.8, 163.6, 136.1, 130.9, 61.3, 52.5, 51.1, 41.5, 25.0, 22.8, 21.9, 14.2 ppm. HRMS (ESI+): m/z = 272.1492 $[M+H]^+$, calc.: 272.1492, $[\alpha]_D^{20}$ = -39 (*c* = 2.28 in MeOH).

2.5.6 Ethyl fumarate-*L*-Ile-OMe (44)

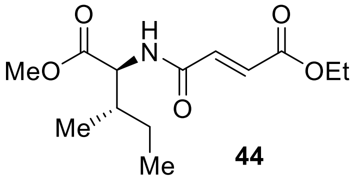

44

44 was synthesized according to the general procedure 2.5. Purification by column chromatography (silica gel, cyclohexane/EtOAc = 3:1, R_f = 0.28) afforded **44** as colorless oil (1.65 g, 6.08 mmol, 93%). ^{1}H-NMR (300 MHz, $CDCl_3$) δ = 7.06 (d, *J* = 15.4 Hz, 1H), 6.90 (d, *J* = 9.2 Hz, 1H), 6.80 (d, *J* = 15.4 Hz, 1H), 4.68 (dd, *J* = 8.7, 5.2 Hz, 1H), 4.22 (q, *J* = 7.2 Hz, 2H), 3.73 (s, 3H), 1.97 – 1.83 (m, 1H), 1.52 – 1.35 (m, 1H), 1.27 (t, *J* = 7.2 Hz, 3H), 1.24 – 1.09 (m, 1H), 0.89 (t, *J* = 7.4 Hz, 3H), 0.89 (d, *J* = 6.9 Hz, 3H) ppm. ^{13}C-NMR (75 MHz, $CDCl_3$) δ = 172.4, 165.7, 163.5, 136.2, 130.9, 61.3, 56.8, 52.4, 38.1, 25.3, 15.4, 14.2, 11.6 ppm. HRMS (ESI+): m/z = 272.1490 $[M+H]^+$, calc.: 272.1492, $[\alpha]_D^{20}$ = -14 (*c* = 1.91 in MeOH).

2.6 Ethyl fumarate-*L*-Ala-OH (24)

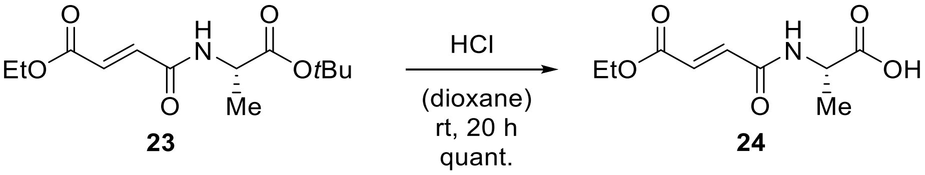

23 (2.17 g, 7.80 mmol, 1.0 eq.) was dissolved in 4 M HCl in dioxane (5 mL, 20 mmol, 2.6 eq.) and stirred at rt for 20 h. H_2O (50.0 mL) was added, and the reaction mixture was extracted with EtOAc (3 x 50.0 mL). The combined organic layers were washed with brine, dried over $MgSO_4$, filtered and the solvent evaporated under reduced pressure to give **24** as white solid (1.68 g, 7.80 mmol, quant.). mp: 27-29°C. ^{1}H-NMR (300 MHz, DMSO-d_6) δ = 8.85 (d, *J* = 7.3 Hz, 1H), 7.07 (d, *J* = 15.5 Hz, 1H), 6.58 (d, *J* = 15.5 Hz, 1H), 4.29 (pp, *J* = 7.3 Hz, 1H), 4.19 (q, *J* = 7.1 Hz, 2H), 1.31 (d, *J* = 7.3 Hz, 3H), 1.24 (t, *J* = 7.1 Hz, 3H) ppm. ^{13}C-NMR (75 MHz, DMSO-d_6) δ = 173.6, 165.0, 162.5, 137.0, 128.9, 60.7, 47.8, 17.1, 14.0 ppm. HRMS (ESI+): m/z = 238.0685 $[M+Na]^+$, calc.: 238.0686, $[\alpha]_D^{20}$ = -26 (*c* = 3.33 in MeOH).

2.7 General procedure for the saponification of methyl-/ethylesters

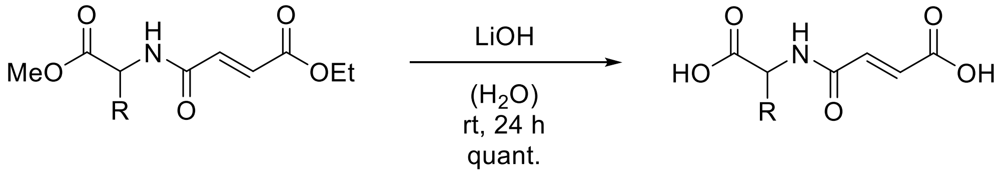

Fumaryl amino acid diester (1.0 eq.) was dissolved in THF (1-5 mL). A solution of LiOH (0.1 M, 4.0 eq.) in H_2O was added and the reaction mixture stirred for 24 h at rt. The reaction mixture was then acidified with 1 M aq. HCl until pH = 1 was reached and extracted with EtOAc (4 x). The combined organic layers were dried over $MgSO_4$, filtered and the solvent evaporated under reduced pressure.

2.7.1 *L*-Ala-fumaric acid (30)

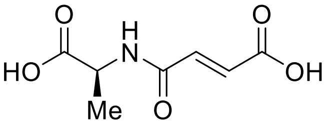

30

30 was synthesized according to the general procedure 2.7 and afforded **30** as white solid (1.22 g, 6.54 mmol, quant.). mp: 204°C (dec.). ^{1}H-NMR (300 MHz, DMSO-d_6) δ = 12.78 (s, 2H), 8.81 (d, *J* = 7.3 Hz, 1H), 6.98 (d, *J* = 15.5 Hz, 1H), 6.53 (d, *J* = 15.5 Hz, 1H), 4.29 (pp, *J* = 7.3 Hz, 1H), 1.30 (d, *J* = 7.3 Hz, 3H) ppm. ^{13}C-NMR (75 MHz, DMSO-d_6) δ = 173.7, 166.4, 162.9, 136.5, 130.1, 47.9, 17.1 ppm. HRMS (ESI+): m/z = 188.0598 $[M+H]^+$, calc.: 188.0553, $[\alpha]_D^{20}$ = -35 (*c* = 1.72 in MeOH).

2.7.2 *L*-Abu-fumaric acid (45)

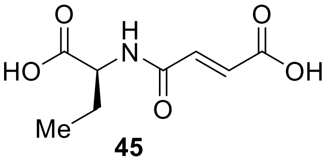

45

45 was synthesized according to the general procedure 2.7 and afforded **45** as white solid (827 mg, 4.11 mmol, quant.). mp: 176-178°C. ^{1}H-NMR (300 MHz, DMSO-d_6) δ = 12.78 (s, 2H), 8.73 (d, *J* = 7.8 Hz, 1H), 7.05 (d, *J* = 15.5 Hz, 1H), 6.53 (d, *J* = 15.5 Hz, 1H), 4.22 (td, *J* = 8.1, 5.1 Hz, 1H), 1.82 – 1.57 (m, 2H), 0.89 (t, *J* = 7.4 Hz, 3H) ppm. ^{13}C-NMR (75 MHz, DMSO-d_6) δ = 173.1, 166.4, 163.2, 136.6, 130.1, 53.6, 24.3, 10.3 ppm. HRMS (ESI+): m/z = 202.0713 $[M+H]^+$, calc.: 202.0710, $[\alpha]_D^{20}$ = -21 (*c* = 1.46 in MeOH).

2.7.3 Nva-fumaric acid (46)

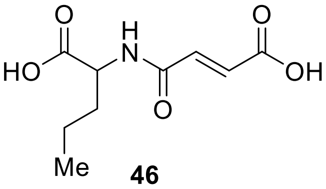

46

46 was synthesized according to the general procedure 2.7 and afforded **46** as white solid (836 mg, 3.89 mmol, quant.). mp: 202°C (dec.). ^{1}H-NMR (300 MHz, DMSO-d_6) δ = 12.78 (s, 2H), 8.74 (d, *J* = 7.7 Hz, 1H), 7.03 (d, *J* = 15.5 Hz, 1H), 6.53 (d, *J* = 15.5 Hz, 1H), 4.32 – 4.22 (m, 1H), 1.77 – 1.53 (m, 2H), 1.41 – 1.26 (m, 2H), 0.87 (t, *J* = 7.3 Hz, 3H) ppm. ^{13}C-NMR (75 MHz, DMSO-d_6) δ = 173.3, 166.4, 163.1, 136.5, 130.1, 51.9, 33.0, 18.6, 13.4 ppm. HRMS (ESI+): m/z = 216.0870 $[M+H]^+$, calc.: 216.0866.

2.7.4 *L*-Leu-fumaric acid (47)

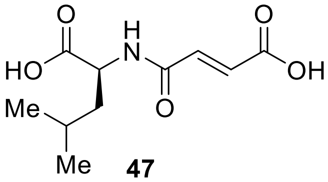

47

47 was synthesized according to the general procedure 2.7 and afforded **47** as white solid (845 mg, 3.68 mmol, quant.). mp: 143-144°C. ^{1}H-NMR (300 MHz, DMSO-d$_6$) δ = 12.76 (s, 2H), 8.75 (d, *J* = 8.0 Hz, 1H), 7.00 (d, *J* = 15.5 Hz, 1H), 6.53 (d, *J* = 15.5 Hz, 1H), 4.29 (t, *J* = 7.4 Hz, 1H), 1.72 – 1.46 (m, 3H), 0.90 (d, *J* = 6.3 Hz, 3H), 0.85 (d, *J* = 6.3 Hz, 3H) ppm. ^{13}C-NMR (75 MHz, DMSO-d$_6$) δ = 173.6, 166.4, 163.1, 136.4, 130.2, 50.6, 39.9, 24.4, 22.8, 21.3 ppm. HRMS (ESI+): m/z = 230.1024 $[M+H]^+$, calc.: 230.1023, $[\alpha]_D^{20}$ = -25 (*c* = 1.57 in MeOH).

2.7.5 *L*-Ile-fumaric acid (48)

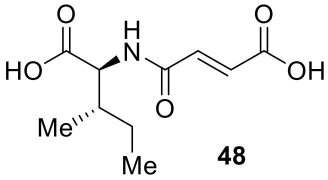

48

48 was synthesized according to the general procedure 2.7 and afforded **48** as white solid (844 mg, 3.68 mmol, quant.). mp: 137-140°C. ^{1}H-NMR (300 MHz, DMSO-d$_6$) δ = 12.78 (s, 2H), 8.64 (d, *J* = 8.5 Hz, 1H), 7.14 (d, *J* = 15.4 Hz, 1H), 6.53 (d, *J* = 15.4 Hz, 1H), 4.28 (dd, *J* = 8.4, 5.9 Hz, 1H), 1.89 – 1.66 (m, 1H), 1.53 – 1.31 (m, 1H), 1.31 – 1.10 (m, 1H), 0.87 (d, *J* = 6.9 Hz, 3H), 0.85 (t, *J* = 7.4 Hz, 3H) ppm. ^{13}C-NMR (75 MHz, DMSO-d$_6$) δ = 172.6, 166.5, 163.3, 136.7, 130.1, 56.7, 36.4, 24.7, 15.6, 11.3 ppm. HRMS (ESI+): m/z = 230.1030 $[M+H]^+$, calc.: 230.1023, $[\alpha]_D^{20}$ = +7 (*c* = 1.78 in MeOH).

2.8 2,2,2-Trifluroacetyl-*L*-Ala-OH (13)

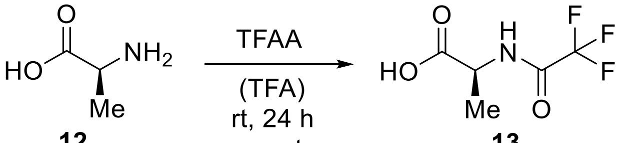

12 **13**

To a solution of *L*-alanine (**12**) (2.50 g, 28.1 mmol, 1.0 eq.) in TFA (11 mL), TFAA (7.80 mL, 11.8 g, 56.1 mmol, 2.0 eq.) was added dropwise at 0°C. The reaction mixture was stirred at rt for 24 h. After evaporating the solvent under reduced pressure, H_2O (20 mL) was added and the aqueous solution was lyophilized to afford **13** as white solid (5.19 g, 28.1 mmol, quant.). ^{1}H-NMR (300 MHz, DMSO-d$_6$) δ = 12.63 (bs, 1H), 9.66 (d, *J* = 7.4 Hz, 1H), 4.31 (pp, *J* = 7.3 Hz, 1H), 1.36 (d, *J* = 7.3 Hz, 3H) ppm. ^{13}C-NMR (75 MHz, DMSO-d$_6$) δ = 172.5, 156.2 (q, *J* = 36.6 Hz), 115.8 (q, *J* = 287.9 Hz), 48.2, 16.2 ppm. HRMS (ESI-): m/z = 184.0233 $[M-H]^-$, calc.: 184.0227. The spectroscopic data are in agreement with the literature.[3]

2.9 2,2,2-Trifluroacetyl-*L*-Ala-Pfp (49)

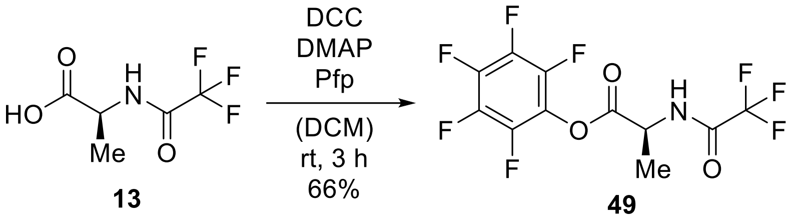

2,2,2-Trifluoroactyl-*L*-Ala-OH (**13**) (300 mg, 1.62 mmol, 1.2 eq.), DMAP (10 mg, 0.08 mmol, 5 mol%) and pentafluorophenol (249 mg, 1.35 mmol, 1.0 eq.) were dissolved in DCM (30 mL). At 0°C, DCC (335 mg, 1.62 mmol, 1.2 eq.) was added to the solution and the reaction mixture was stirred at rt for 3 h. The resulting suspension was filtered, and the solvent evaporated under reduced pressure. The obtained residue was dissolved in DCM and washed with 1 M aq. HCl and brine, dried over $MgSO_4$ and the solvent evaporated under reduced pressure. The crude product was purified by column chromatography and afforded **49** as white solid (375 mg, 1.07 mmol, 66%). mp: 85-87°C. ^{1}H-NMR (300 MHz, Acetone-d_6) δ = 9.18 (s, 1H), 5.06 – 4.92 (m, 1H), 1.73 (d, *J* = 7.3 Hz, 3H) ppm. ^{13}C-NMR (75 MHz, Acetone-d_6) δ = 168.7, 157.9 (d, *J* = 37.4 Hz), 144.2 – 136.3 (m, arom. -CF), 117.1 (q, *J* = 287.1), 49.5, 16.6 ppm. HRMS (ESI+): m/z = 352.0215 $[M+H]^+$, calc.: 352.0214, $[\alpha]_D^{20}$ = -12 (*c* = 0.81 in $CHCl_3$).

2.10 General procedure for the esterification of sorbicillin with corresponding acids

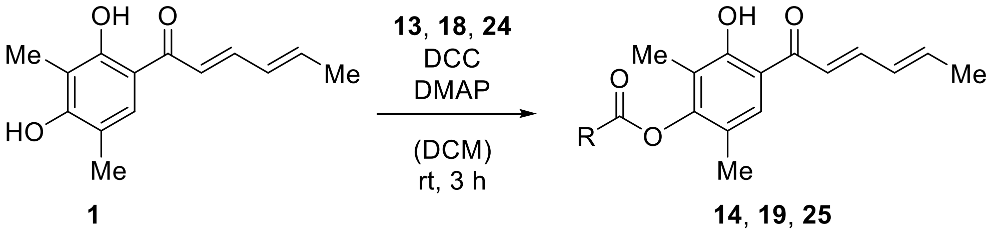

Sorbicillin (**1**) (1.0 eq.), DMAP (5 mol%) and corresponding acid (1.2 eq.) were dissolved in DCM (0.2 mmol/mL). At 0°C, DCC (1.2 eq.) was added to the solution and the reaction mixture was stirred for 3 h at rt. The resulting suspension was filtered, and the solvent evaporated under reduced pressure. The obtained residue was dissolved in DCM and washed with 1 M aq. HCl and brine, dried over $MgSO_4$ and the solvent evaporated under reduced pressure. The crude product was purified by column chromatography.

2.10.1 Sorbicillin-*L*-Ala-ethylfumarate (25)

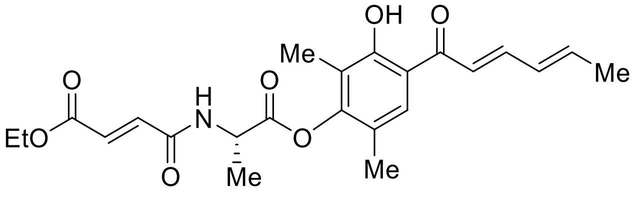

25

25 was synthesized according to the general procedure 2.9. Purification of the crude product by column chromatography (silica gel, cyclohexane/EtOAc = 3:1, R_f = 0.20) afforded **25** as yellow solid

(540 mg, 1.26 mmol, 37%). mp: 149°C (dec.). ^{1}H-NMR (600 MHz, Acetone-d_6) δ = 13.40 (s, 1H), 7.84 (s, 1H), 7.52 (dd, *J* = 14.8, 10.1 Hz, 1H), 7.31 (d, *J* = 14.8 Hz, 1H), 7.11 (d, *J* = 15.5 Hz, 1H), 6.74 (d, *J* = 15.5 Hz, 1H), 6.53 – 6.39 (m, 2H), 4.84 (q, *J* = 7.3 Hz, 1H), 4.22 (q, *J* = 7.1 Hz, 2H), 2.14 (s, 3H), 2.04 (s, 1H), 1.91 (d, *J* = 5.8 Hz, 3H), 1.69 (d, *J* = 7.3 Hz, 3H), 1.28 (t, *J* = 7.1 Hz, 3H) ppm. ^{13}C-NMR (151 MHz, Acetone-d_6) δ = 194.7, 170.6, 165.8, 164.1, 161.8, 154.5, 146.6, 143.1, 136.9, 131.5, 130.9, 129.7, 122.9, 121.9, 120.2, 118.3, 61.6, 49.5, 19.0, 17.4, 16.1, 14.4, 9.0 ppm. HRMS (ESI+): m/z = 452.1677 $[M+Na]^+$, calc.: 452.1680, $[\alpha]_D^{20}$ = +5 (*c* = 0.63 in $CHCl_3$).

2.10.2 Sorbicillin-*L*-Ala-TFA (14)

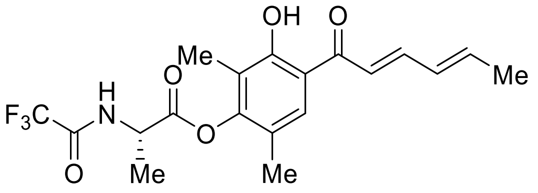

14

14 was synthesized according to the general procedure 2.9. Purification of the crude product by column chromatography (silica gel, pentane/EtOAc = 5:1, R_f = 0.30) afforded **14** as yellow solid (790 mg, 1.98 mmol, 92%). mp: 157°C (dec.) ^{1}H-NMR (600 MHz, Acetone-d_6) δ = 13.41 (s, 1H), 9.09 (d, *J* = 6.1 Hz, 1H), 7.85 (s, 1H), 7.61 – 7.38 (m, 1H), 7.30 (d, *J* = 14.8 Hz, 1H), 6.59 – 6.25 (m, 2H), 5.04 – 4.69 (m, 1H), 2.14 (s, 3H), 2.04 (s, 3H), 1.91 (d, *J* = 5.4 Hz, 3H), 1.76 (d, *J* = 7.4 Hz, 3H) ppm. ^{13}C-NMR (151 MHz, Acetone-d_6) δ = 194.4, 169.2, 161.5, 157.5 (q, *J* = 37.5 Hz), 154.0, 146.4, 142.9, 131.2, 129.5, 122.6, 121.4, 119.8, 118.1, 116.6 (q, *J* = 287.1 Hz), 49.4, 18.7, 16.5, 15.7, 8.6 ppm. HRMS (ESI+): m/z = 422.1181 $[M+Na]^+$, calc.: 422.1186, $[\alpha]_D^{20}$ = -16 (*c* = 1.02 in $CHCl_3$).

2.10.3 Sorbicillin-bromopropanate (19)

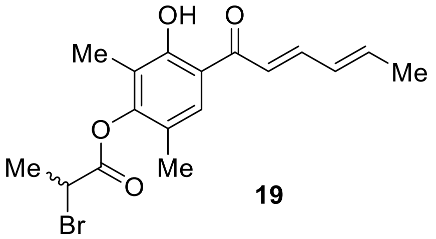

19

19 was synthesized according to the general procedure 2.9. Purification of the crude product by column chromatography (silica gel, pentane/EtOAc = 3:1, R_f = 0.60) afforded **19** as yellow solid (1.50 g, 4.09 mmol, 96%). mp: 106-108°C. ^{1}H-NMR (300 MHz, Acetone-d_6) δ = 13.42 (s, 1H), 7.85 (s, 1H), 7.60 – 7.44 (m, 1H), 7.29 (d, *J* = 14.8 Hz, 1H), 6.56 – 6.37 (m, 2H), 4.98 (q, *J* = 6.8 Hz, 1H), 2.16 (s, 3H), 2.06 (s, 3H), 1.97 (d, *J* = 6.8 Hz, 3H), 1.91 (d, J = 5.3 Hz, 3H) ppm. ^{13}C-NMR (75 MHz, Acetone-d_6) δ = 194.7, 168.1, 162.2, 154.0, 146.8, 143.2, 131.5, 129.9, 122.8, 121.6, 120.3, 118.4, 40.5, 22.1, 19.0, 16.0, 8.9 ppm. HRMS (ESI+): m/z = 367.0558 $[M+H]^+$, calc.: 367.0539.

2.11 Sorbicillin-nitropropanate (49)

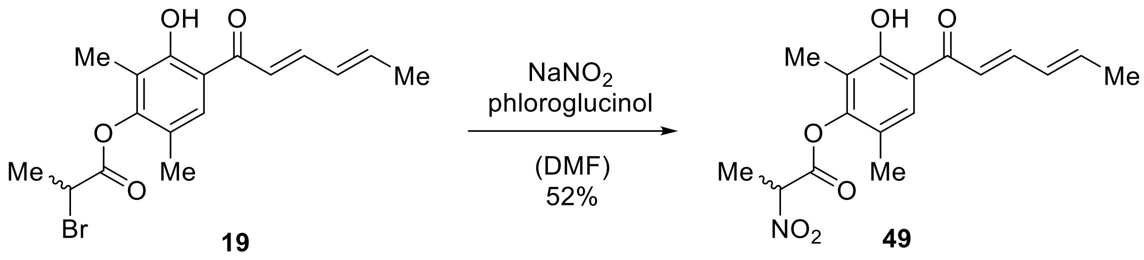

To a solution of $NaNO_2$ (639 mg, 9.26 mmol, 1.7 eq.) and phloroglucinol (618 mg, 4.09 mmol, 0.9 eq.) in DMF (12 mL), **19** (2.00 g, 5.45 mmol, 1.0 eq.) was added at -15°C. The stirred reaction mixture was allowed to warm to 10°C over 3h, then poured into ice-cold water for quenching and extracted with Et_2O (3x). The combined organic layers were washed with brine, dried over $MgSO_4$, filtered and the solvent evaporated under reduced pressure. Purification of the raw product by column chromatography (silica gel, cyclohexane/EtOAc = 3:1, R_f = 0.50) afforded **49** as yellow solid (944 mg, 2.83 mmol, 52%). mp: 108-110°C. ^{1}H-NMR (300 MHz, $CDCl_3$) δ = 13.26 (s, 1H), 7.54 (s, 1H), 7.60 – 7.41 (m, 1H), 6.95 (d, *J* = 14.7 Hz, 1H), 6.44 – 6.26 (m, 2H), 5.49 (q, *J* = 7.1 Hz, 1H), 2.14 (s, 3H), 2.07 (s, 3H), 1.99 (d, *J* = 7.1 Hz, 3H), 1.93 (d, *J* = 5.1 Hz, 3H) ppm. ^{13}C-NMR (75 MHz, $CDCl_3$) δ = 193.7, 162.5, 161.6, 152.3, 146.3, 142.8, 130.6, 128.8, 121.5, 120.1, 119.9, 118.2, 83.0, 19.2, 16.1, 16.0, 8.9 ppm. HRMS (ESI+): m/z = 334.1326 $[M+H]^+$, calc.: 334.1285.

2.12 General procedure for the PIFA mediated oxidative dearomatization of sorbicillin derivatives

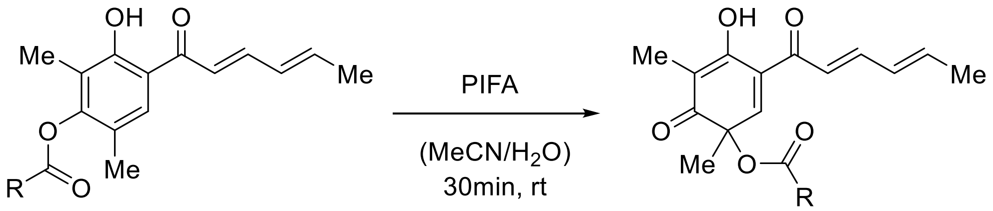

To a solution of sorbicillinol derivative (50mM, 1.0 eq.) in MeCN, PIFA (1.1 eq.) was added at 0°C. The reaction mixture was stirred at rt for 30 min, then diluted with H_2O and extracted with EtOAc (3x). The combined organic layers were washed with brine, dried over $MgSO_4$, filtered and the solvent evaporated under reduced pressure. Since the formed diastereoisomers could not be separated, only HRMS data is provided for the following synthetic intermediates. NMR analysis of the purified mixture of diastereoisomers indicated the expected 1:1 ratio.

2.12.1 Sorbicillinol-*L*-Ala-TFA (15)

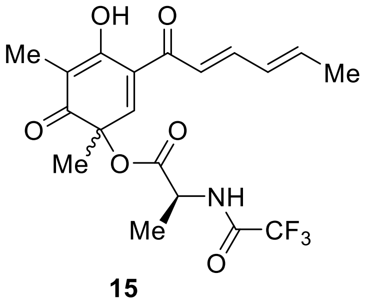

15

15 was synthesized according to the general procedure 2.11. The raw product was purified by MPLC (column: Reveleris C18- 40g, gradient: 0 min 60% H_2O + 0.05% TFA (A) / 40% acetonitrile + 0.05% TFA (B), 0–8 min 70% A / 30% B, 8–28 min 50% A / 50% B, 28-28.1 min 5% A / 95% B, 28.1-38 min 5% A / 95% B, 38-38.1 min 70% A / 30% B, 38-40 min 70% A / 30% B, flow rate: 40 mL/min, running time: 38 min., retention time **15**: 27 min, detection wavelength: 370 nm) and afforded **15** as yellow solid (322 mg, 0.78 mmol, 29%). HRMS (ESI+): m/z = 438.1129 $[M+Na]^+$, calc.: 438.1135.

2.12.2 Sorbicillinol-*L*-Ala-fumarate-ethyl ester (26)

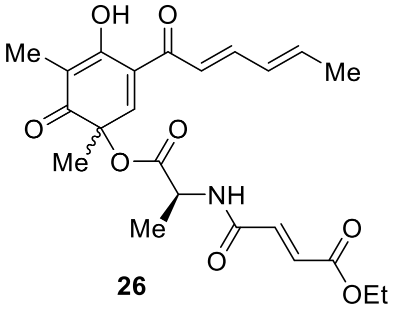

26

26 was synthesized according to the general procedure 2.11. The raw product was purified by MPLC (column: Reveleris C18- 40g, gradient: 0 min 60% H_2O + 0.05% TFA (A) / 40% acetonitrile + 0.05% TFA (B), 0–10 min 60% A / 40% B, 10–20 min 5% A / 95% B, 20-30 min 5% A / 95% B, flow rate: 40 mL/min, running time: 30 min., retention time **26**: 27 min, detection wavelength: 370 nm) and afforded **26** as yellow solid (134 mg, 0.30 mmol, 48%). HRMS (ESI+): m/z = 468.1630 $[M+Na]^+$, calc.: 468.1629.

2.12.3 Sorbicillinol-bromopropanat (20)

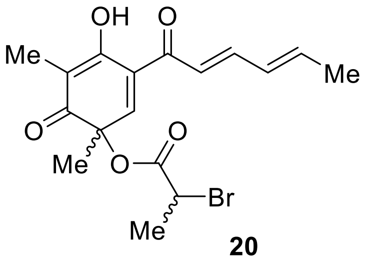

20

20 was synthesized according to the general procedure 2.11. The raw product was purified by MPLC (column: Reveleris C18- 40g, gradient: 0 min 60% H_2O + 0.05% TFA (A) / 40% acetonitrile + 0.05% TFA (B), 0–10 min 60% A / 40% B, 10–20 min 5% A / 95% B, 20-30 min 5% A / 95% B, flow rate: 40 mL/min,

running time: 30 min., retention time **20**: 28 min, detection wavelength: 370 nm) and afforded **20** as yellow solid (121 mg, 0.32 mmol, 32%). HRMS (ESI+): m/z = 383.0488 $[M+H]^+$, calc.: 383.0489.

2.13 Nitrosorbicillactones (21a,b)

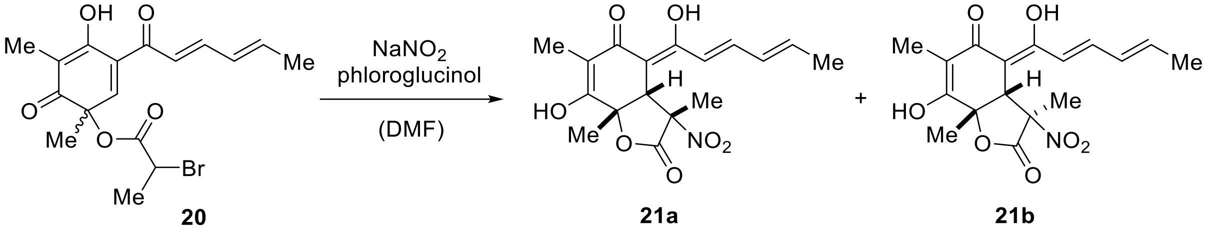

To a solution of $NaNO_2$ (126 mg, 1.82 mmol, 1.7 eq.) and phloroglucinol (122 mg, 0.96 mmol, 0.9 eq.) in DMF (12 mL), **20** (375 mg, 1.07 mmol, 1.0 eq.) was added at -15°C. The stirred reaction mixture was allowed to warm to 10°C over 3h, then poured into ice-cold water for quenching and extracted with Et_2O (3x). The combined organic layers were washed with brine, dried over $MgSO_4$, filtered and the solvent evaporated under reduced pressure. Purification of the raw product by HPLC (column: Eurosphere II 100-5 C18 A, gradient: 0 min 70% H_2O + 0.05% TFA (A) / 30% acetonitrile + 0.05% TFA (B), 0–2 min 70% A / 30% B, 2–5 min 60% A / 40% B, 5-26 min 40% A / 60% B, flow rate: 10 mL/min, running time: 26 min., retention time **21a**: 24.3 min, epimer **21b**: 25.2 min, detection wavelength: 370 nm) afforded **21a** (15 mg, 42.9 µmol, 4%) and epimer **21c** (41 mg, 118 µmol, 11%) as yellow solids.

21a: mp: 133°C (dec.). ^{1}H-NMR (300 MHz, Acetone-d_6) δ = 16.61 (s, 1H), 7.31 (dd, *J* = 14.8, 10.0 Hz, 1H), 6.59 (d, *J* = 14.8 Hz, 1H), 6.40 – 6.16 (m, 2H), 4.29 (s, 1H), 1.86 (s, 3H), 1.86 (d, *J* = 5.5 Hz, 3H), 1.76 (s, 3H), 1.65 (s, 3H) ppm. ^{13}C-NMR (75 MHz, Acetone-d_6) δ = 190.6, 172.3, 168.1, 163.4, 141.4, 139.3, 131.7, 120.8, 110.9, 97.3, 93.7, 82.9, 51.1, 24.5, 20.6, 18.8, 7.3 ppm. HRMS (ESI+): m/z = 350.1238 $[M+H]^+$, calc.: 350.1234.

Epimer **21b**: mp: 105-107°C. ^{1}H-NMR (300 MHz, Acetone-d_6) δ = 16.79 (s, 1H), 7.22 (dd, *J* = 14.8, 9.7 Hz, 1H), 6.32 – 6.08 (m, 2H), 5.87 (d, *J* = 14.8 Hz, 1H), 4.68 (s, 1H), 1.91 (s, 3H), 1.85 (d, *J* = 5.4 Hz, 3H), 1.81 (s, 3H), 1.63 (s, 3H) ppm. ^{13}C-NMR (75 MHz, Acetone-d_6) δ = 190.7, 173.6, 169.4, 163.1, 142.2, 140.1, 131.6, 119.4, 111.5, 97.3, 94.3, 83.3, 52.0, 25.4, 18.8, 16.8, 7.6 ppm. HRMS (ESI+): m/z = 350.1240 $[M+H]^+$, calc.: 350.1234.

2.14 (*rac*)-SorbicillactoneTFA (16a,b)

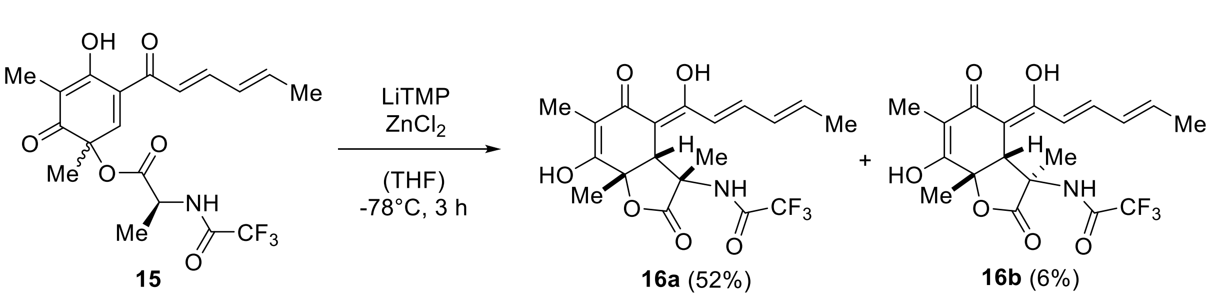

Under an argon atmosphere, *n*-BuLi (1.44 mL, 3.61 mmol, 2.5 M in hexane, 10 eq.) was added dropwise to a solution of TMP (510 mg, 3.61 mmol, 10 eq.) in THF (5 mL) at -78°C. The solution was stirred at 0°C for 10 min and afterwards cooled to -78°C. A solution of **15** (150 mg, 0.36 mmol, 1 eq.) and $ZnCl_2$ (0.7 M in THF, 1.08 mL, 2.1 eq) in THF (5 mL) was added dropwise. After stirring for 3 h at -78°C the reaction mixture was quenched with 4 M HCl in dioxane and extracted with Et_2O (3x). The combined organic

layers were dried over $MgSO_4$, filtered and the solvent evaporated under reduced pressure. The raw product was purified by MPLC (column: Reveleris C18- 40g, gradient: 0 min 70% H_2O + 0.05% TFA (A) / 30% acetonitrile + 0.05% TFA (B), 0–8 min 70% A / 30% B, 8–38 min 50% A / 50% B, flow rate: 40 mL/min, running time: 38 min., retention time **16a**: 30 min, epimer **16b**: 32 min, detection wavelength: 370 nm) and afforded **16a** (78 mg, 0.19 mmol, 52%) and epimer **16b** (9 mg, 0.02 mmol, 6%) as yellow solids.

Epimer **16b**: mp: 123°C (dec.). ^{1}H-NMR (600 MHz, Acetone-d_6) δ = 16.66 (s, 1H), 9.03 (s, 1H), 7.22 (dd, *J* = 14.7, 9.7 Hz, 1H), 6.26 – 6.10 (m, 2H), 5.98 (d, *J* = 14.9 Hz, 1H), 4.22 (s, 1H), 1.84 (d, *J* = 5.3 Hz, 3H), 1.79 (s, 3H), 1.77 (s, 3H), 1.33 (s, 3H) ppm. ^{13}C-NMR (151 MHz, Acetone-d_6) δ = 191.6, 173.3, 171.9, 164.4, 157.2 (q, *J* = 36.8 Hz), 140.8, 138.9, 131.6, 120.5, 116.5 (q, *J* = 284.7 Hz), 111.2, 98.7, 81.1, 62.8, 46.0, 25.8, 18.8, 18.7, 7.5 ppm. HRMS (ESI+): m/z = 438.1133 $[M+Na]^+$, calc.: 438.1135.

16a: for NMR and HRMS see section 2.15.

2.15 (-)SorbicillactoneTFA (16a)

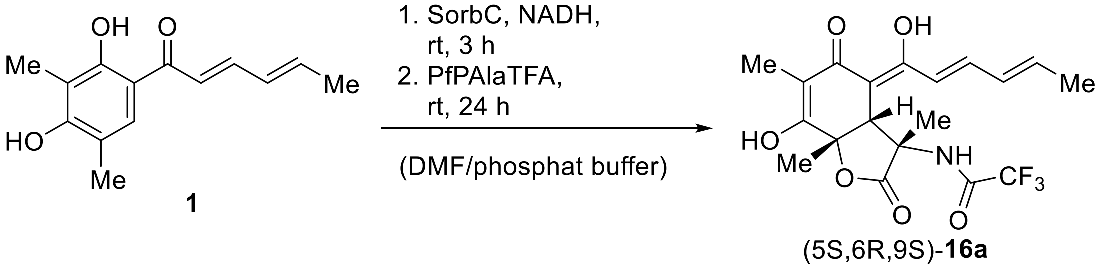

Phosphate buffer (30 mL, 50 mM, pH = 8) and the enzyme SorbC (6.65 mg, 0.11 µmol, 0.1mol%) in phosphate buffer were added to a solution of sorbicillin (**1**) (25.0 mg, 108 µmol, 1 eq.) in DMF (5 mL). The enzymatic reaction was started by the addition NADH (92.6 mg, 129 µmol, 1.2 eq.) dissolved in phosphate buffer (3 mL) and incubated for 3 h at rt. A solution of PfpAlaTFA (49.1 mg, 140 µmol, 1.3 eq in DCM (30 mL) was added and the emulsion was stirred for 12 h. After acidification with aq. HCl (1M), the reaction mixture was extracted with Et_2O (3x). The combined organic layers were dried over $MgSO_4$, filtered and the solvent evaporated under reduced pressure. The raw product was purified by HPLC (column: Eurosphere II 100-5 C18 A, gradient: 0 min 90% H_2O + 0.05% TFA (A) / 10% acetonitrile + 0.05% TFA (B), 0–2 min 90% A / 10% B, 2–2.1 min 75% A / 25% B, 2.1-25 min 40% A / 60% B, 25-27 min 30% A / 70% B, 27.1-30 min 5% A / 95% B, 30-30.1 min 90% A / 10% B, 30-32 min 90% A / 10% B, flow rate: 10 mL/min, running time: 32 min., retention time **16a**: 25.8 min, detection wavelength: 370 nm) and afforded **16a** as yellow solid (8 mg, 19.4 µmol, 18%). mp: 124°C (dec.).^{1}H-NMR (600 MHz, Acetone-d_6) δ = 16.56 (s, 1H), 8.56 (s, 1H), 7.19 (dd, *J* = 14.8, 11.0 Hz, 1H), 6.54 (d, *J* = 14.8 Hz, 1H), 6.36 – 6.26 (m, 1H), 6.22 – 6.11 (m, 1H), 3.85 (s, 1H), 1.83 (dd, *J* = 6.8, 1.3 Hz, 3H), 1.67 (s, 3H), 1.67 (s, 3H), 1.64 (s, 3H) ppm. ^{13}C-NMR (151 MHz, Acetone-d_6) δ = 191.8, 172.8, 171.2, 165.5, 155.8 (q, *J* = 37.4 Hz), 140.1, 138.0, 131.9, 121.6, 116.6 (q, *J* = 288.1 Hz), 110.7, 98.7, 82.0, 61.2, 52.2, 25.2, 25.0, 18.8, 7.2 ppm. HRMS (ESI+): m/z = 438.1132 $[M+Na]^+$, calc.: 438.1135. $[\alpha]_D^{20}$ = −299 (*c* = 0.23, MeOH).

2.16 Sorbicillactone ethyl ester (27a,b)

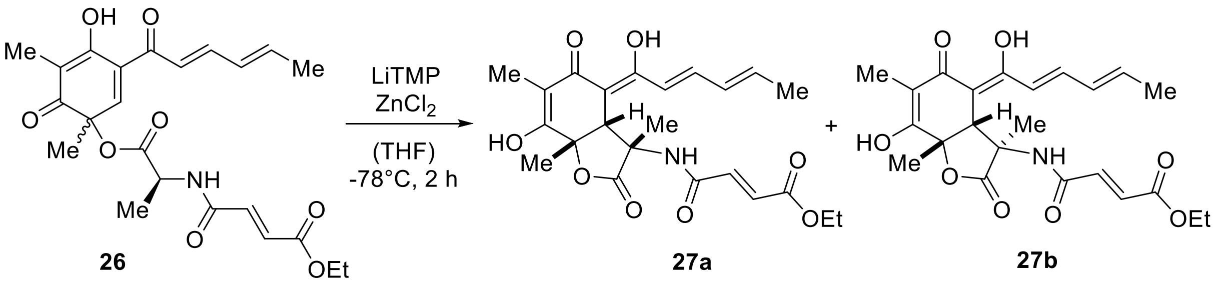

Under an argon atmosphere, *n*-BuLi (808 µL, 2.02 mmol, 2.5 M in hexane, 6 eq.) was added dropwise to a solution of TMP (285 mg, 2.02 mmol, 6 eq.) in THF (3 mL) at -78°C. The solution was stirred at 0°C for 10 min and afterwards cooled to -78°C. A solution of **26** (150 mg, 0.34 mmol, 1 eq.) and $ZnCl_2$ (0.7 M in THF, 490 µL, 2.1 eq) in THF (2 mL) was added dropwise. After stirring for 3 h at -78°C the reaction mixture was quenched with 4 M HCl in dioxane and extracted with Et_2O (3x). The combined organic layers were dried over $MgSO_4$, filtered and the solvent evaporated under reduced pressure. The raw product was purified by HPLC (column: Eurosphere II 100-5 C18 A, gradient: 0 min 80% H_2O + 0.05% TFA (A) / 20% acetonitrile + 0.05% TFA (B), 0–2 min 80% A / 20% B, 2–5 min 70% A / 30% B, 5-36 min 45% A / 55% B, flow rate: 10 mL/min, running time: 35 min., retention time **27a**: 33.2 min, epimer **27b**: 34.1 min, detection wavelength: 370 nm) and afforded **27a** (1 mg, 2.24 µmol, <1%) and epimer **27b** (4 mg, 8.98 µmol, 2%) as yellow solids.

27a: mp: 100°C (dec.). ^{1}H-NMR (600 MHz, Acetone-d_6) δ = 16.51 (s, 1H), 7.73 (s, 1H), 7.18 (dd, *J* = 14.7, 10.9 Hz, 1H), 6.85 (d, *J* = 15.4 Hz, 1H), 6.55 (d, *J* = 14.5 Hz, 1H), 6.52 (d, *J* = 15.4 Hz, 1H), 6.33 – 6.28 (m, 1H), 6.19 – 6.06 (m, 1H), 4.18 (q, *J* = 7.1 Hz, 2H), 3.74 (s, 1H), 1.84 (d, *J* = 5.3 Hz, 3H), 1.64 (s, 3H), 1.58 (s, 3H), 1.56 (s, 3H), 1.25 (t, *J* = 7.1 Hz, 3H) ppm. HRMS (ESI+): m/z = 446.1807 $[M+H]^+$, calc.: 446.1809.

Epimer **27b**: mp: 85°C (dec.) ^{1}H-NMR (600 MHz, Acetone-d_6) δ = 16.67 (s, 1H), 8.23 (s, 1H), 7.14 – 7.09 (m, 1H), 7.07 (d, *J* = 15.5 Hz, 1H), 6.83 (d, *J* = 15.5 Hz, 1H), 6.16 – 6.07 (m, 1H), 6.02 – 5.95 (m, 1H), 5.92 (d, *J* = 14.9 Hz, 1H), 4.36 (s, 1H), 4.24 (q, *J* = 7.1 Hz, 2H), 1.78 (s, 3H), 1.76 (d, *J* = 6.8 Hz, 3H), 1.74 (s, 3H), 1.29 (t, *J* = 7.1 Hz, 3H), 1.25 (s, 3H) ppm. HRMS (ESI+): m/z = 446.1809 $[M+H]^+$, calc.: 446.1809.

2.17 General procedure for the synthesis of azlactones

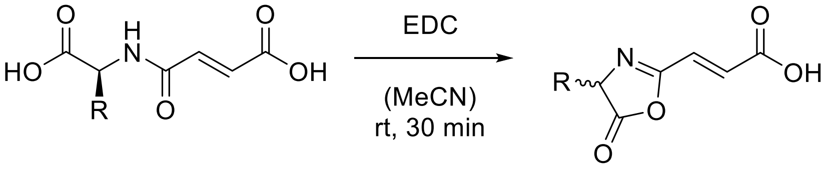

EDC (1.1 eq.) was added to a solution of fumaryl amino acid (133 mM, 1.0 eq.) in MeCN and the reaction mixture stirred for 30min at rt. The whole reaction mixture was used in further steps without any work up or purification.

2.18 General procedure for the enzymatic synthesis of sorbicillactone A and derivatives

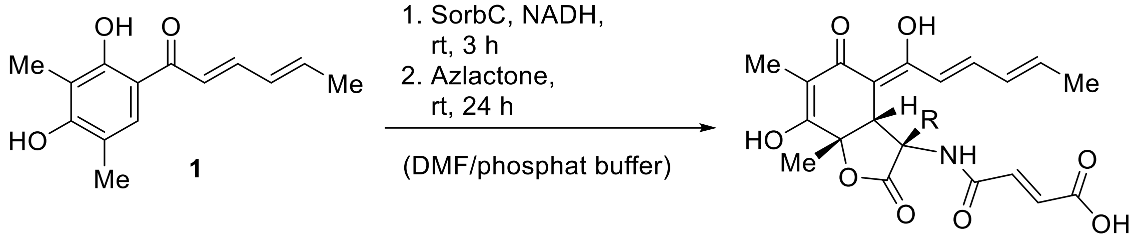

7, 33-36

Phosphate buffer (30 mL, 50 mM, pH = 8) and the enzyme SorbC (6.65 mg, 0.11 µmol, 0.1mol%) in phosphate buffer were added to a solution of sorbicillin (**1**) (25.0 mg, 108 µmol, 1 eq.) in DMF (5 mL). The enzymatic reaction was started by the addition of NADH (92.6 mg, 129 µmol, 1.2 eq.) dissolved in phosphate buffer (3 mL) and incubated for 3 h at rt. Freshly prepared azlactone (1.2 eq.) was added to the enzymatic reaction and the mixture was stirred for 12 h. After acidification with aq. HCl (1M), the reaction mixture was extracted with Et_2O (3x). The combined organic layers were dried over MgSO4, filtered and the solvent evaporated under reduced pressure. The raw product was purified by HPLC (column: Eurosphere II 100-5 C18 A, gradient: 0 min 90% H_2O + 0.05% TFA (A) / 10% acetonitrile + 0.05% TFA (B), 0–2 min 90% A / 10% B, 2–2.1 min 75% A / 25% B, 2.1-25 min 45% A / 55% B, 25-27 min 40% A / 60% B, 27.1-30 min 5% A / 95% B, 30-30.1 min 90% A / 10% B, 30-32 min 90% A / 10% B, flow rate: 10 mL/min, running time: 32 min., detection wavelength: 370 nm)

2.18.1 Sorbicillactone A (7)

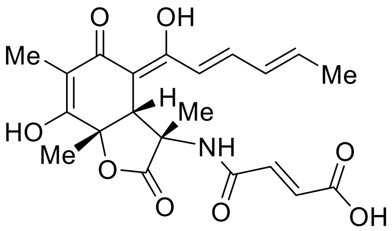

7

7 was synthesized and purified (t_R = 23.3 min) according to the general procedure 2.17, yielding 13 mg (31 µmol, 29%) of a yellow solid. mp: 145°C (dec.). ^{1}H-NMR (600 MHz, Acetone-d_6) δ = 7.73 (s, 1H), 7.18 (dd, *J* = 14.7, 10.8 Hz, 1H), 6.86 (d, *J* = 15.4 Hz, 1H), 6.55 (d, *J* = 15.0 Hz, 1H), 6.53 (d, *J* = 15.4 Hz, 1H), 6.32 (ddd, *J* = 14.7, 11.0, 1.9 Hz, 1H), 6.21 – 6.10 (m, 1H), 3.74 (s, 1H), 1.84 (dd, *J* = 6.9, 1.8 Hz, 3H), 1.64 (s, 3H), 1.59 (s, 3H), 1.56 (s, 3H) ppm. ^{13}C-NMR (151 MHz, Acetone-d_6) δ = 192.2, 173.6, 169.2, 166.3, 165.9, 162.7, 139.6, 137.6, 136.6, 132.0, 131.0, 121.6, 111.1, 99.7, 81.4, 60.6, 52.7, 25.9, 25.3, 18.7, 7.6 ppm. HRMS (ESI+): m/z = 418.1490 $[M+H]^+$, calc.: 418.1496. $[\alpha]_D^{20}$ = −881 (*c* = 0.38 in MeOH). The spectroscopic data are in agreement with the literature.[4,5]

2.18.2 9-Ethyl sorbicillactone A (33)

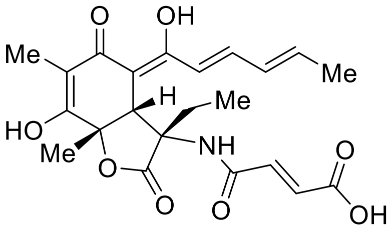

33

33 was synthesized and purified (t_R = 24.0 min) according to the general procedure 2.17, yielding 12 mg (28 µmol, 26%) of a yellow solid. mp: 159°C (dec.). ^{1}H-NMR (600 MHz, Acetone-d_6) δ = 16.49 (s, 1H), 7.68 (s, 1H), 7.16 (dd, *J* = 14.8, 11.0 Hz, 1H), 6.90 (d, *J* = 15.4 Hz, 1H), 6.53 (d, *J* = 15.4 Hz, 1H), 6.41 (d, *J* = 14.8 Hz, 1H), 6.31 (ddd, *J* = 14.6, 10.9, 1.7 Hz, 1H), 6.16 (m, 1H), 3.67 (s, 1H), 1.93 – 1.86 (m, 2H), 1.84 (dd, *J* = 6.9, 1.7 Hz, 3H), 1.65 (s, 3H), 1.59 (s, 3H), 1.12 (t, *J* = 7.3 Hz, 3H) ppm. ^{13}C-NMR (151 MHz, Acetone-d_6) δ = 192.3, 172.5, 170.1, 166.3, 166.1, 163.0, 139.7, 137.8, 136.7, 132.0, 131.1, 121.5, 110.8, 99.6, 81.3, 63.5, 49.9, 30.8, 25.3, 18.7, 8.0, 7.6 ppm. HRMS (ESI+): m/z = 432.1649 $[M+H]^+$, calc.: 432.1653. $[\alpha]_D^{20}$ = −946 (*c* = 1.05 in MeOH).

2.18.3 9-Propyl sorbicillactone A (34)

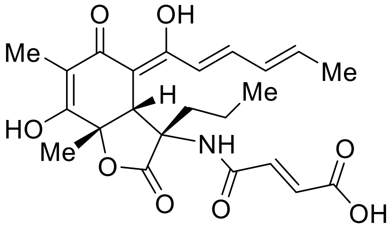

34

34 was synthesized and purified (t_R = 24.9 min) according to the general procedure 2.17, yielding 12 mg (27 µmol, 25%) of a yellow solid. mp: 140°C (dec.). ^{1}H-NMR (600 MHz, Acetone-d_6) δ = 16.50 (s, 1H), 7.67 (s, 1H), 7.17 (dd, *J* = 14.8, 10.9 Hz, 1H), 6.89 (d, *J* = 15.4 Hz, 1H), 6.53 (d, *J* = 15.4 Hz, 1H), 6.40 (d, *J* = 14.8 Hz, 1H), 6.34 – 6.26 (m, 1H), 6.21 – 6.14 (m, 1H), 3.69 (s, 1H), 1.84 (dd, *J* = 6.9, 1.7 Hz, 3H), 1.83 – 1.78 (m, 3H), 1.65 (s, 3H), 1.59 (s, 3H), 1.43 – 1.33 (m, 1H), 0.94 (t, *J* = 7.2 Hz, 3H) ppm. ^{13}C-NMR (151 MHz, Acetone-d_6) δ = 192.2, 172.5, 170.1, 166.4, 166.2, 162.8, 139.7, 137.9, 136.7, 131.8, 131.1, 121.6, 110.8, 99.6, 81.3, 63.3, 50.1, 40.1, 25.3, 18.8, 17.2, 14.4, 7.6 ppm. HRMS (ESI+): m/z = 446.1809 $[M+H]^+$, calc.: 446.1809. $[\alpha]_D^{20}$ = −920 (*c* = 0.92 in MeOH).

2.18.4 9-Isobutyl sorbicillactone A (35)

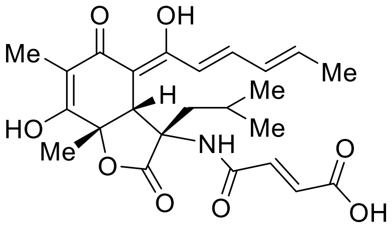

35

35 was synthesized and purified (t_R = 26.1 min) according to the general procedure 2.17, yielding 10 mg (23 µmol, 21%) of a yellow solid. mp: 133°C (dec.). ^{1}H-NMR (600 MHz, Acetone-d$_6$) δ = 7.60 (s, 1H), 7.17 (dd, *J* = 14.8, 11.0 Hz, 1H), 6.94 (d, *J* = 15.4 Hz, 1H), 6.53 (d, *J* = 15.4 Hz, 1H), 6.47 (d, *J* = 14.8 Hz, 1H), 6.35 – 6.27 (m, 1H), 6.21 – 6.12 (m, 1H), 3.71 (s, 1H), 1.84 (dd, *J* = 6.6, 1.9 Hz, 3H), 1.87 – 1.74 (m, 3H), 1.66 (s, 3H), 1.59 (s, 3H), 0.99 (d, *J* = 6.8 Hz, 3H), 0.94 (d, *J* = 6.6 Hz, 3H) ppm. ^{13}C-NMR (151 MHz, Acetone-d$_6$) δ = 192.2, 172.6, 170.3, 166.4, 166.0, 162.9, 139.7, 137.8, 136.8, 131.8, 131.1, 121.8, 110.8, 99.6, 81.3, 63.0, 51.3, 46.5, 25.5, 25.4, 24.5, 23.4, 18.8, 7.6 ppm. HRMS (ESI+): m/z = 460.1967 [M+H]$^+$, calc.: 460.1966. $[\alpha]_D^{20}$ = −795 (*c* = 1.23 in MeOH).

2.18.5 9-*sec* butyl sorbicillactone A (36)

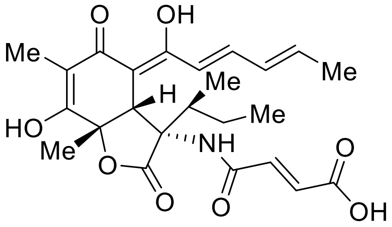

36

36 was synthesized and purified (t_R = 26.0 min) according to the general procedure 2.17, yielding 9 mg (20 µmol, 19%) of a yellow solid. mp: 155°C (dec.). ^{1}H-NMR (600 MHz, Acetone-d$_6$) δ = 7.70 (s, 1H), 7.15 (dd, *J* = 14.7, 11.0 Hz, 1H), 6.95 (d, *J* = 15.4 Hz, 1H), 6.54 (d, *J* = 15.4 Hz, 1H), 6.33 (d, J = 14.9 Hz, 1H), 6.34 – 6.28 (m, 1H), 6.19 – 6.11 (m, 1H), 3.83 (s, 1H), 1.84 (dd, *J* = 6.9, 1.7 Hz, 3H), 1.83 – 1.78 (m, 1H), 1.77 – 1.71 (m, 1H), 1.66 (s, 3H), 1.63 – 1.60 (m, 1H), 1.59 (s, 3H), 1.20 (d, *J* = 6.7 Hz, 3H), 0.86 (t, *J* = 7.4 Hz, 3H) ppm. ^{13}C-NMR (151 MHz, Acetone-d$_6$) δ = 192.5, 171.1, 170.0, 166.4, 166.2, 163.1, 139.7, 137.8, 136.8, 131.9, 131.1, 121.5, 110.8, 99.8, 81.3, 66.7, 49.2, 40.5, 25.3, 23.4, 18.7, 13.7, 13.1, 7.6 ppm. HRMS (ESI+): m/z = 460.1964 [M+H]$^+$, calc.: 460.1966. $[\alpha]_D^{20}$ = −772 (*c* = 0.51 in MeOH).

2.19 Formation of azlactone during activation of carboxylic acid of (24)

Experiments on the activation of **24** with pentafluorophenol for the conversion with sorbicillinol (**2**), similar to the synthesis of sorbicillactone derivative (5S,6R,9S)-**16a**, did not result in any product formation.

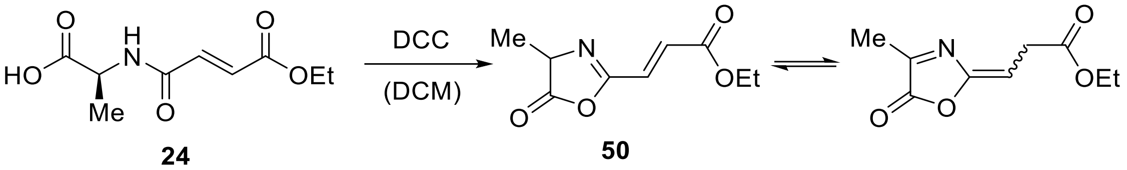

Therefore, a test reaction with **24** (160 mg, 0.73 mmol, 1.0 eq.) and DCC (169 mg, 0.82 mmol, 1.05 eq.) was carried out in DCM (3 mL) at rt for 2 h without the addition of pentafluorophenol. After filtration of the reaction mixture and evaporation of the solvent, NMR analysis (figure S1) and HRMS analysis (figure S2) was performed which finally revealed the formation of an azlactone.

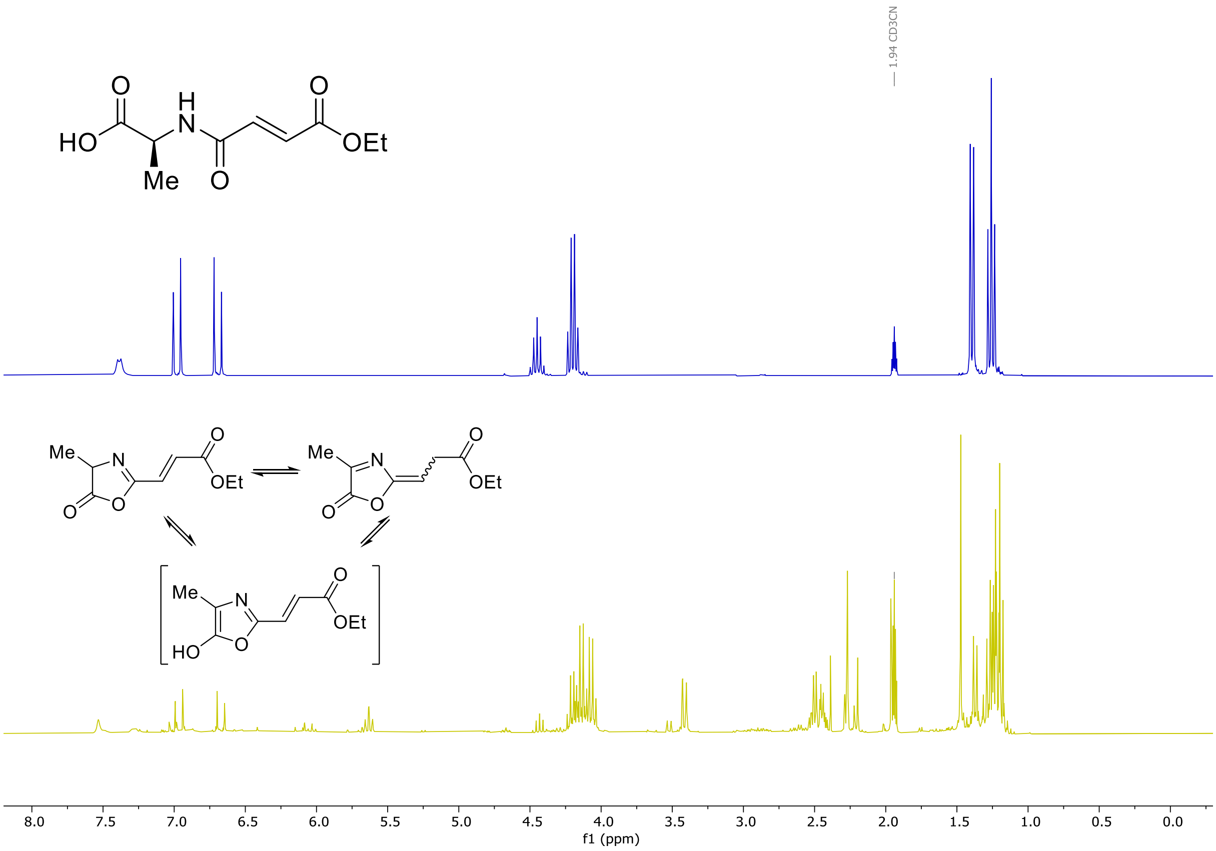

Fig. S1 Comparison of the NMR spectra of **24** (blue) and azlactone **50** (yellow) in MeCN-d_3.

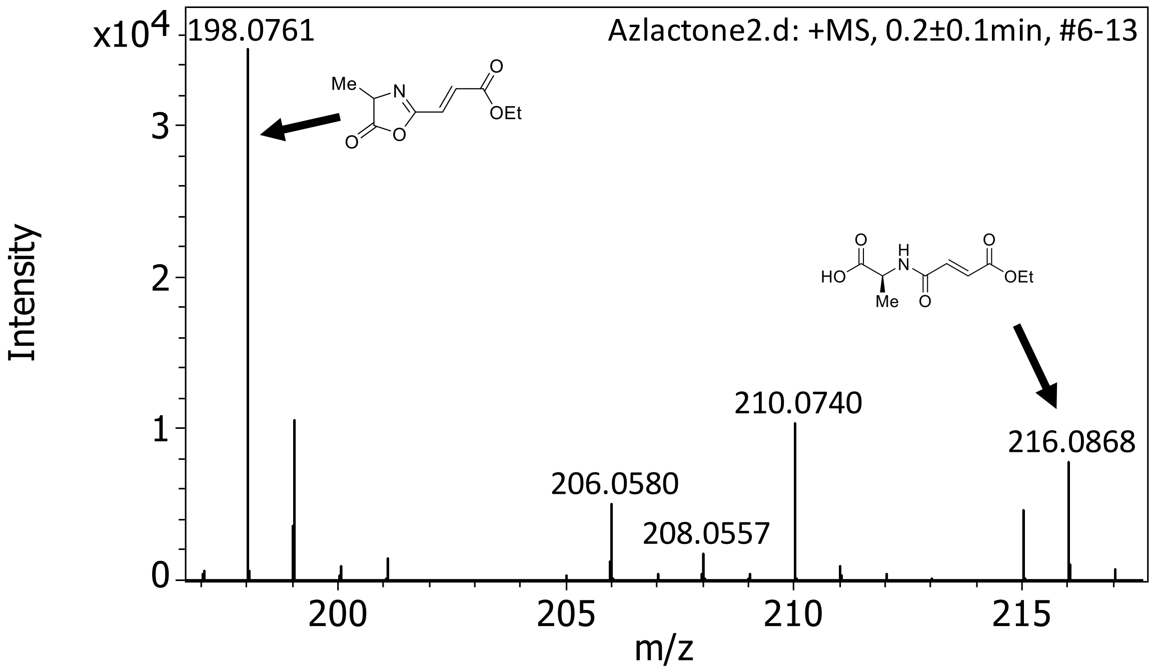

Fig. S2 HRMS data of azlactone **50** (calc.: HRMS (ESI+): m/z = 198.0761 $[M+H]^+$) and **24** (calc.: HRMS (ESI+): m/z = 216.0866 $[M+H]^+$).

3. Total Synthesis of Sorbicillactone A by Harned et al.

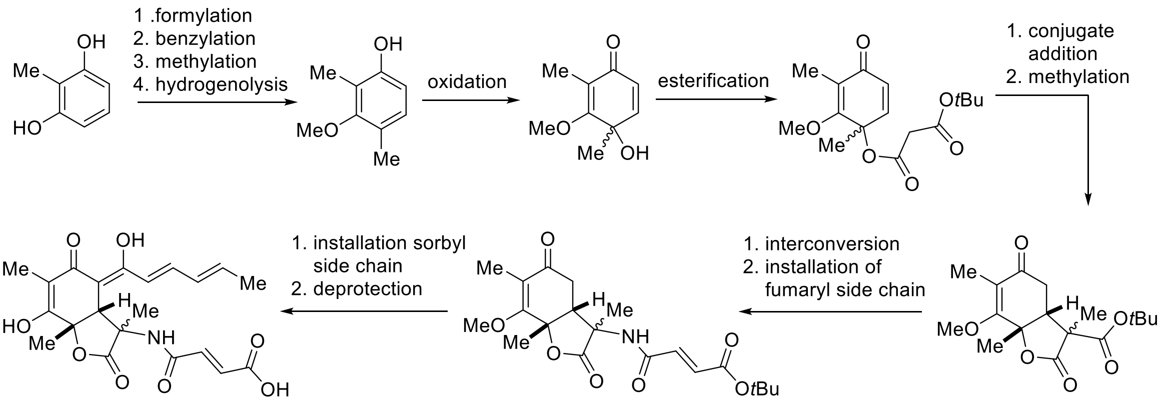

Fig. S3 Non-stereoselective total synthesis of sorbicillactone A published by Harned et al.[5]

4. Supplementary References

1. Sib, A. & Gulder, T. A. M. Stereoselective Total Synthesis of Bisorbicillinoid Natural Products by Enzymatic Oxidative Dearomatization/Dimerization. *Angew. Chem. Int. Ed.* **56,** 12888–12891 (2017).
2. Milzarek, T. M., Schuler, S., Matura, A. & Gulder, T. A. M. Evaluation of the Substrate Promiscuity of SorbC for the Chemo-Enzymatic Total Synthesis of Structurally Diverse Sorbicillinoids. *ACS Catal.* **12,** 1898–1904 (2022).
3. Wang, K., Lu, Y. & Ishihara, K. The ortho-substituent on 2,4-bis(trifluoromethyl)phenylboronic acid catalyzed dehydrative condensation between carboxylic acids and amines. *Chem. Commun.* **54,** 5410–5413 (2018).
4. Bringmann, G. *et al.* The first sorbicillinoid alkaloids, the antileukemic sorbicillactones A and B, from a sponge-derived Penicillium chrysogenum strain. *Tetrahedron* **61,** 7252–7265 (2005).
5. Volp, K. A., Johnson, D. M. & Harned, A. M. A concise synthetic approach to the sorbicillactones: total synthesis of sorbicillactone A and 9-epi-sorbicillactone A. *Org. Lett.* **13,** 4486–4489 (2011).
