# Supplementary material for: Chemoenzymatic total synthesis of sorbicillactone A
Source: Commun Chem. 2024 Feb 24;7:39. doi: 10.1038/s42004-024-01126-1 (PMC10894215; doi:10.1038/s42004-024-01126-1)
Supplement: Supplementary file 3 — Description of Additional Supplementary Files [file 42004_2024_1126_MOESM3_ESM.pdf]

Description of Additional Supplementary Files

File name: Supplementary Data 1

Description: NMR spectra
