# Supplementary material for: Chemoenzymatic total synthesis of sorbicillactone A
Source: Commun Chem. 2024 Feb 24;7:39. doi: 10.1038/s42004-024-01126-1 (PMC10894215; doi:10.1038/s42004-024-01126-1)
Supplement: Supplementary file 4 — Supplementary Data 1 [file 42004_2024_1126_MOESM4_ESM.pdf]

Chemoenzymatic Total Synthesis of Sorbicillactone A

Jonas I. Müller[1], Tobias A. M. Gulder[1,2]

1 Chair of Technical Biochemistry, Technical University of Dresden, Bergstraße 66, 01069 Dresden, Germany,
E-mail: tobias.gulder@tu-dresden.de
2 Helmholtz Institute for Pharmaceutical Research Saarland (HIPS), Department of Natural Product Biotech-nology, Helmholtz Centre for Infection Research (HZI) and Department of Pharmacy at Saarland University, 66123 Saarbrücken, Germany.

NMR Data

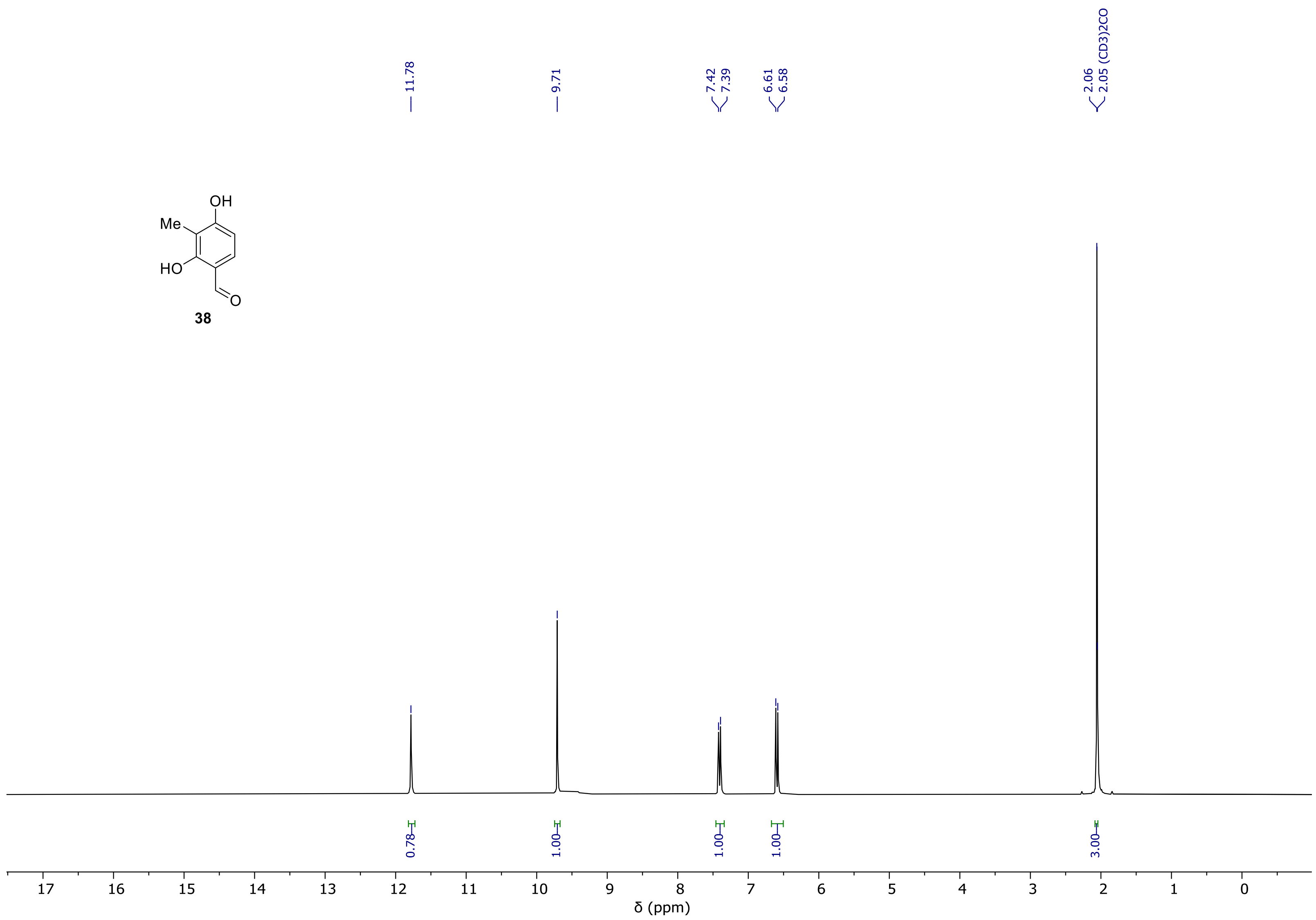

Fig. S4 ^{1}H-NMR spectrum of 2,4-dihydroxy-3-methylbenzaldehyde (**38**), measured in acetone-d_6 at 300 MHz.

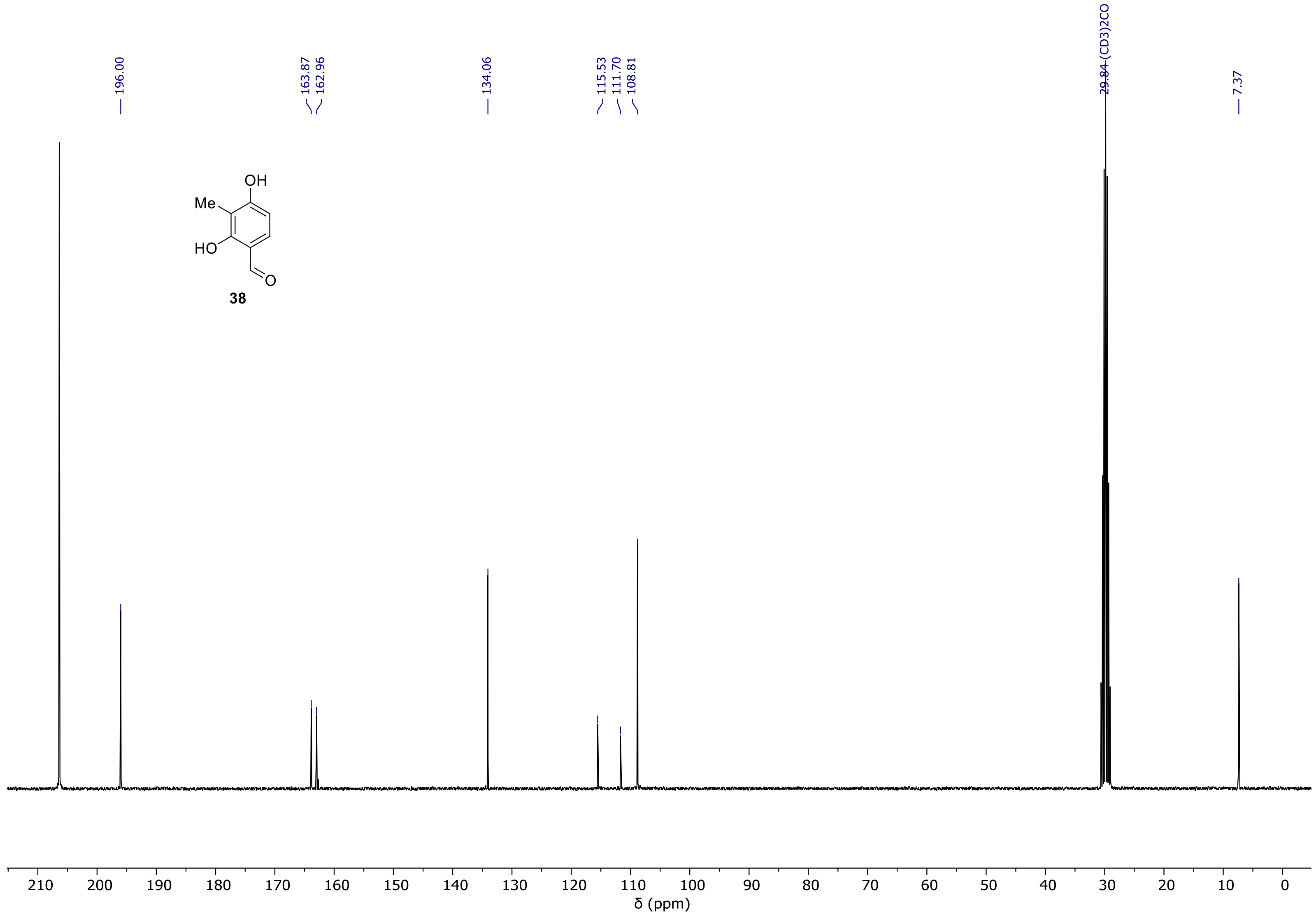

Fig. S5 ^{13}C-NMR spectrum of 2,4-dihydroxy-3-methylbenzaldehyde (**38**), measured in acetone-d_6 at 75 MHz.

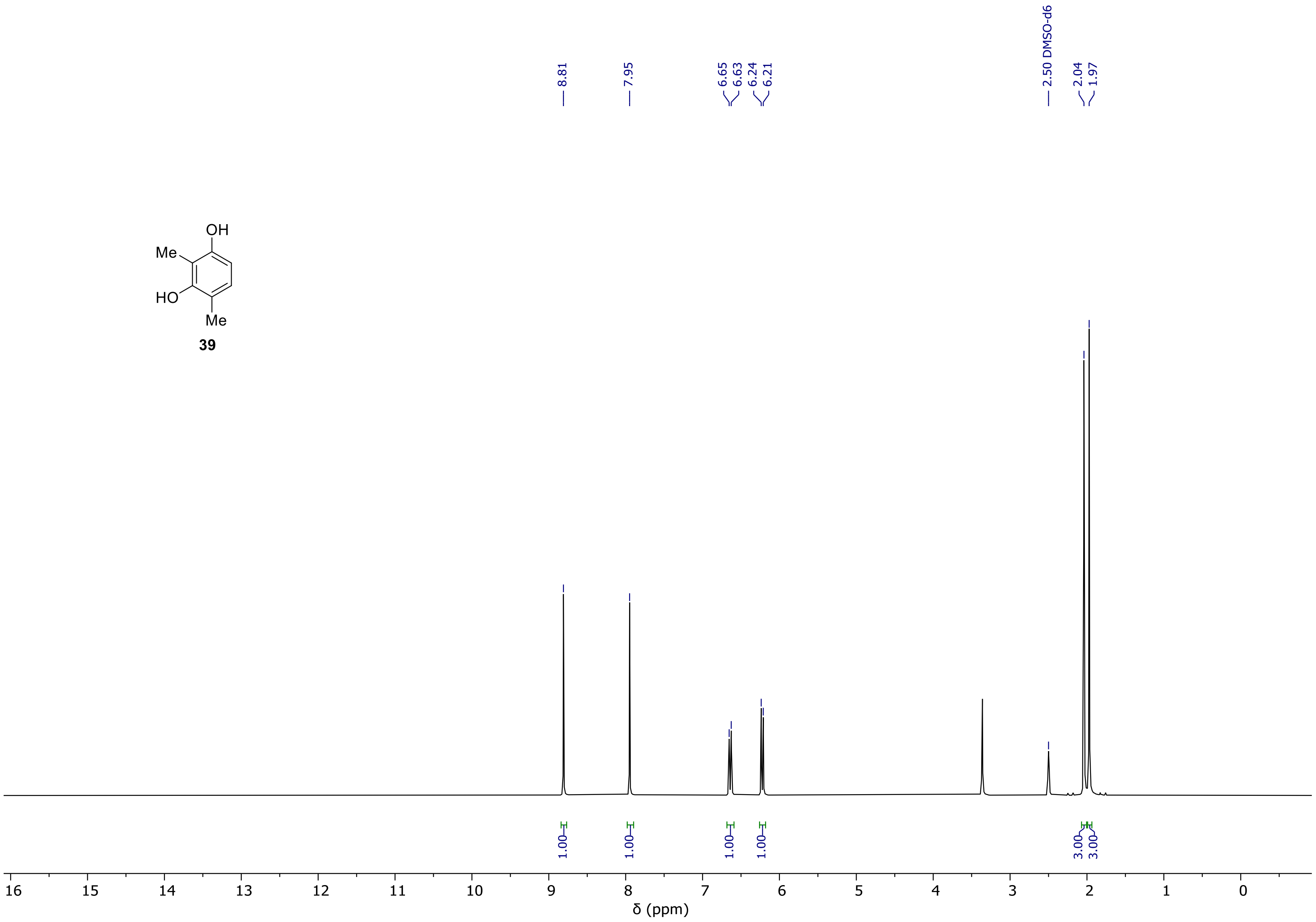

Fig. S6 ^{1}H-NMR spectrum of 2,4-dimethylresorcinol (**39**), measured in DMSO-d_6 at 300 MHz.

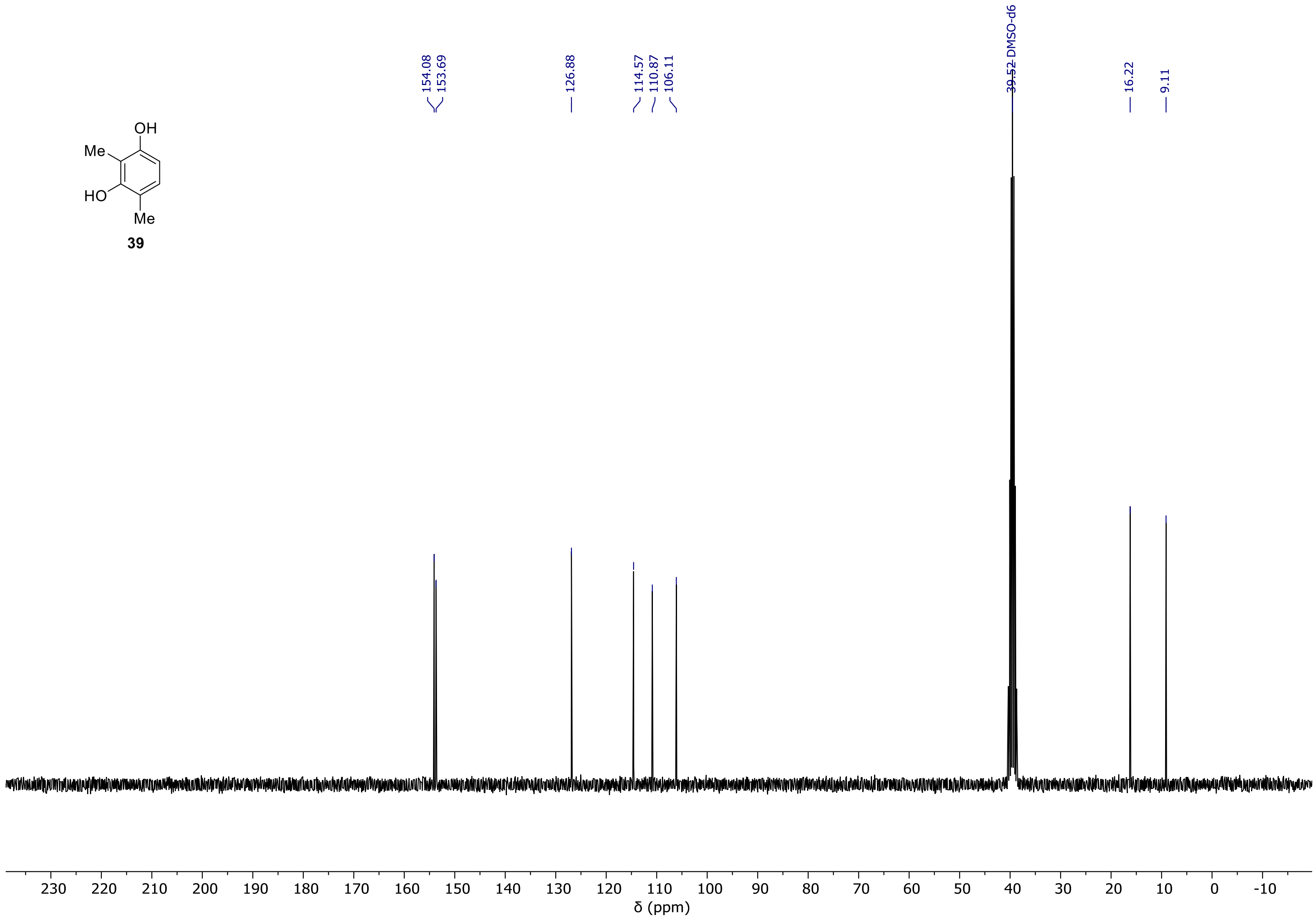

Fig. S7 ^{13}C-NMR spectrum of 2,4-dimethylresorcinol (**39**), measured in DMSO-d_6 at 75 MHz.

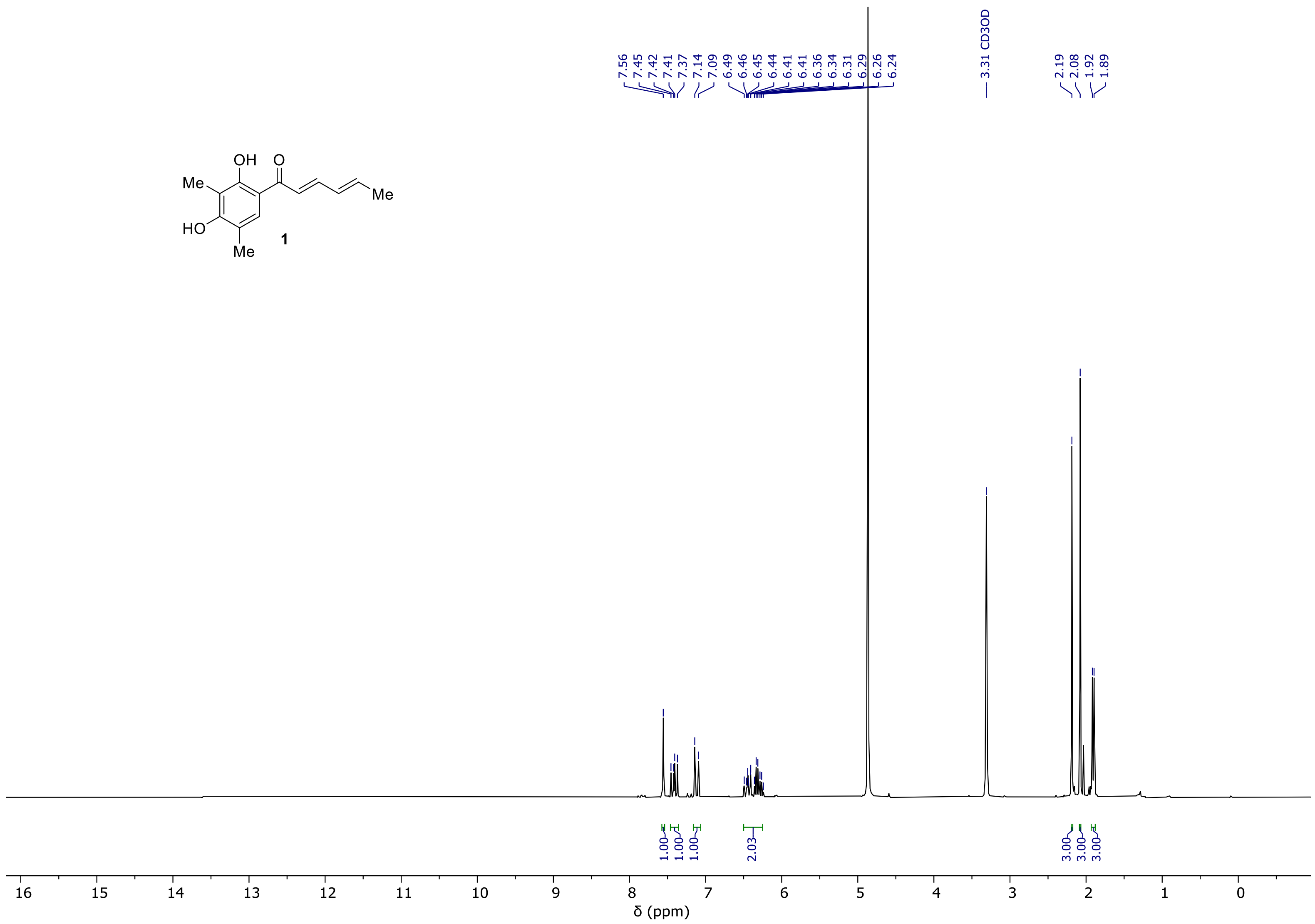

Fig. S8 ^{1}H-NMR spectrum of sorbicillin (**1**), measured in methanol-d_6 at 300 MHz.

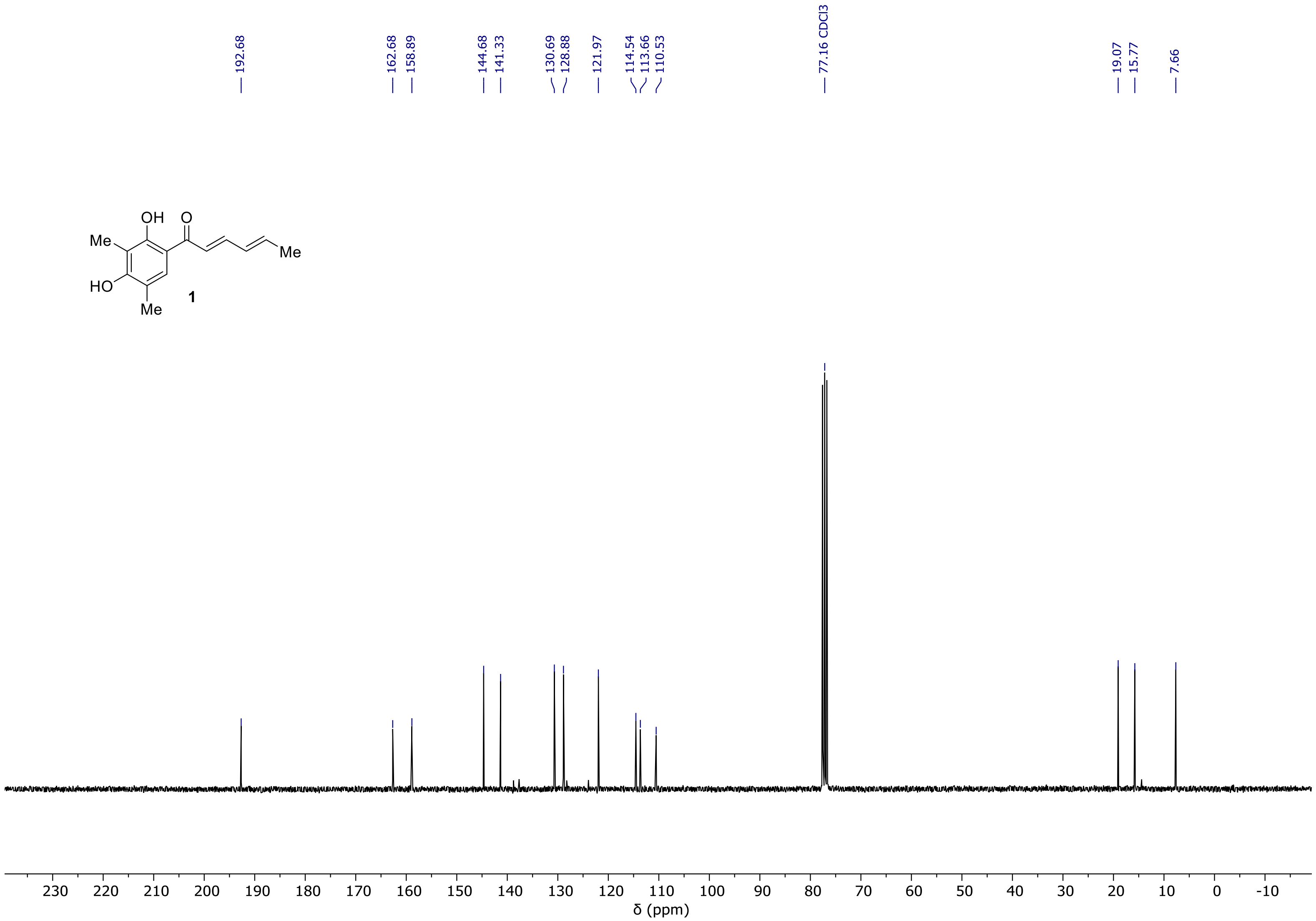

Fig. S9 ^{13}C-NMR spectrum of sorbicillin (**1**), measured in $CDCl_3$ at 75 MHz.

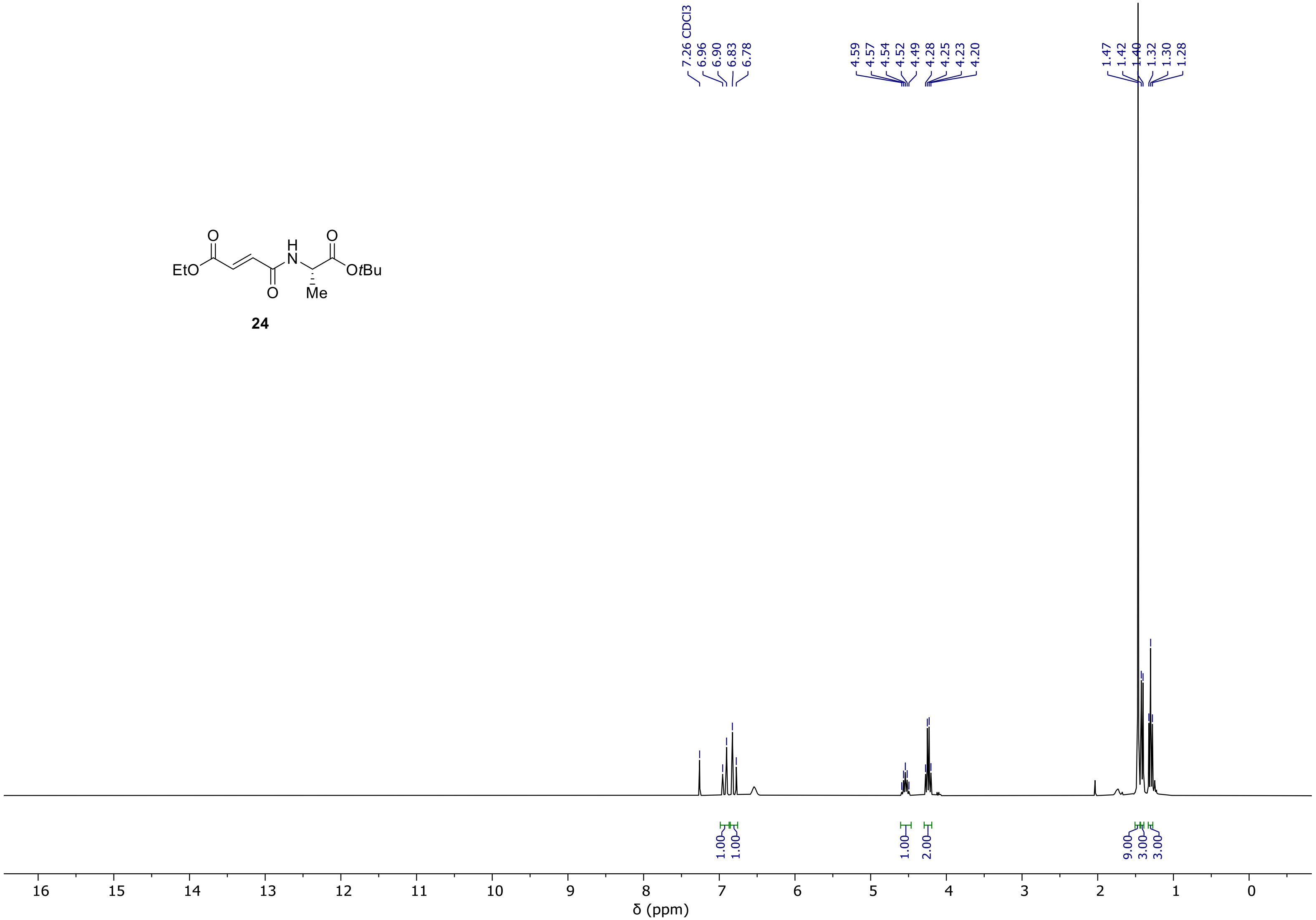

Fig. S10 ^{1}H-NMR spectrum of ethyl fumarate-*L*-Ala-O*t*Bu (**24**), measured in $CDCl_3$ at 300 MHz.

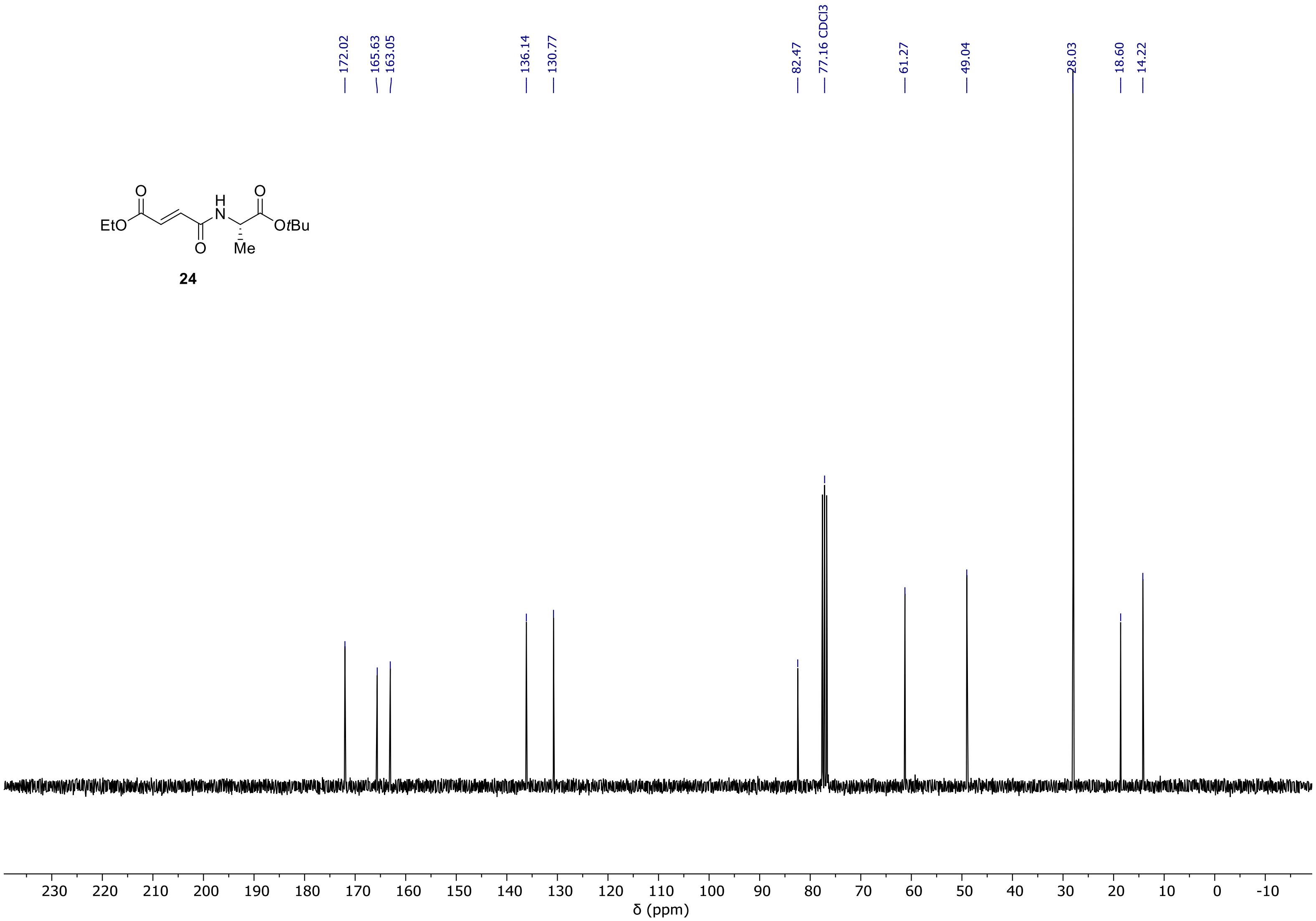

Fig. S11 ^{13}C-NMR spectrum of ethyl fumarate-*L*-Ala-O*t*Bu (**24**), measured in $CDCl_3$ at 75 MHz.

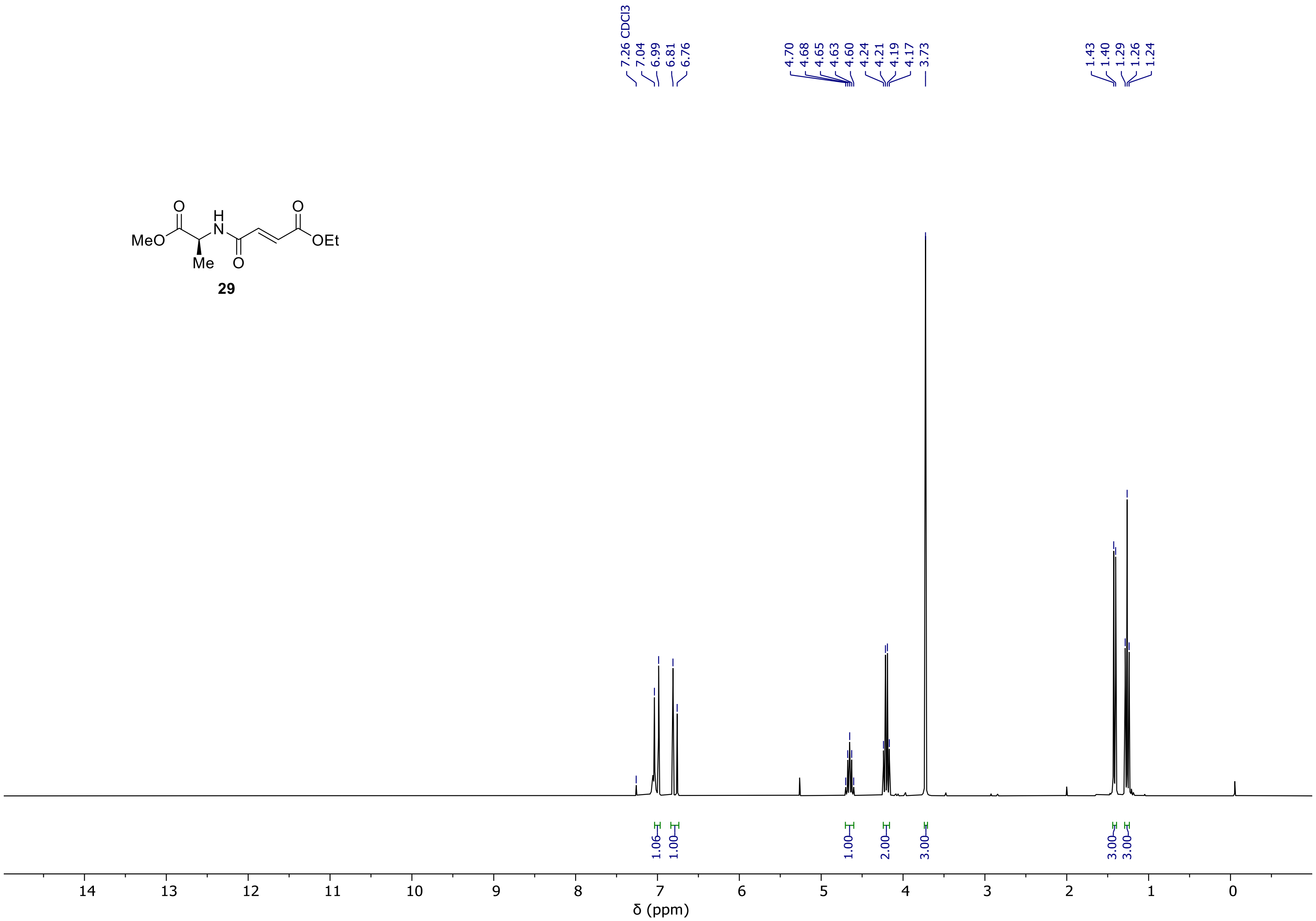

Fig. S12 ^{1}H-NMR spectrum of ethyl fumarate-*L*-Ala-OMe (**29**), measured in $CDCl_3$ at 300 MHz.

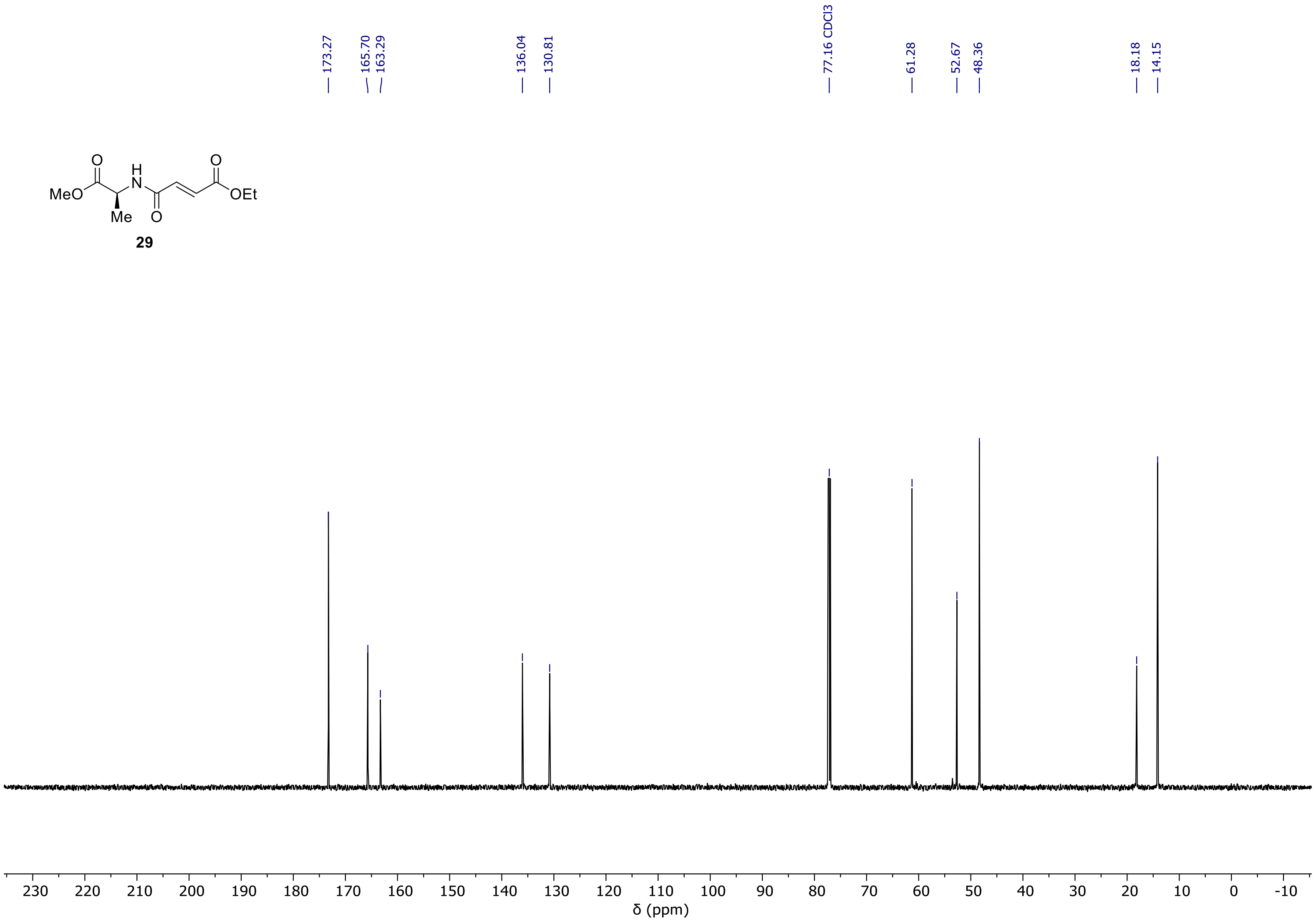

Fig. S13 ^{13}C-NMR spectrum of ethyl fumarate-*L*-Ala-OMe (**29**), measured in $CDCl_3$ at 75 MHz.

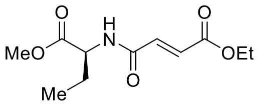

41

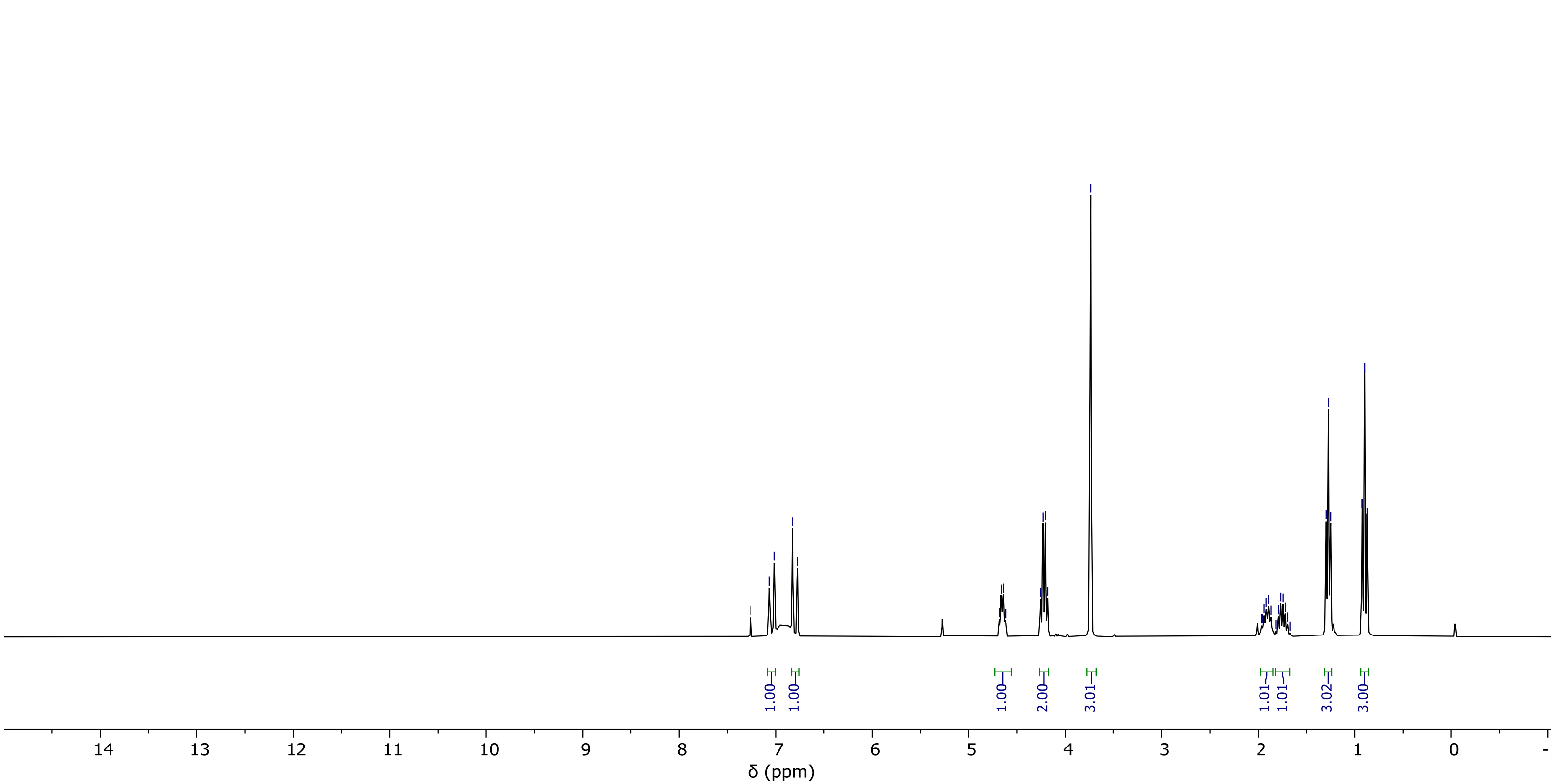

Fig. S14 ^{1}H-NMR spectrum of ethyl fumarate-*L*-Abu-OMe (**41**), measured in $CDCl_3$ at 300 MHz.

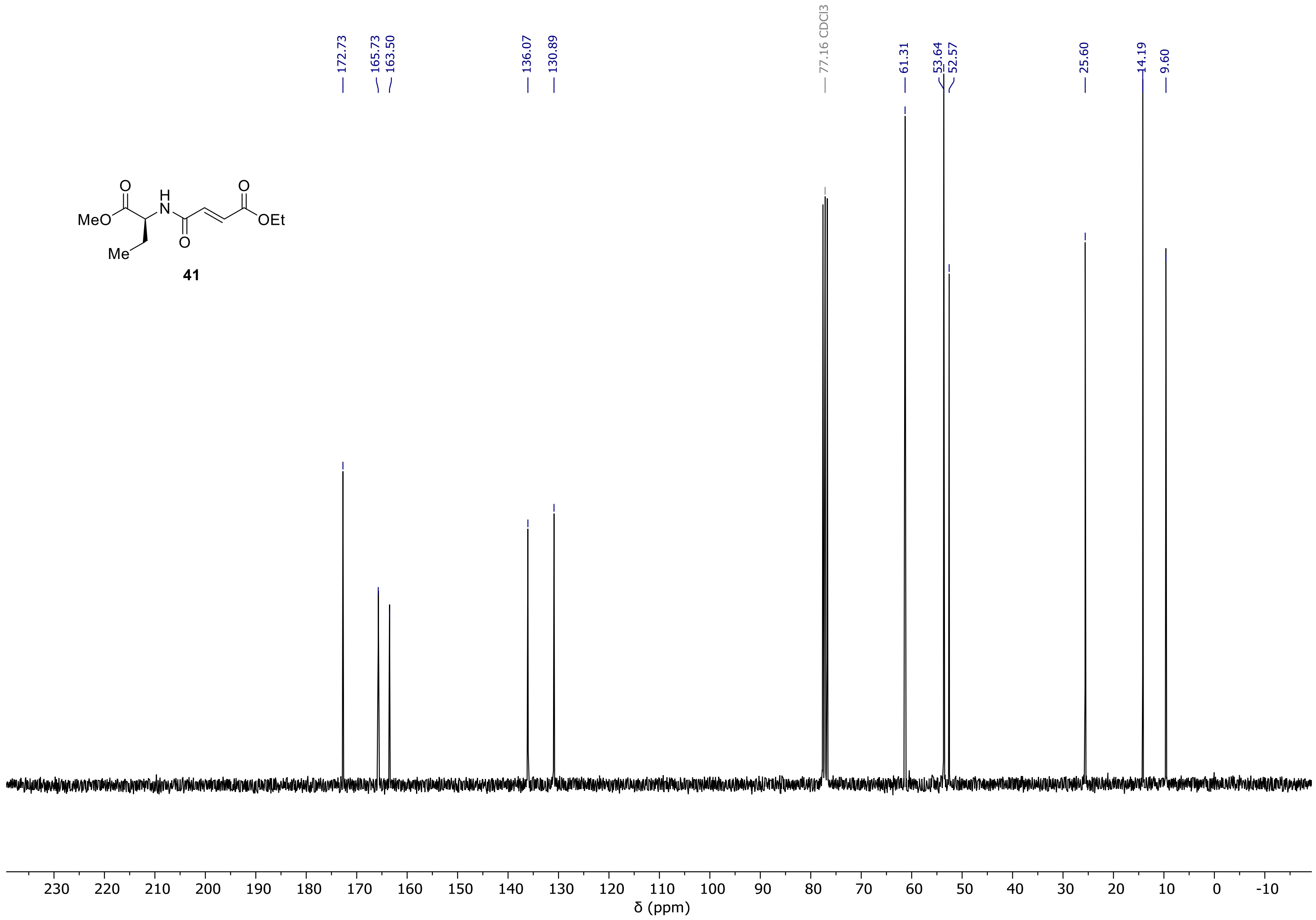

Fig. S15 ^{13}C-NMR spectrum of ethyl fumarate-*L*-Abu-OMe (**41**), measured in $CDCl_3$ at 75 MHz.

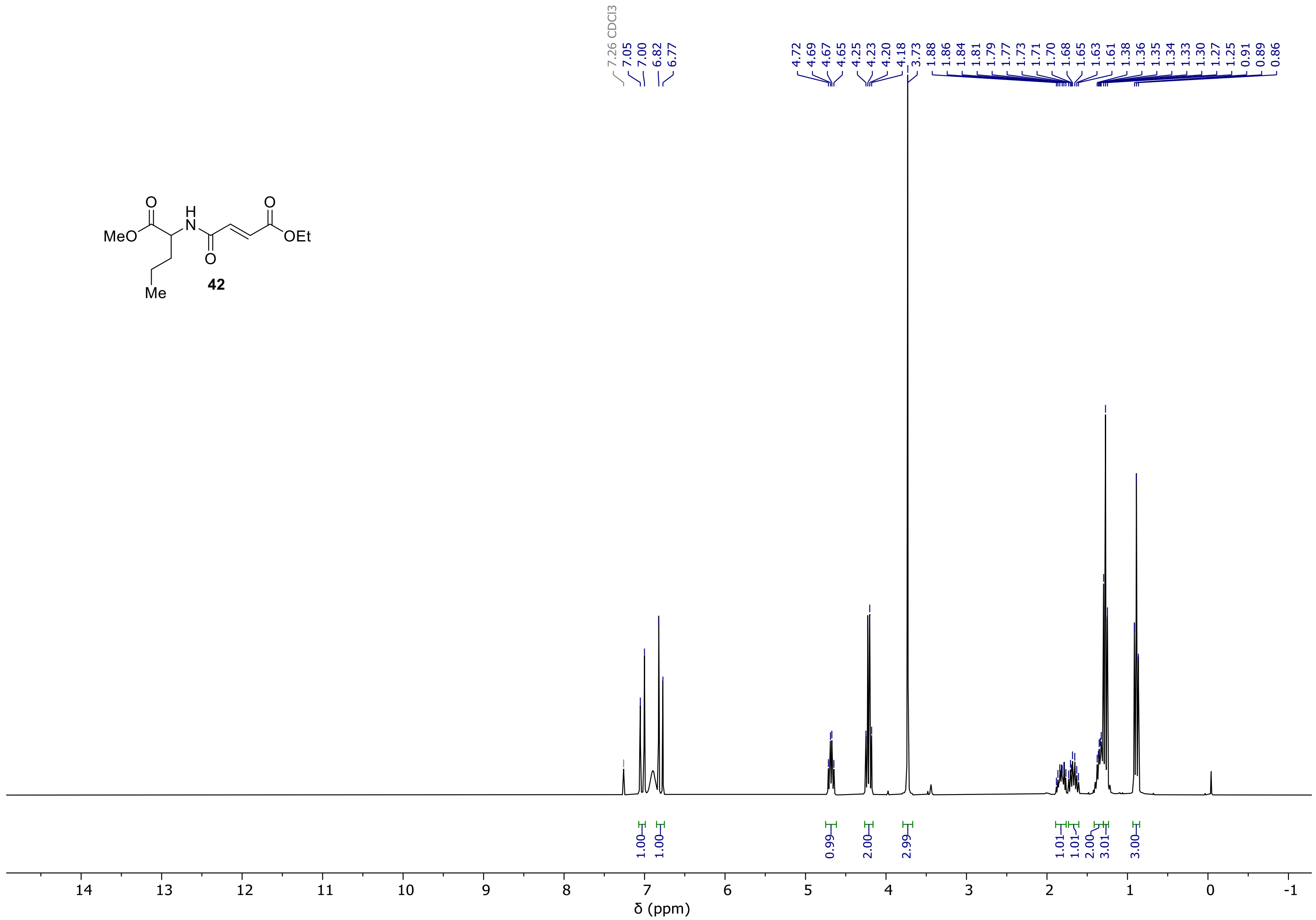

Fig. S16 ^{1}H-NMR spectrum of ethyl fumarate-Nva-OMe (**42**), measured in $CDCl_3$ at 300 MHz.

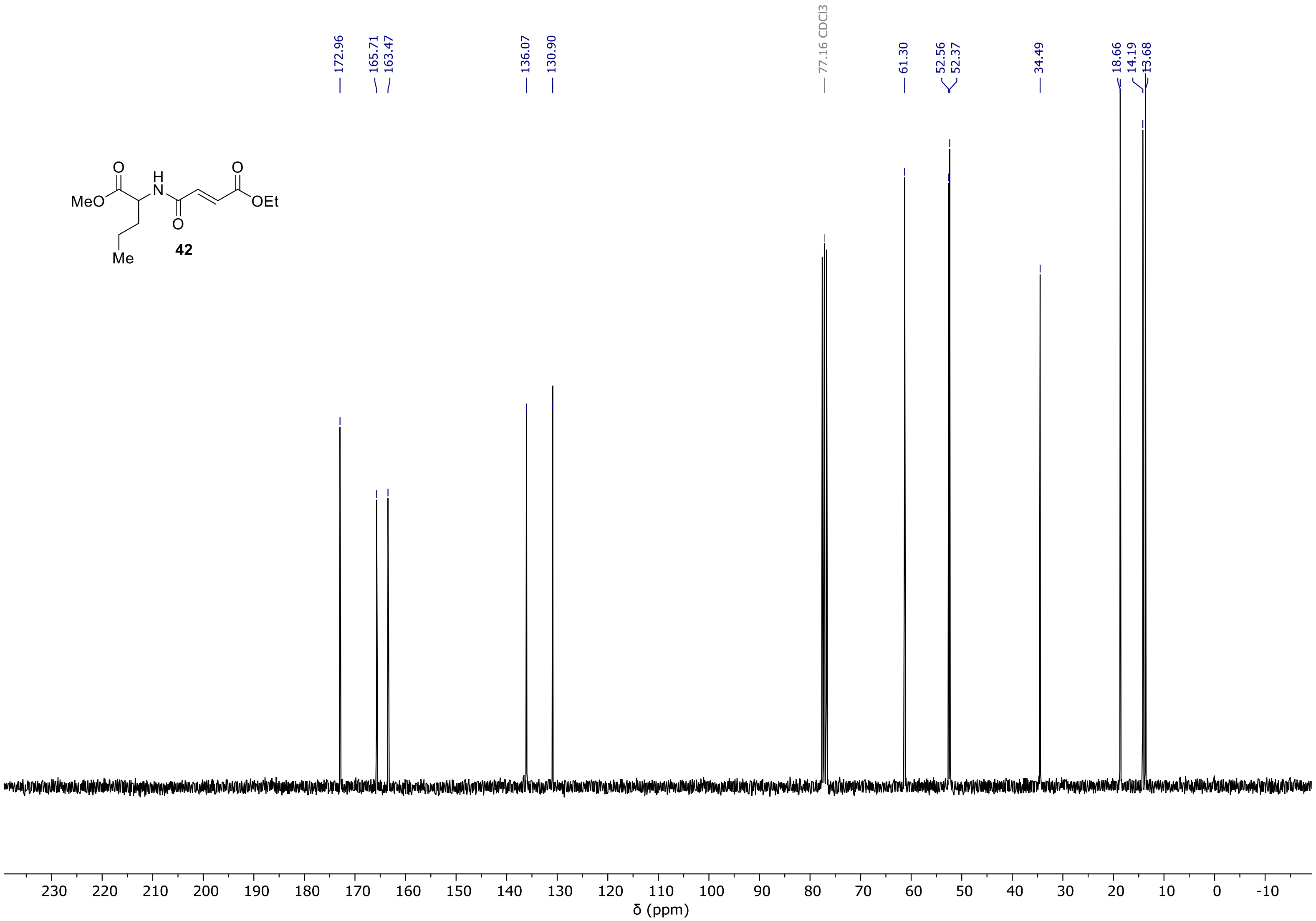

Fig. S17 ^{13}C-NMR spectrum of ethyl fumarate-Nva-OMe (**42**), measured in $CDCl_3$ at 75 MHz.

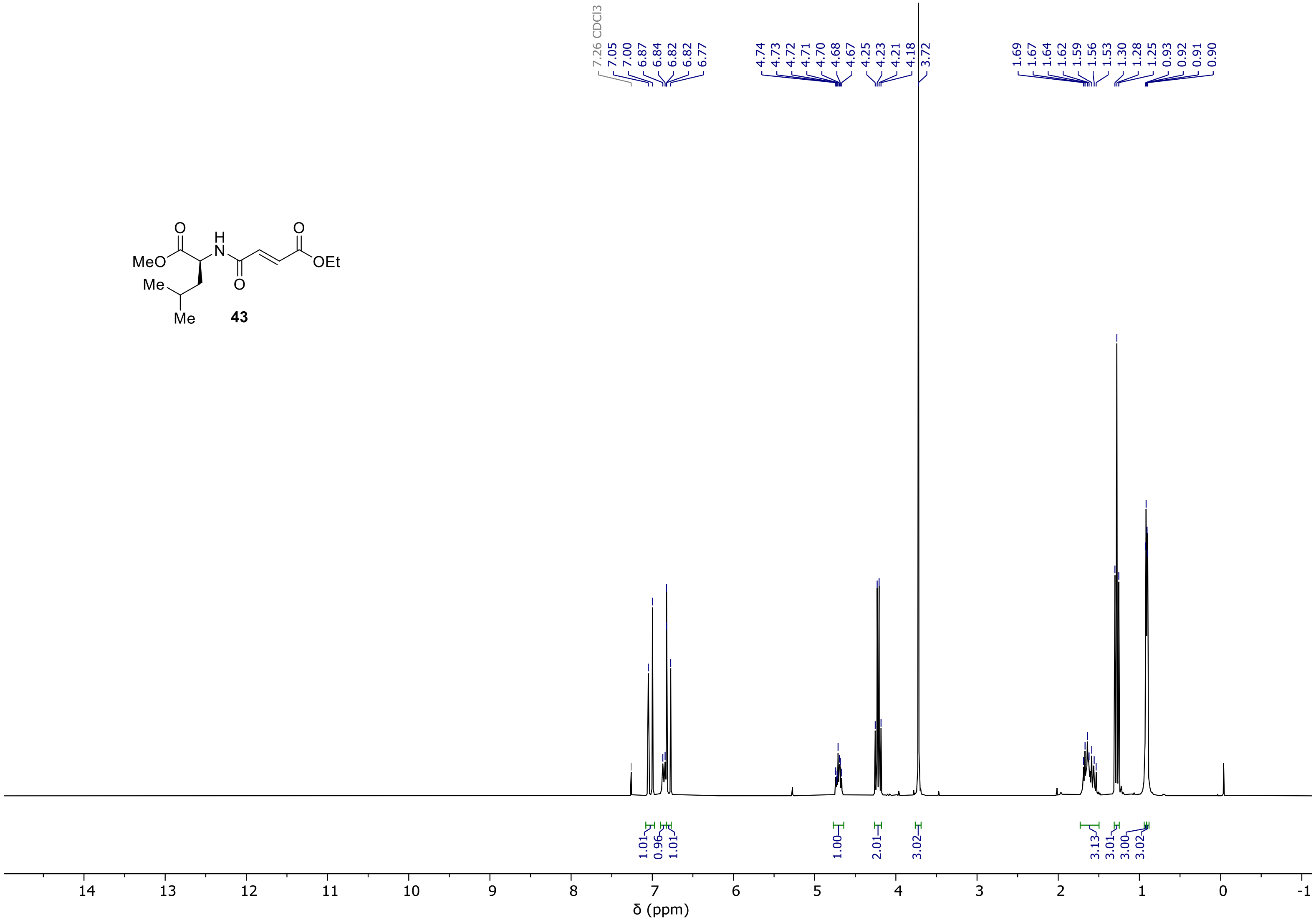

Fig. S18 ^{1}H-NMR spectrum of ethyl fumarate-*L*-Leu-OMe (**43**), measured in $CDCl_3$ at 300 MHz.

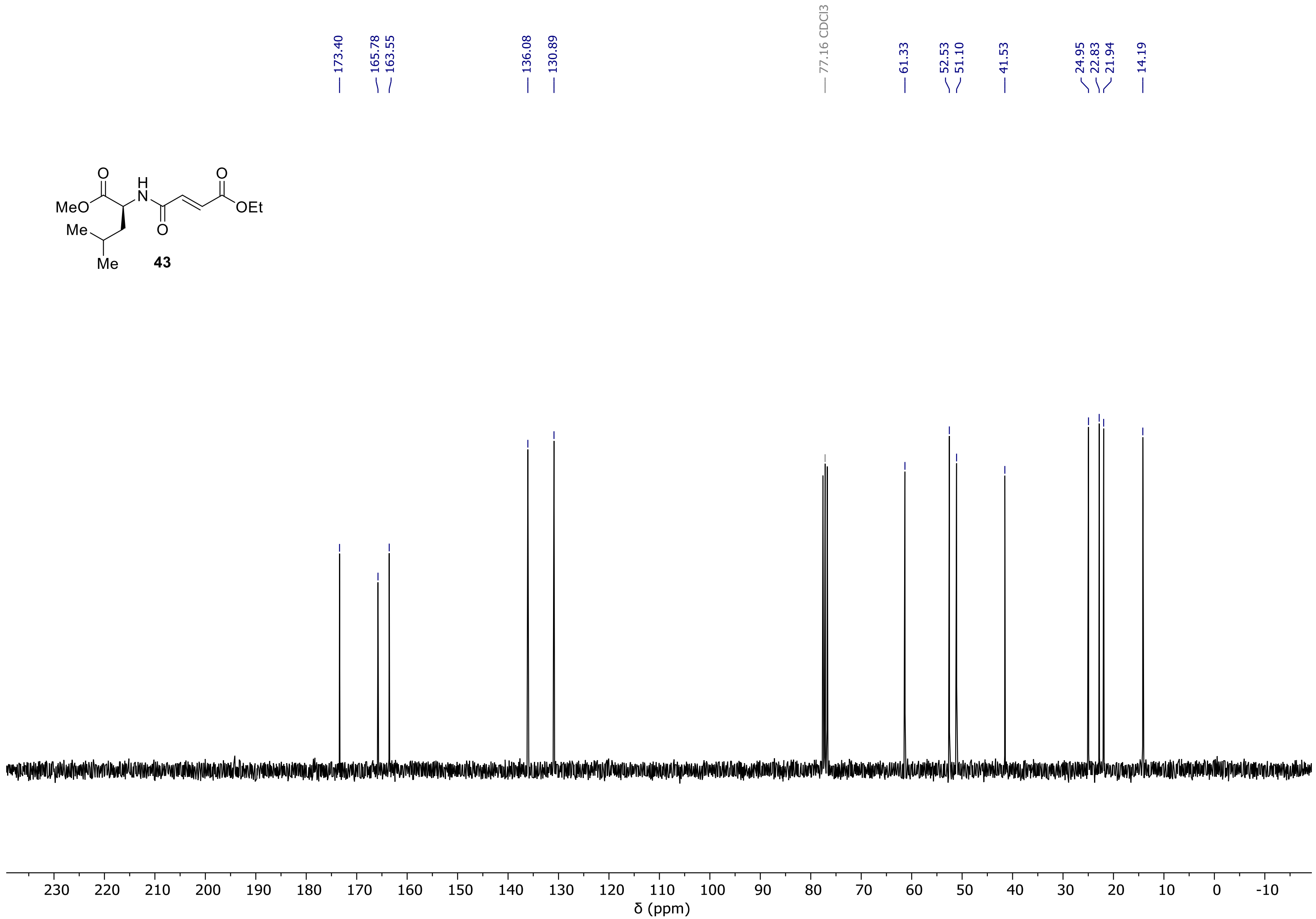

43

Fig. S19 ^{13}C-NMR spectrum of ethyl fumarate-*L*-Leu-OMe (**43**), measured in $CDCl_3$ at 75 MHz.

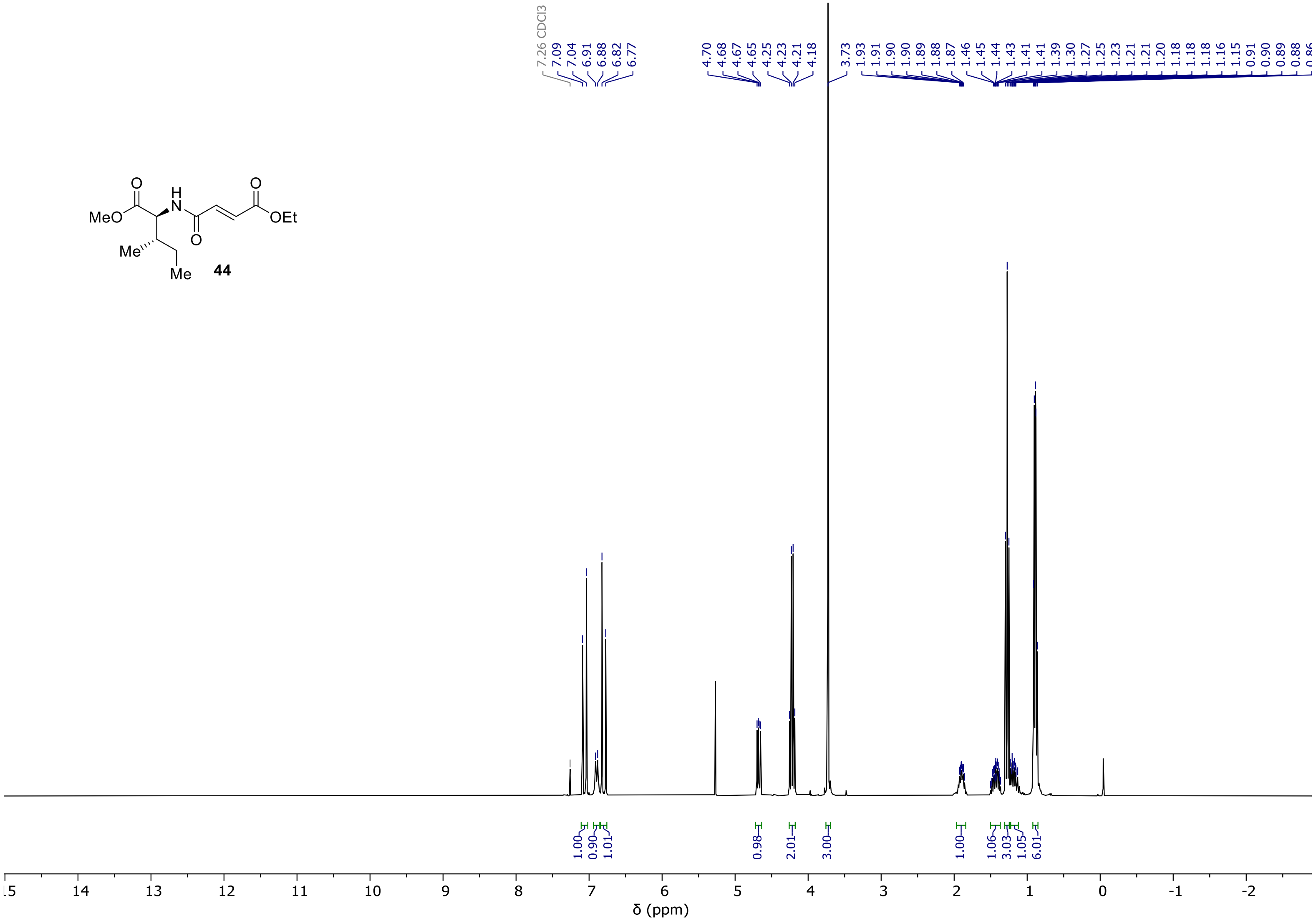

Fig. S20 ^{1}H-NMR spectrum of ethyl fumarate-*L*-Ile-OMe (**44**), measured in $CDCl_3$ at 300 MHz.

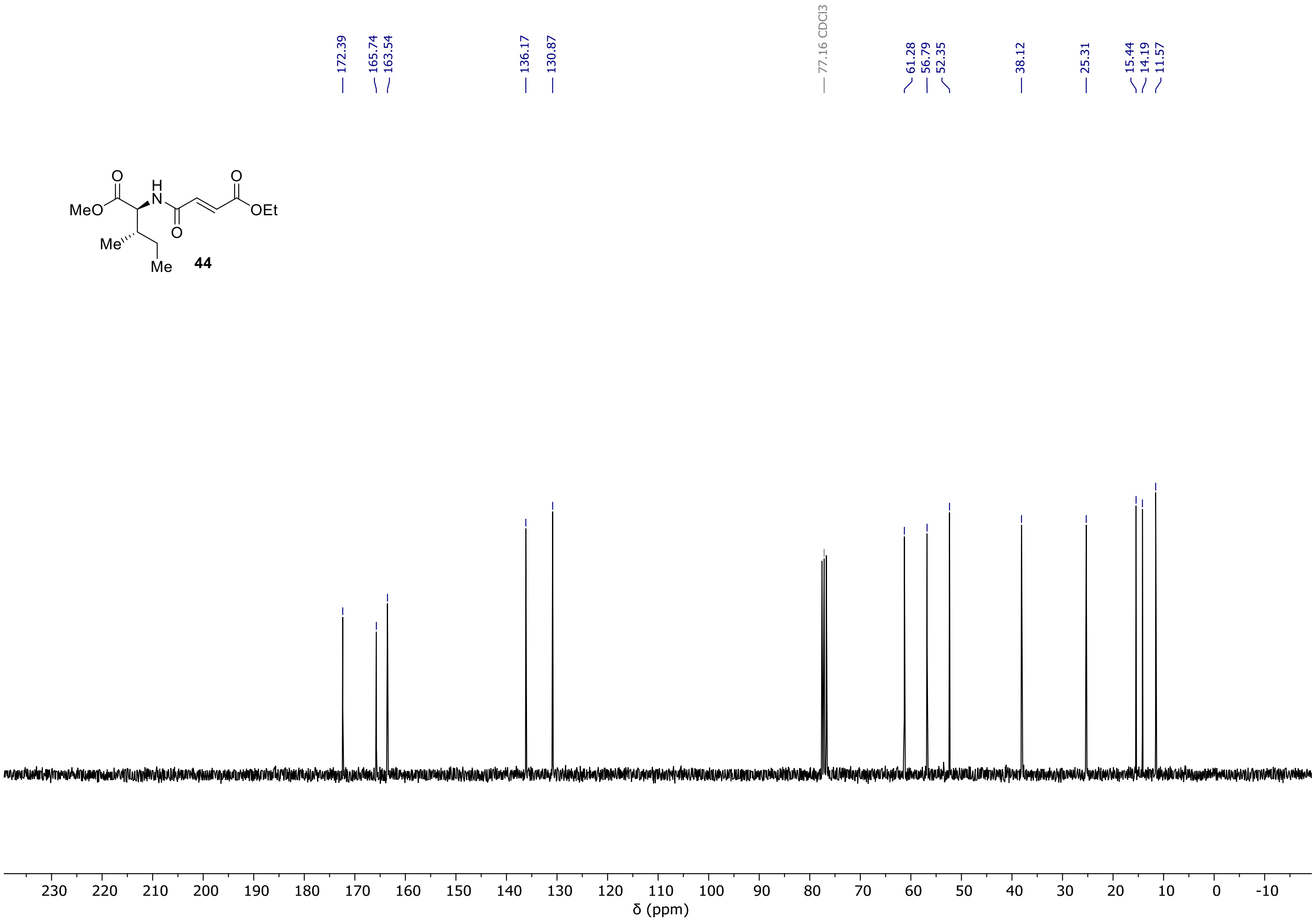

Fig. S21 ^{13}C-NMR spectrum of ethyl fumarate-*L*-Ile-OMe (**44**), measured in $CDCl_3$ at 75 MHz.

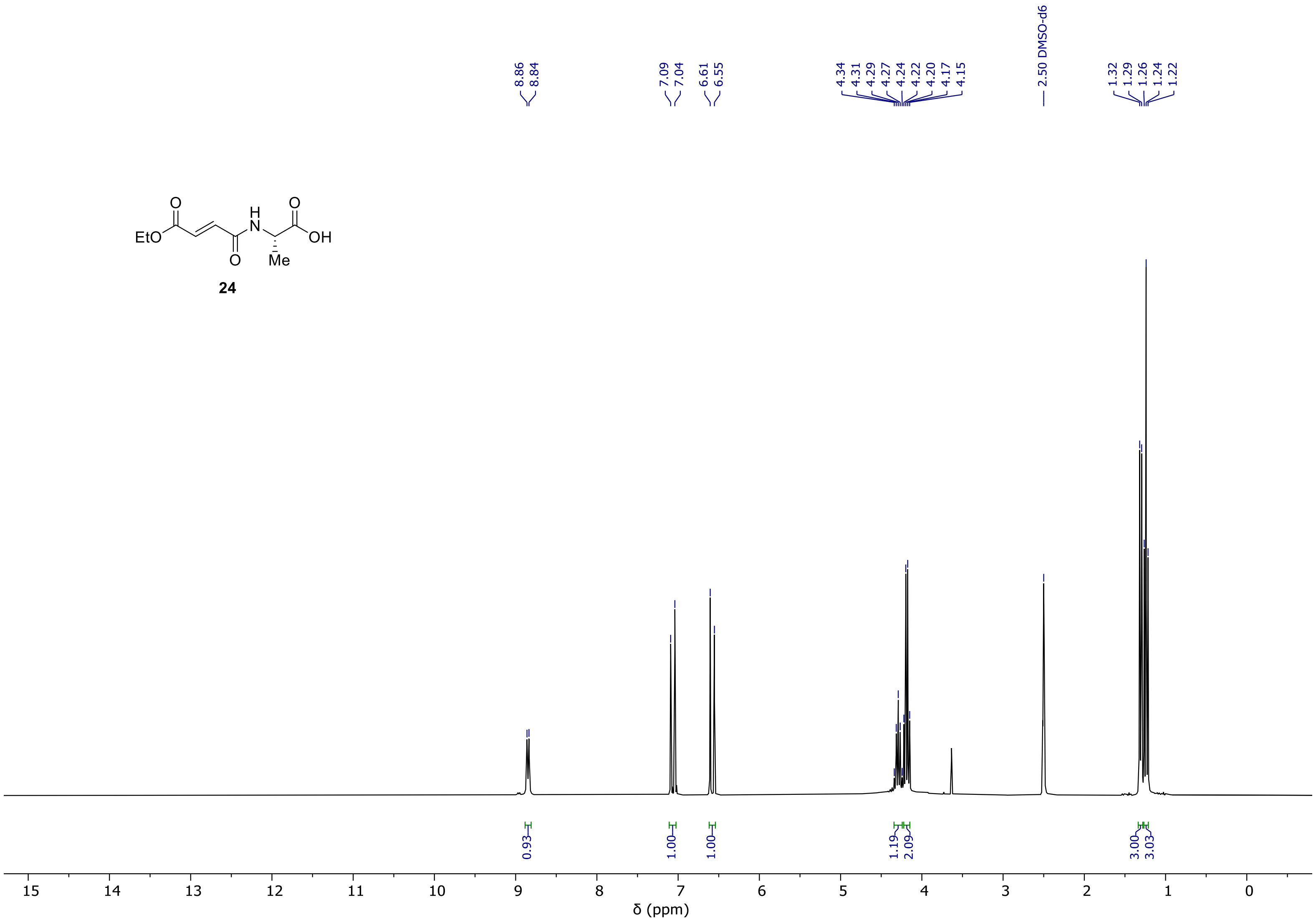

Fig. S22 ^{1}H-NMR spectrum of ethyl fumarate-*L*-Ala-OH (**24**), measured in DMSO-d_6 at 300 MHz.

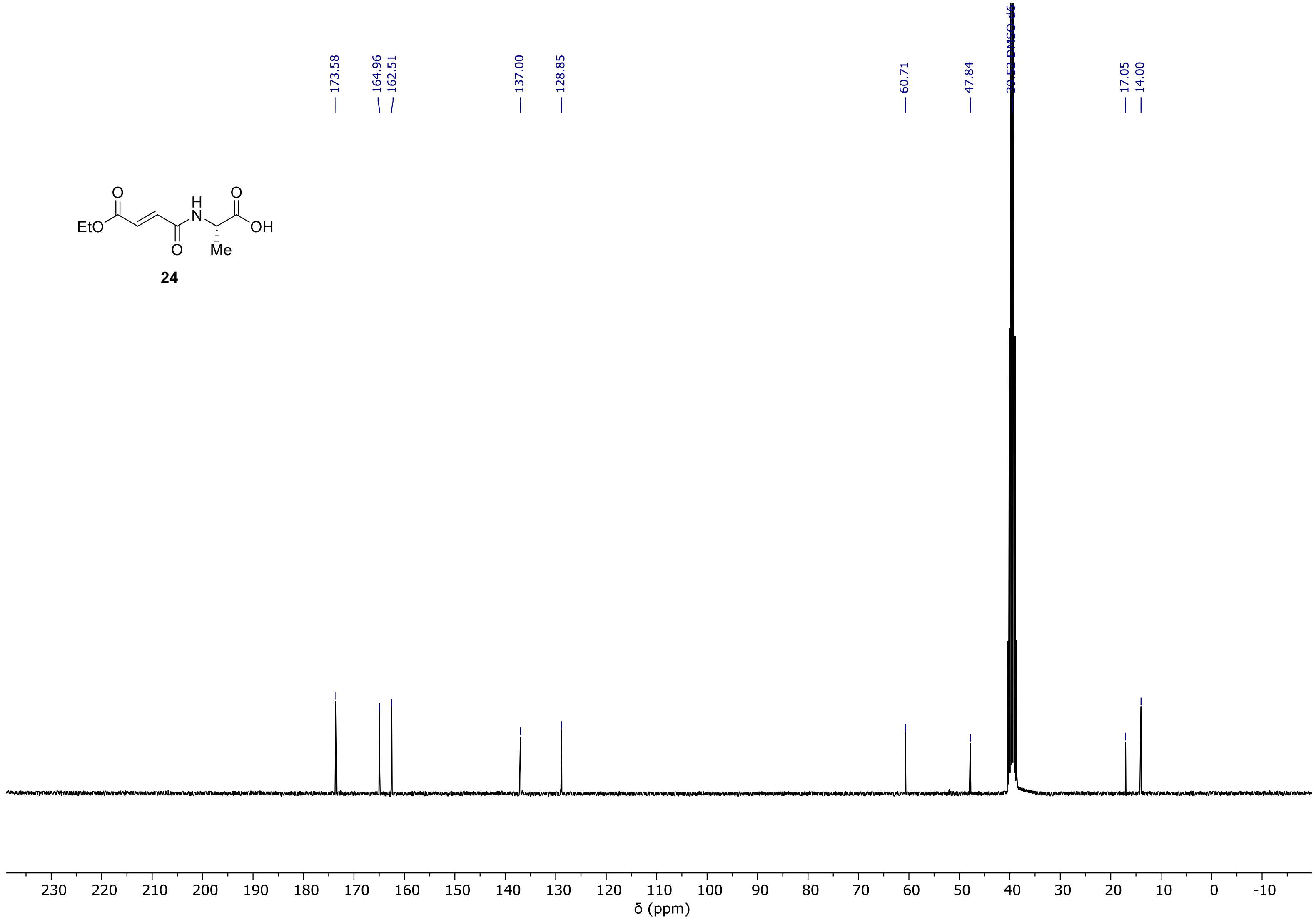

Fig. S23 ^{13}C-NMR spectrum of ethyl fumarate-*L*-Ala-OH (**24**), measured in DMSO-d$_6$ at 75 MHz.

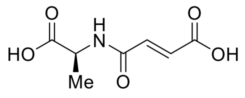

30

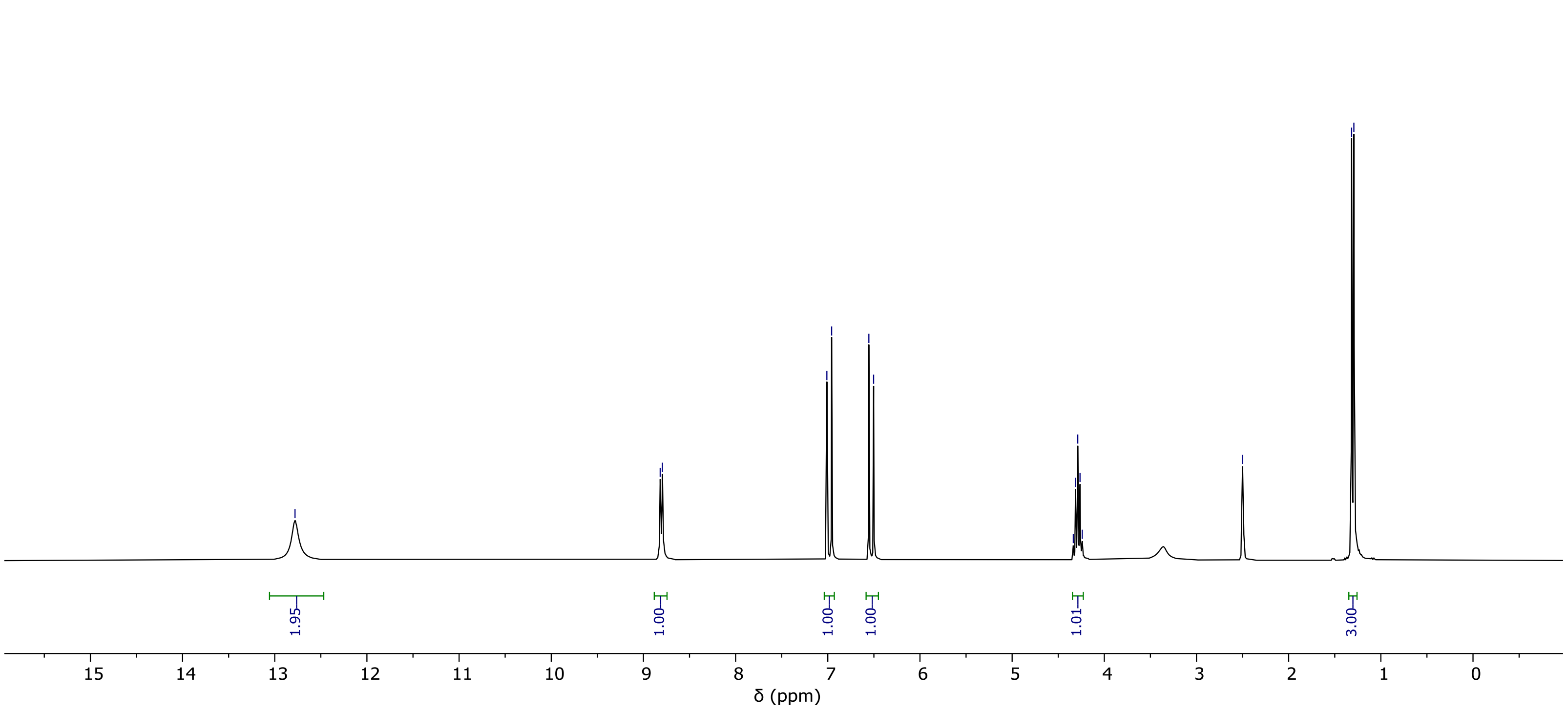

Fig. S24 ^{1}H-NMR spectrum of *L*-Ala-fumaric acid (**30**), measured in DMSO-d$_6$ at 300 MHz.

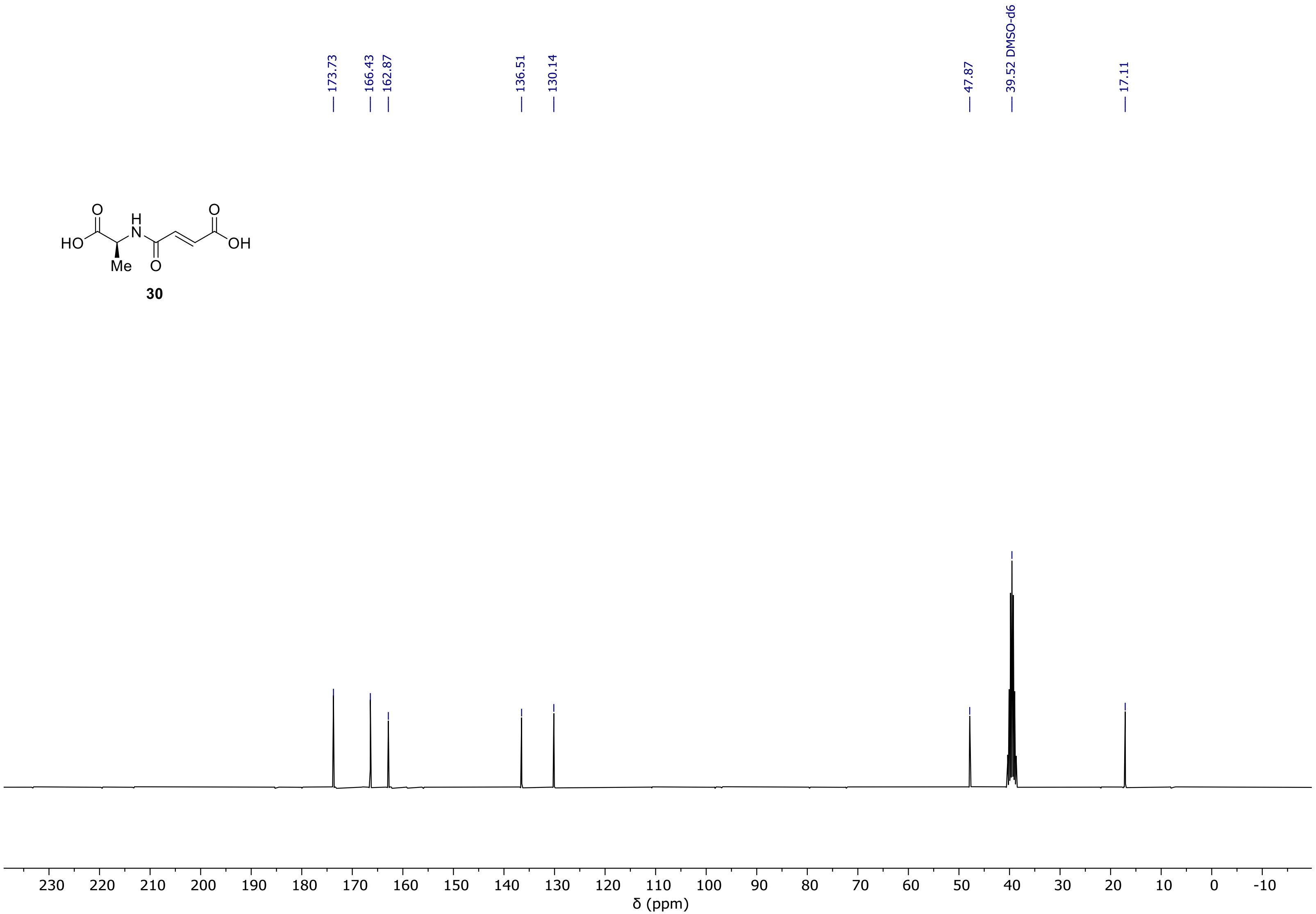

Fig. S25 ^{13}C-NMR spectrum of *L*-Ala-fumaric acid (**30**), measured in DMSO-d_6 at 75 MHz.

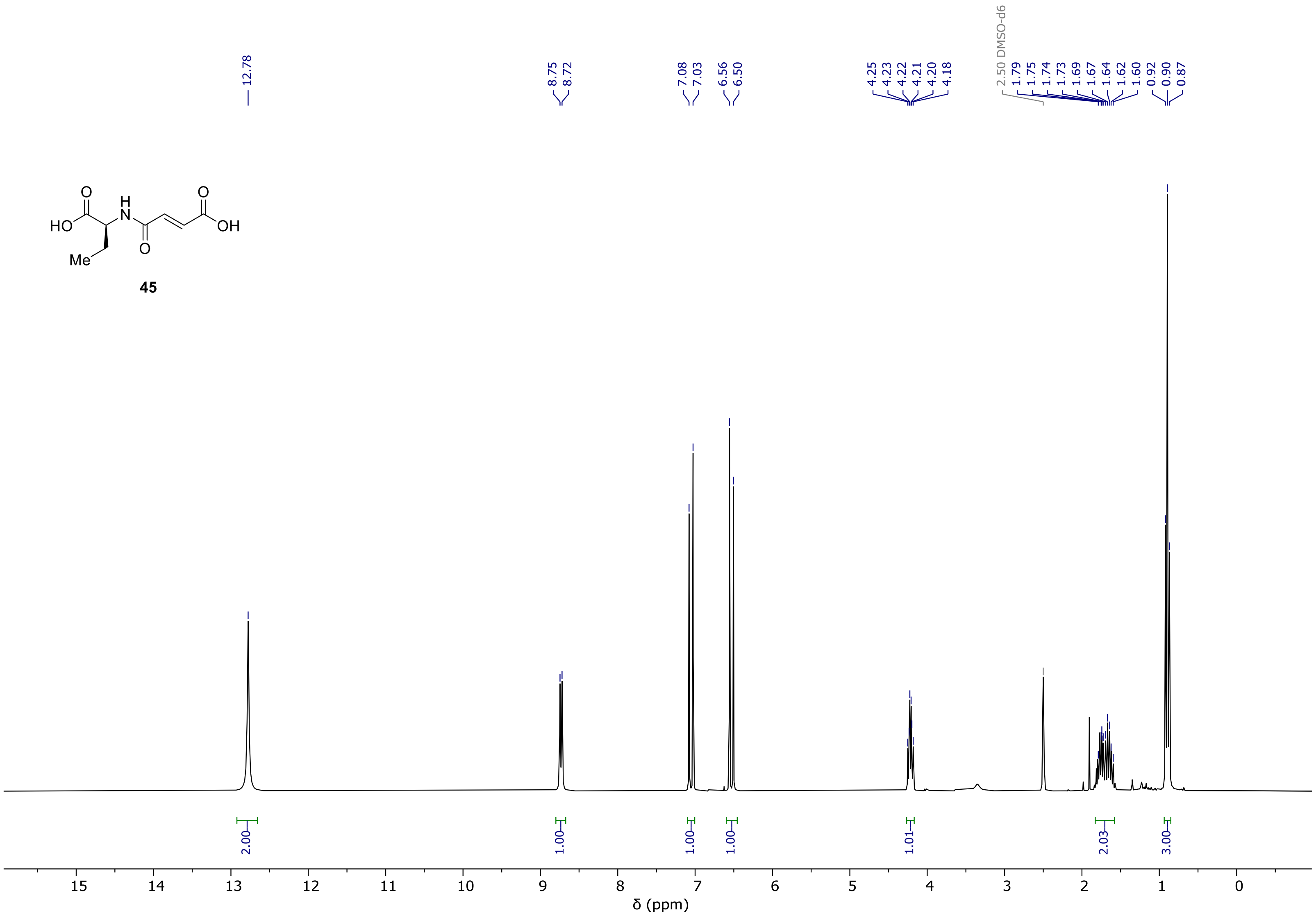

Fig. S26 ^{1}H-NMR spectrum of *L*-Abu-fumaric acid (**45**), measured in DMSO-d_6 at 300 MHz.

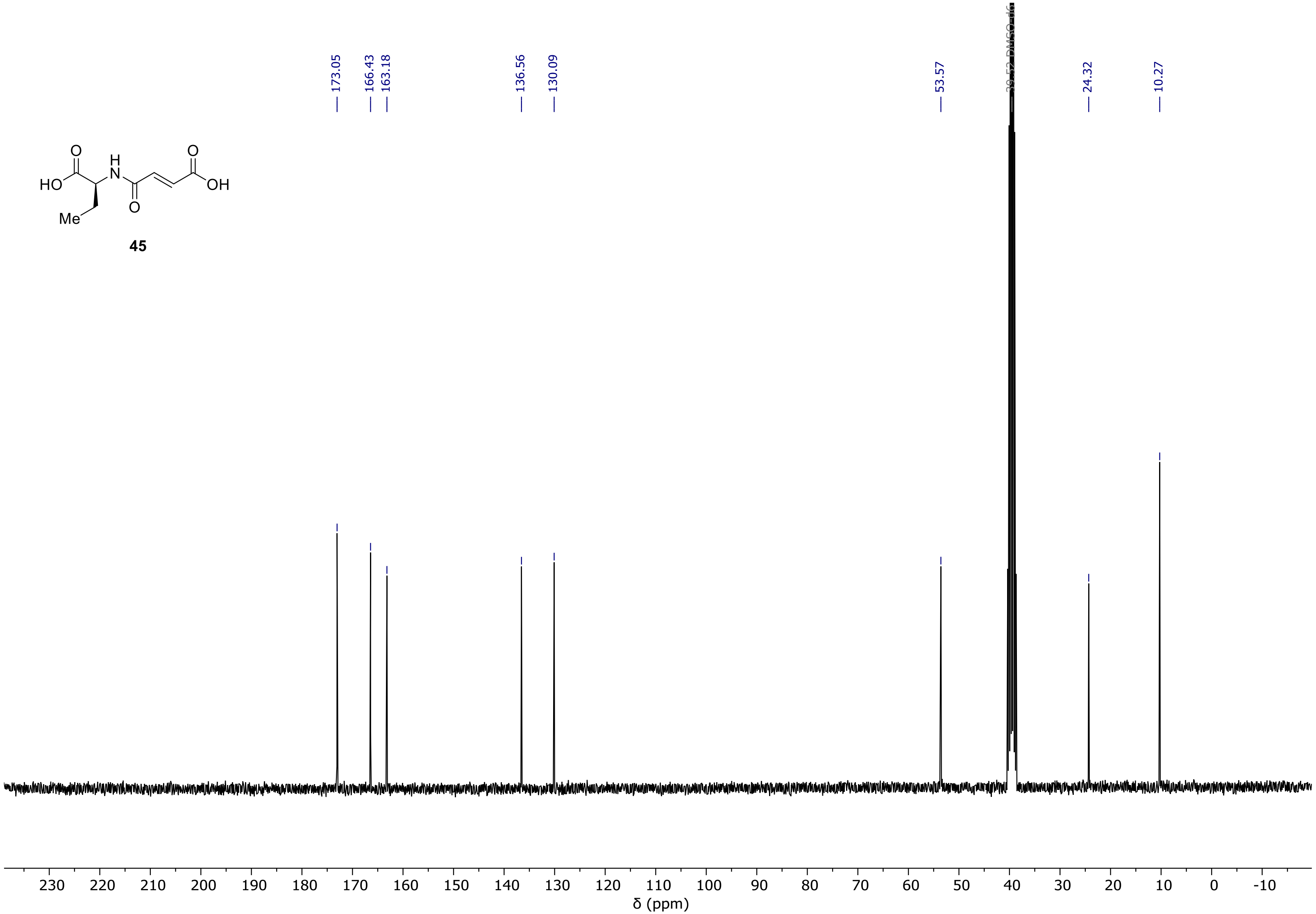

Fig. S27 ^{13}C-NMR spectrum of *L*-Abu-fumaric acid (**45**), measured in DMSO-d_6 at 75 MHz.

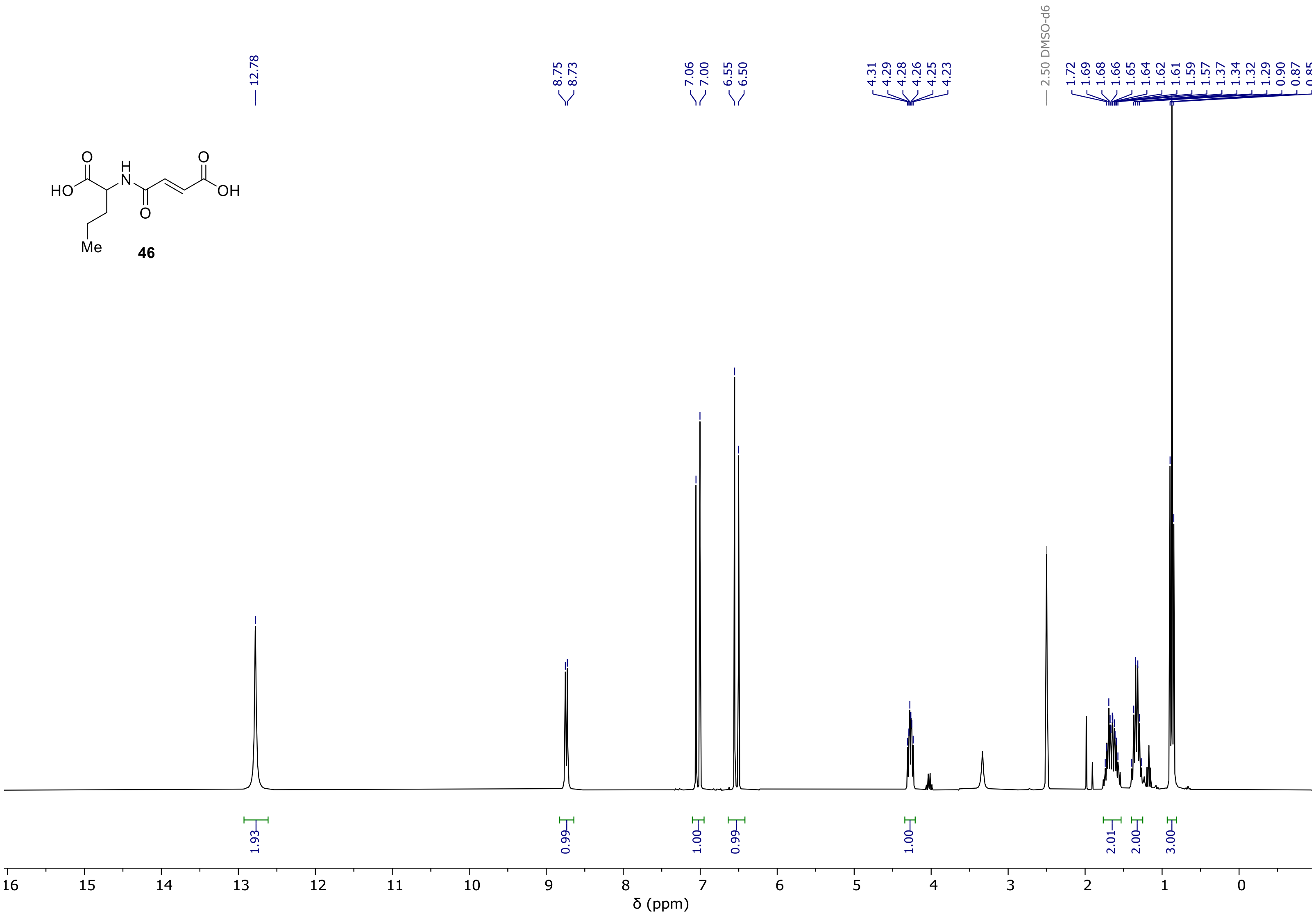

Fig. S28 ^{1}H-NMR spectrum of Nva-fumaric acid (**46**), measured in DMSO-d$_6$ at 300 MHz.

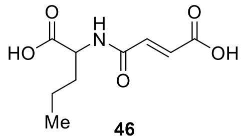

46

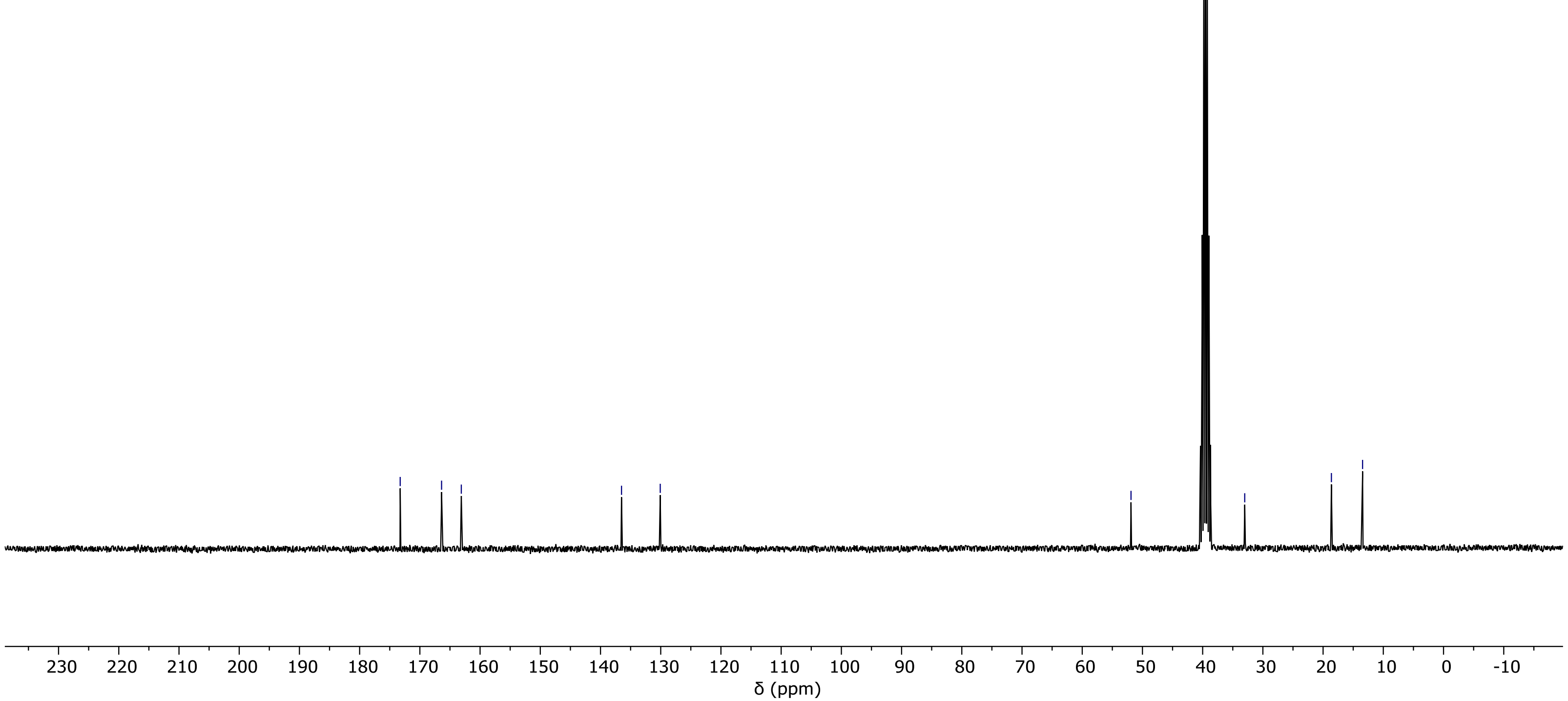

Fig. S29 ^{13}C-NMR spectrum of Nva-fumaric acid (**46**), measured in DMSO-d$_6$ at 75 MHz.

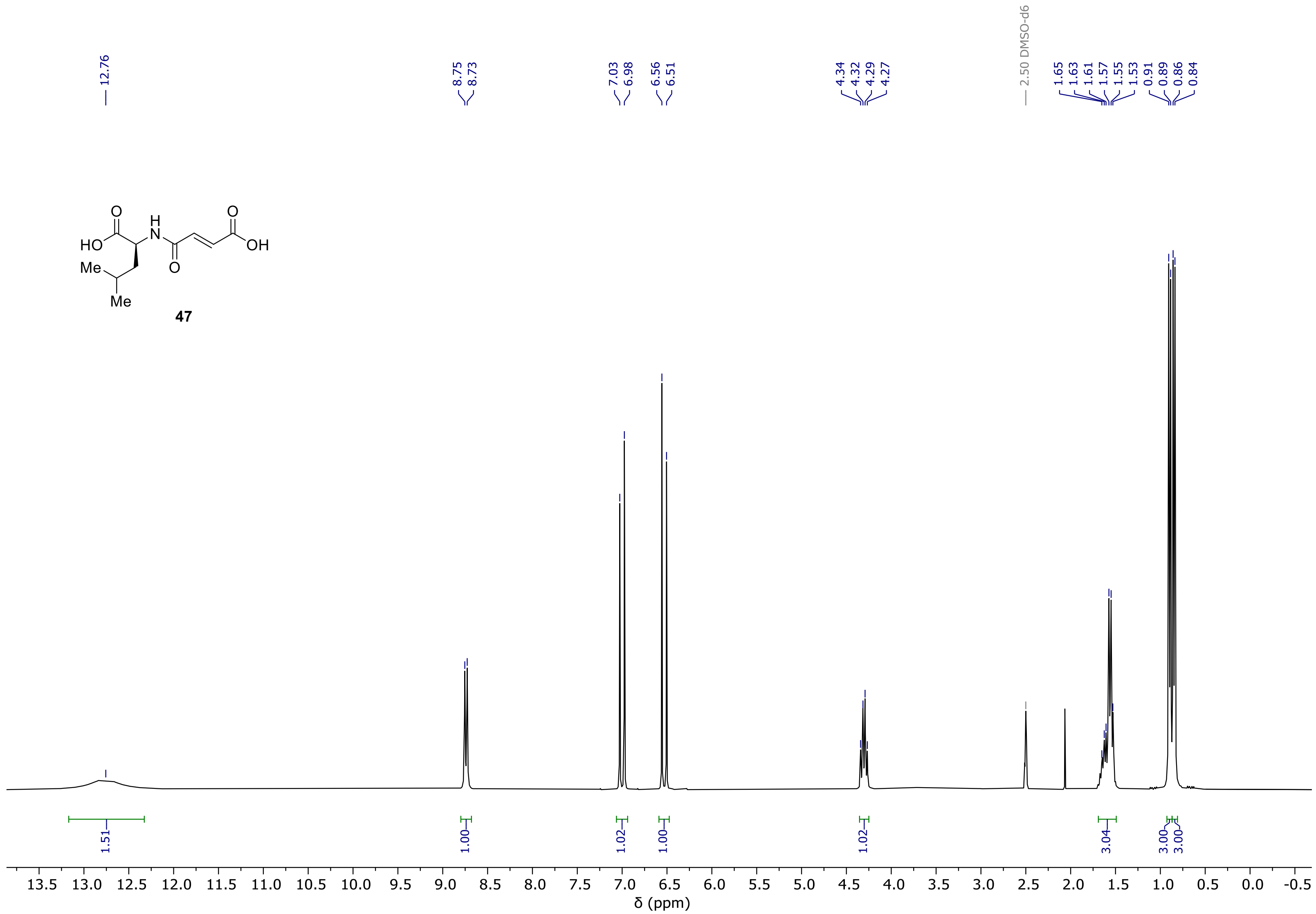

Fig. S30 ^{1}H-NMR spectrum of *L*-Leu-fumaric acid (**47**), measured in DMSO-d_6 at 300 MHz.

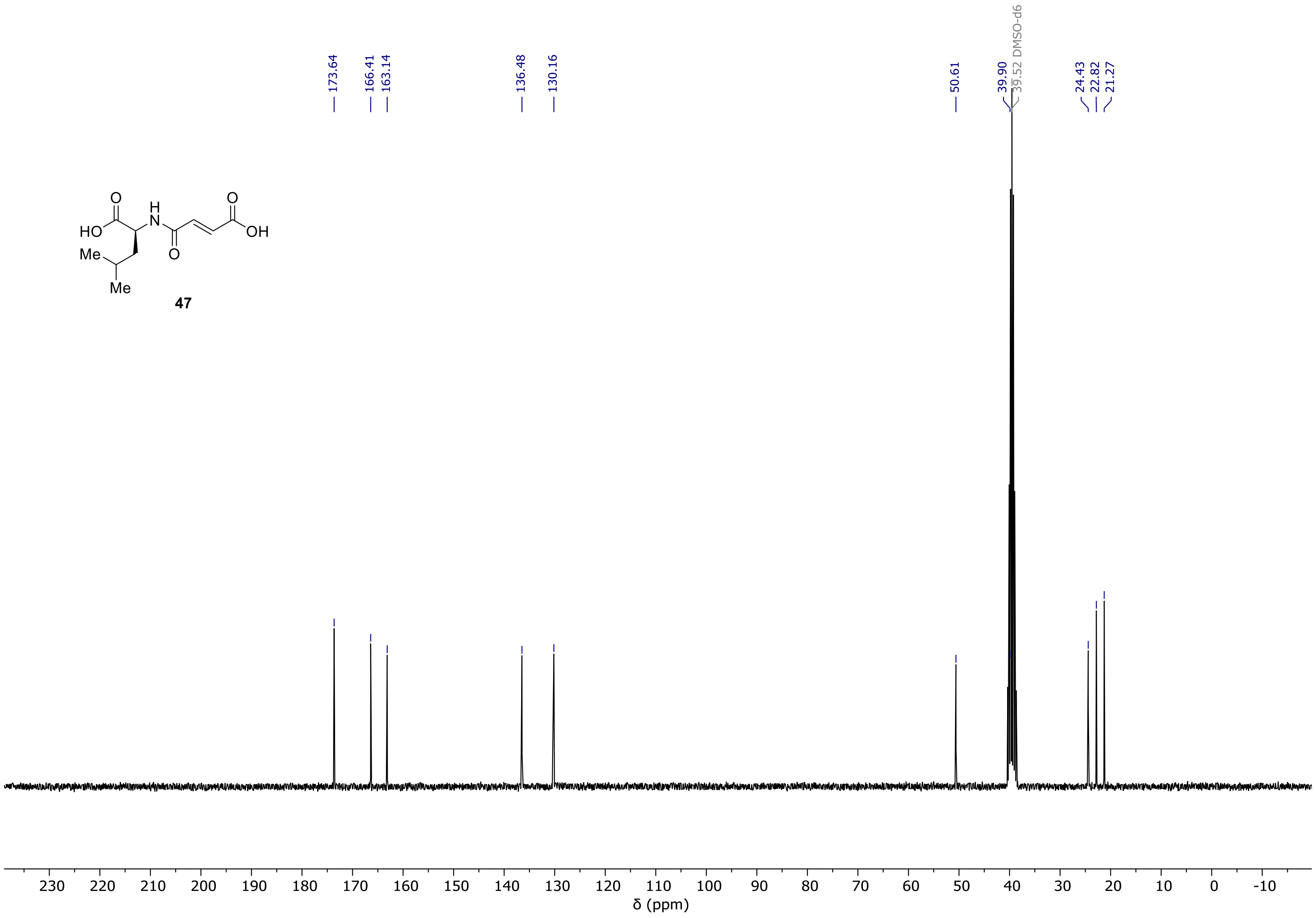

Fig. S31 ^{13}C-NMR spectrum of *L*-Leu-fumaric acid (**47**), measured in DMSO-d_6 at 75 MHz.

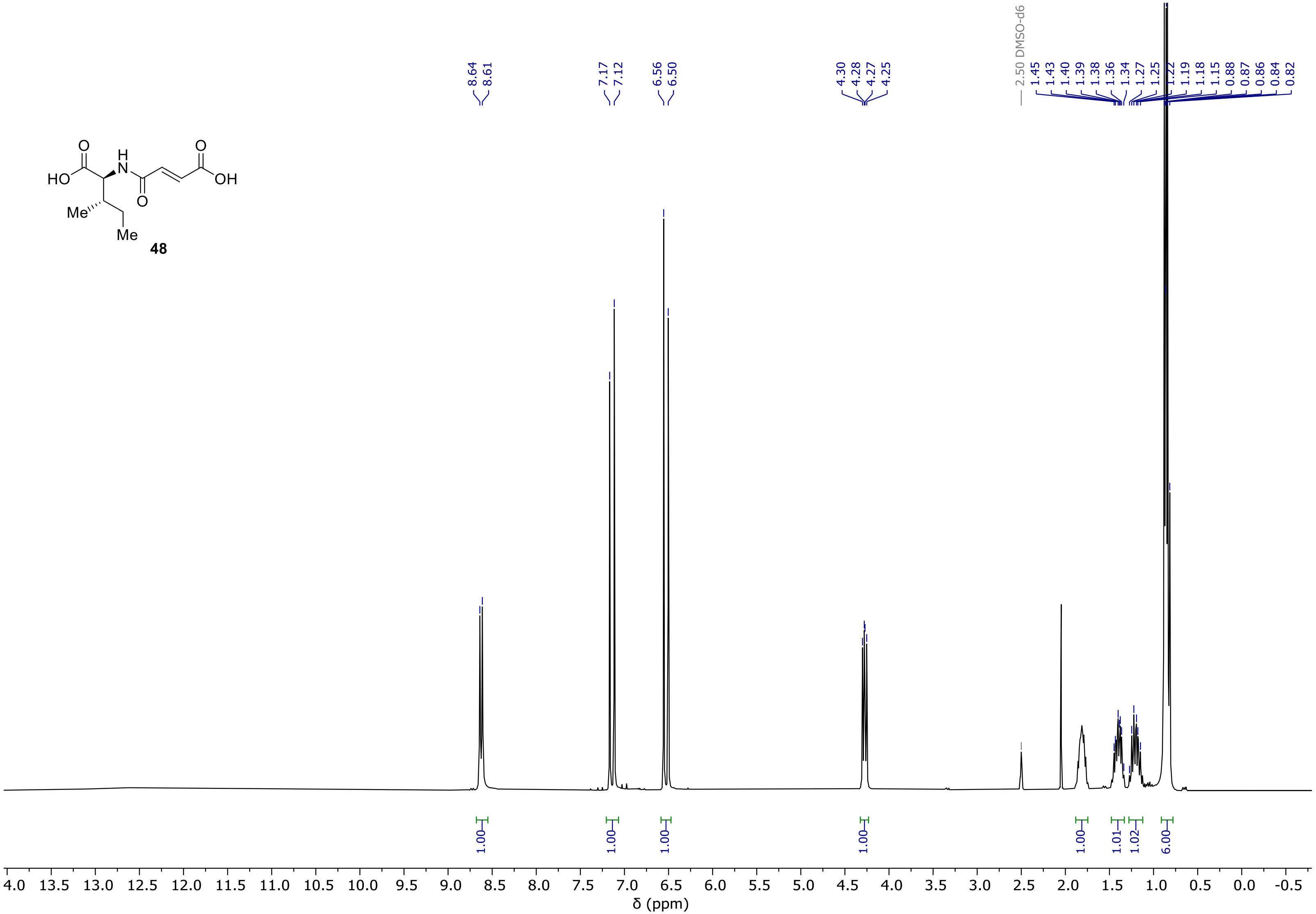

Fig. S32 ^{1}H-NMR spectrum of *L*-Ile-fumaric acid (**48**), measured in DMSO-d_6 at 300 MHz.

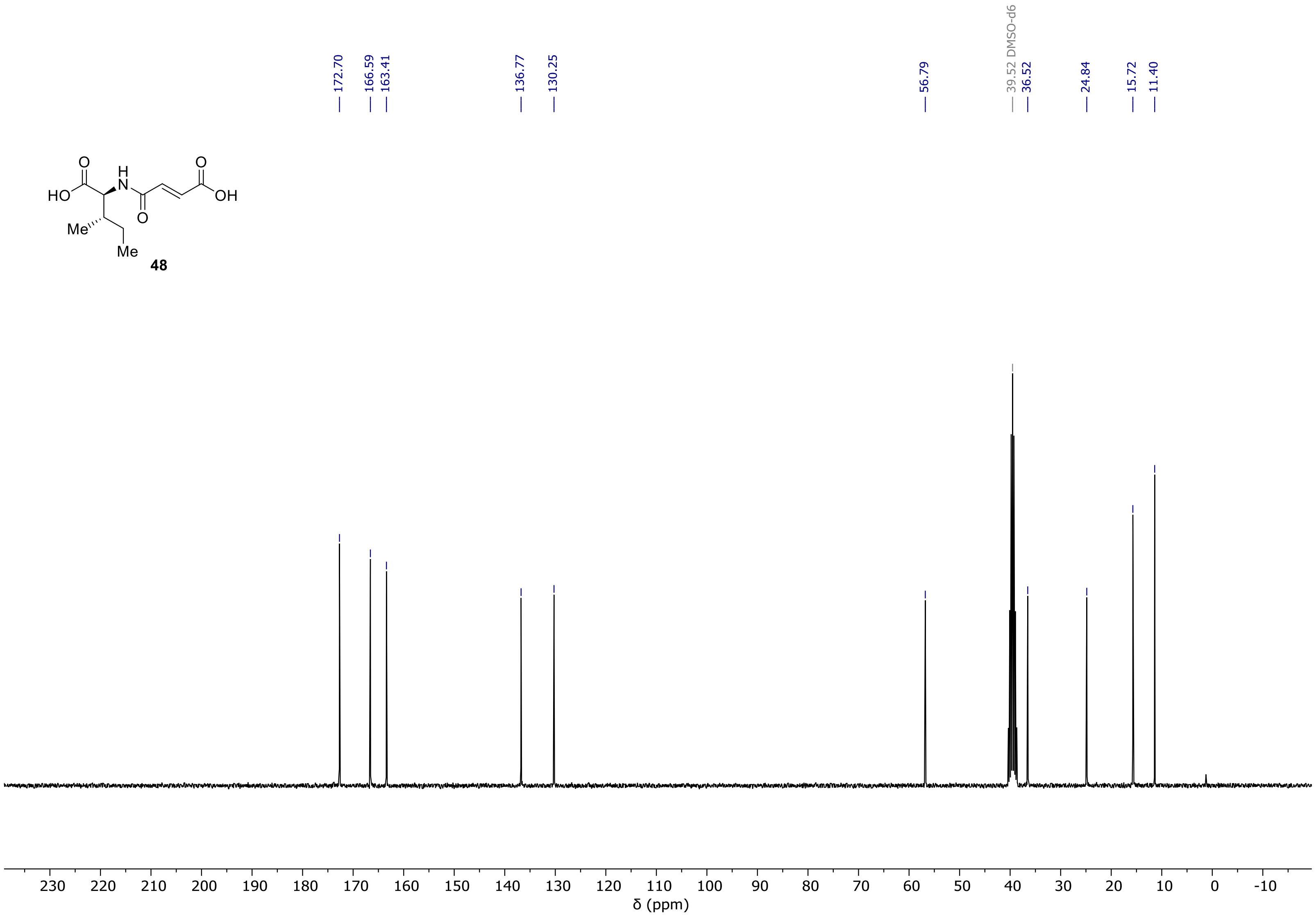

Fig. S33 ^{13}C-NMR spectrum of *L*-Ile-fumaric acid (**48**), measured in DMSO-d$_6$ at 75 MHz.

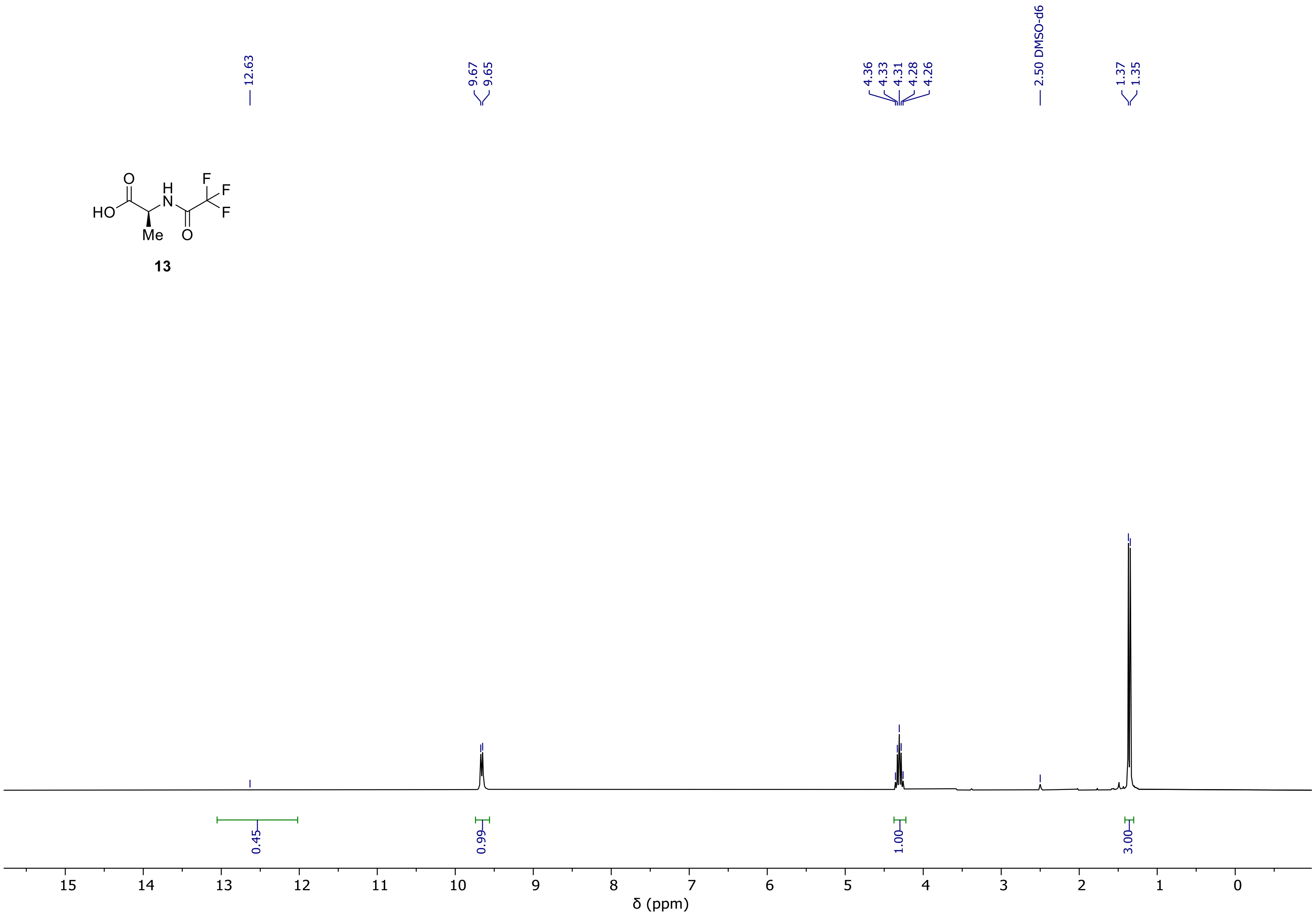

Fig. S34 ^{1}H-NMR spectrum of 2,2,2-trifluroacetyl-*L*-alanine (**13**), measured in DMSO-d_6 at 300 MHz.

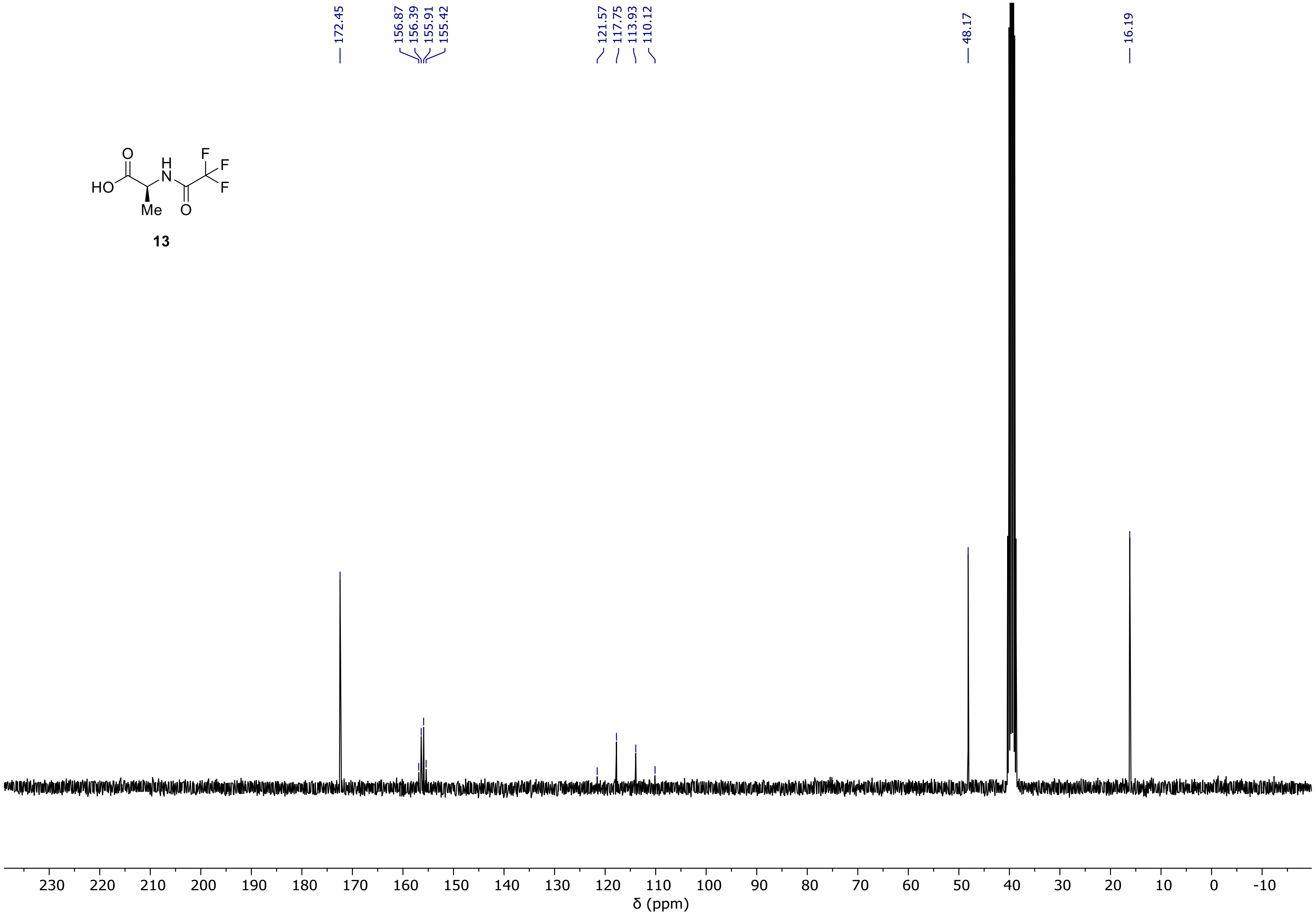

Fig. S35 ^{13}C-NMR spectrum of 2,2,2-trifluroacetyl-*L*-alanine (**13**), measured in DMSO-d_6 at 75 MHz.

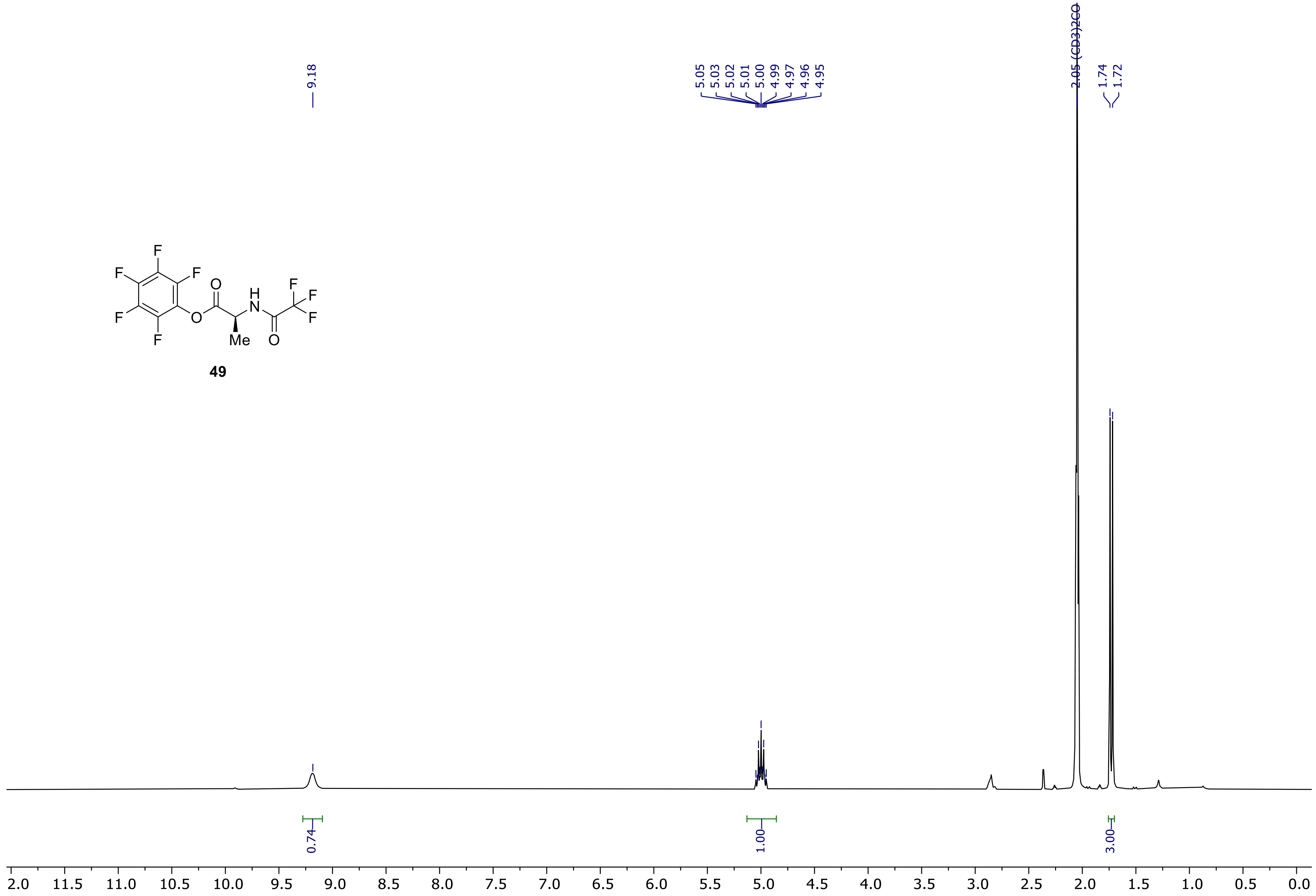

Fig. S36 ^{1}H-NMR spectrum of 2,2,2-trifluroacetyl-*L*-Ala-Pfp (**49**), measured in acetone-d_6 at 300 MHz.

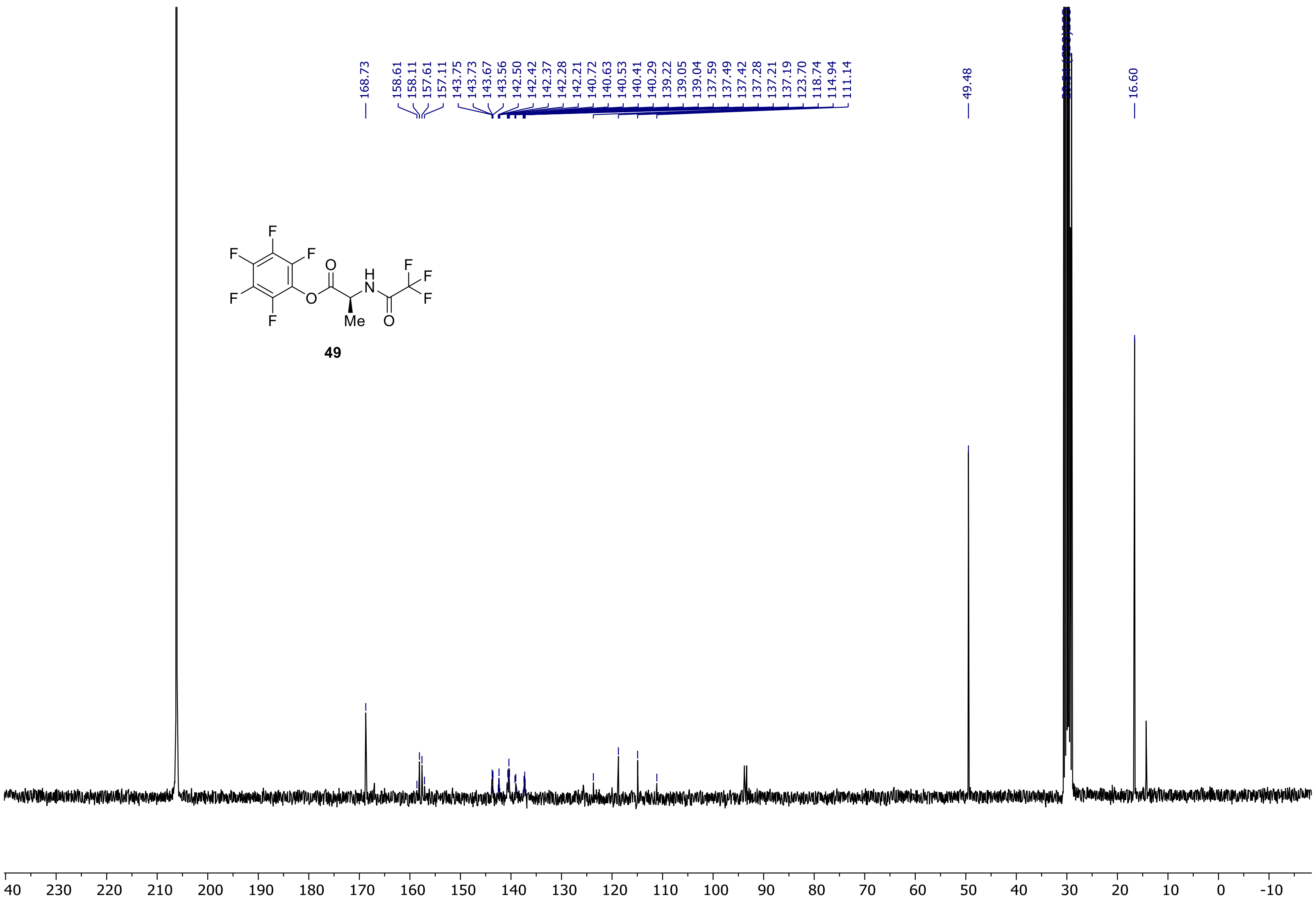

Fig. S37 ^{13}C-NMR spectrum of 2,2,2-trifluroacetyl-*L*-Ala-Pfp (**49**), measured in acetone-d_6 at 75 MHz.

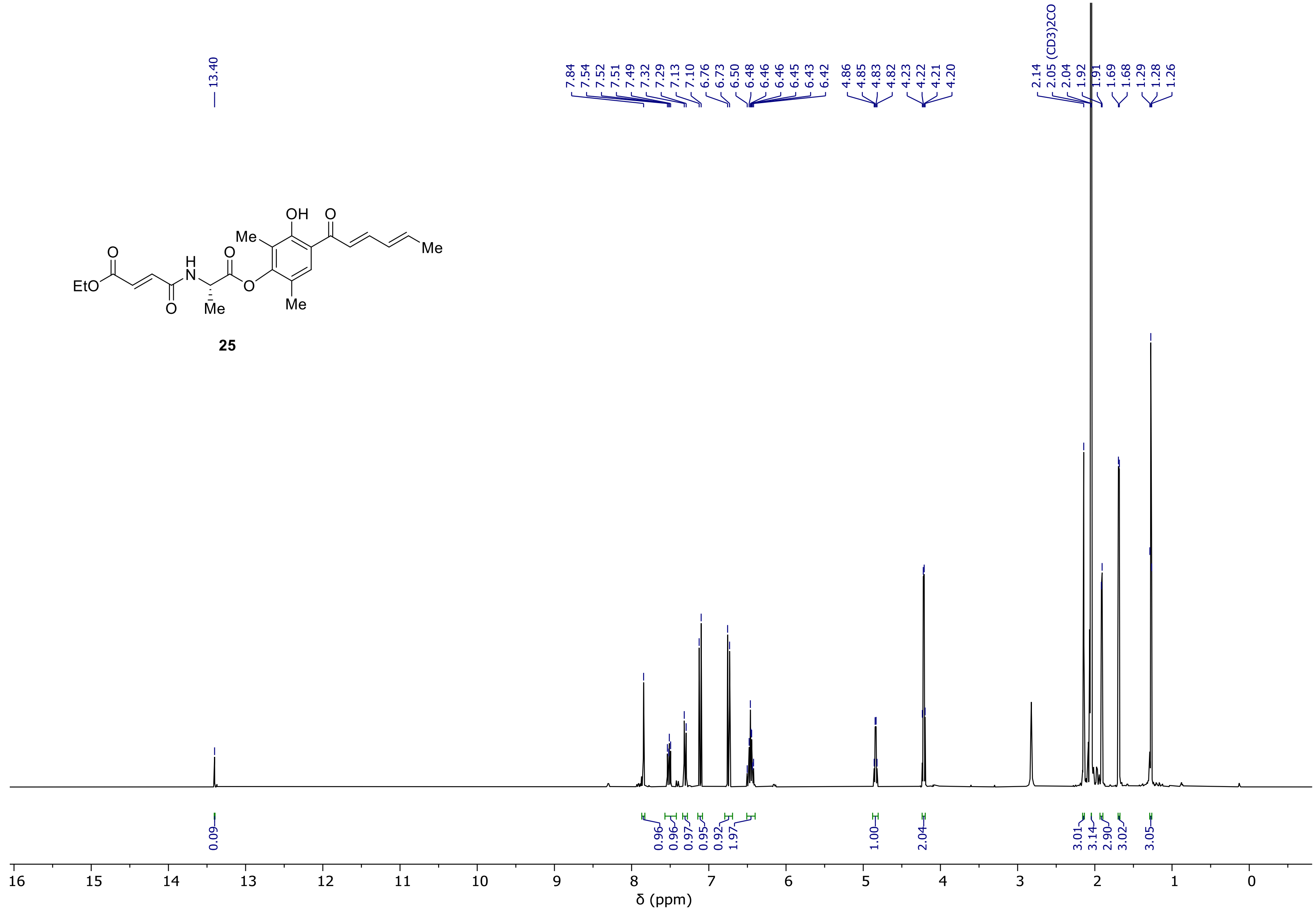

Fig. S38 ^{1}H-NMR spectrum of sorbicillin-*L*-Ala-ethylfumarate (**25**), measured in acetone-d$_6$ at 600 MHz.

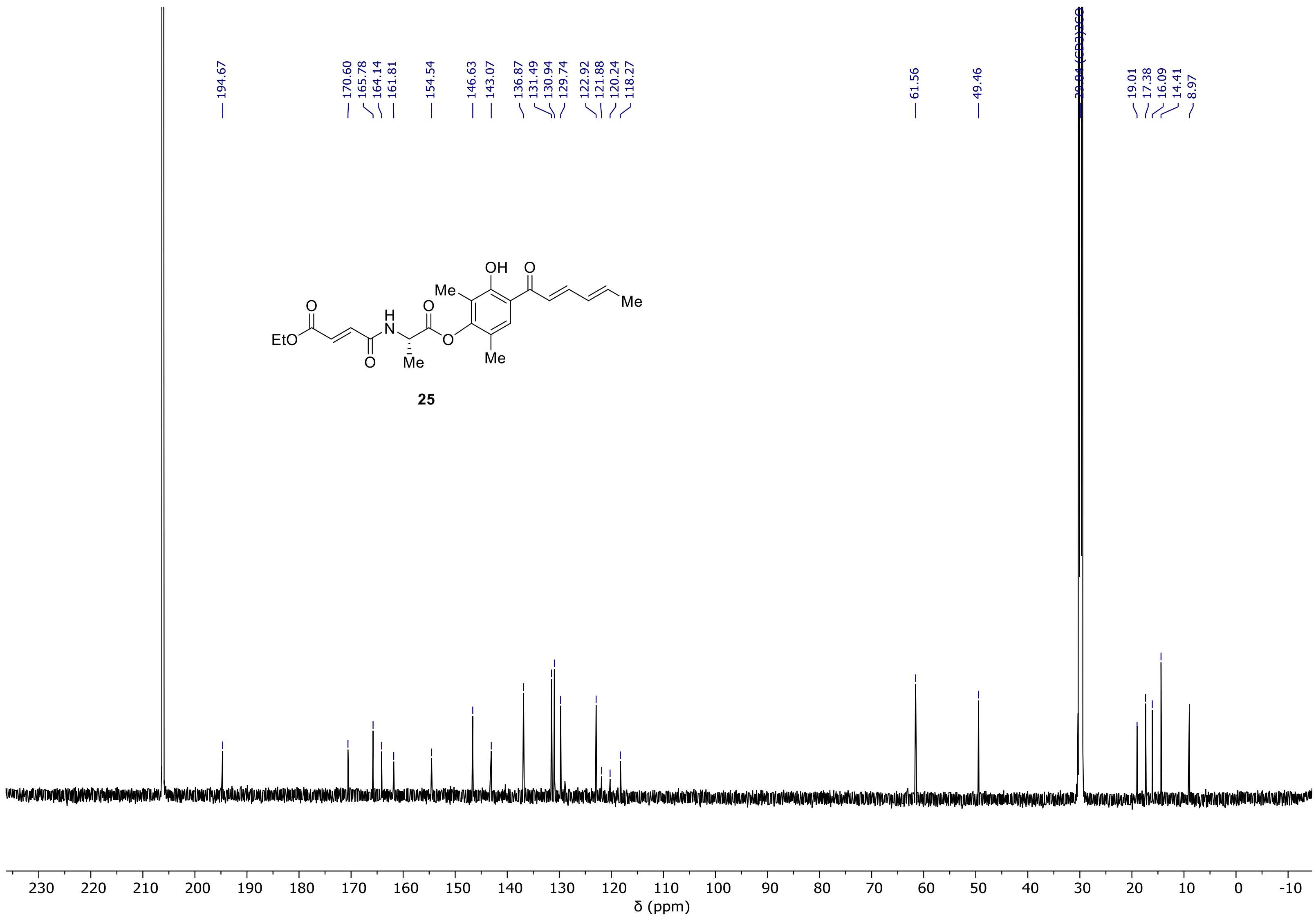

Fig. S39 ^{13}C-NMR spectrum of sorbicillin-*L*-Ala-ethylfumarate (**25**), measured in acetone-d_6 at 151 MHz.

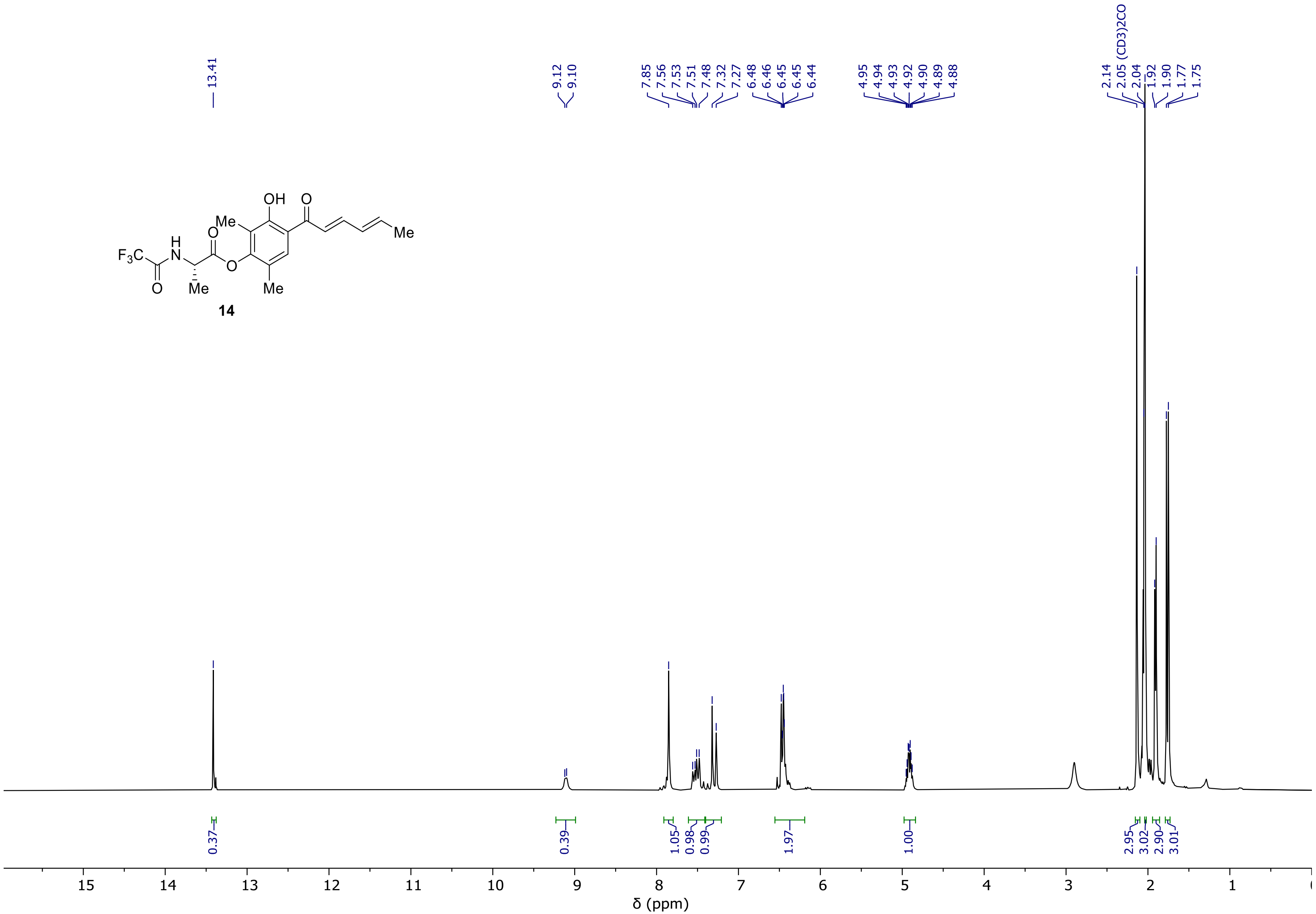

Fig. S40 ^{1}H-NMR spectrum of sorbicillin-*L*-Ala-TFA (**14**), measured in acetone-d_6 at 600 MHz.

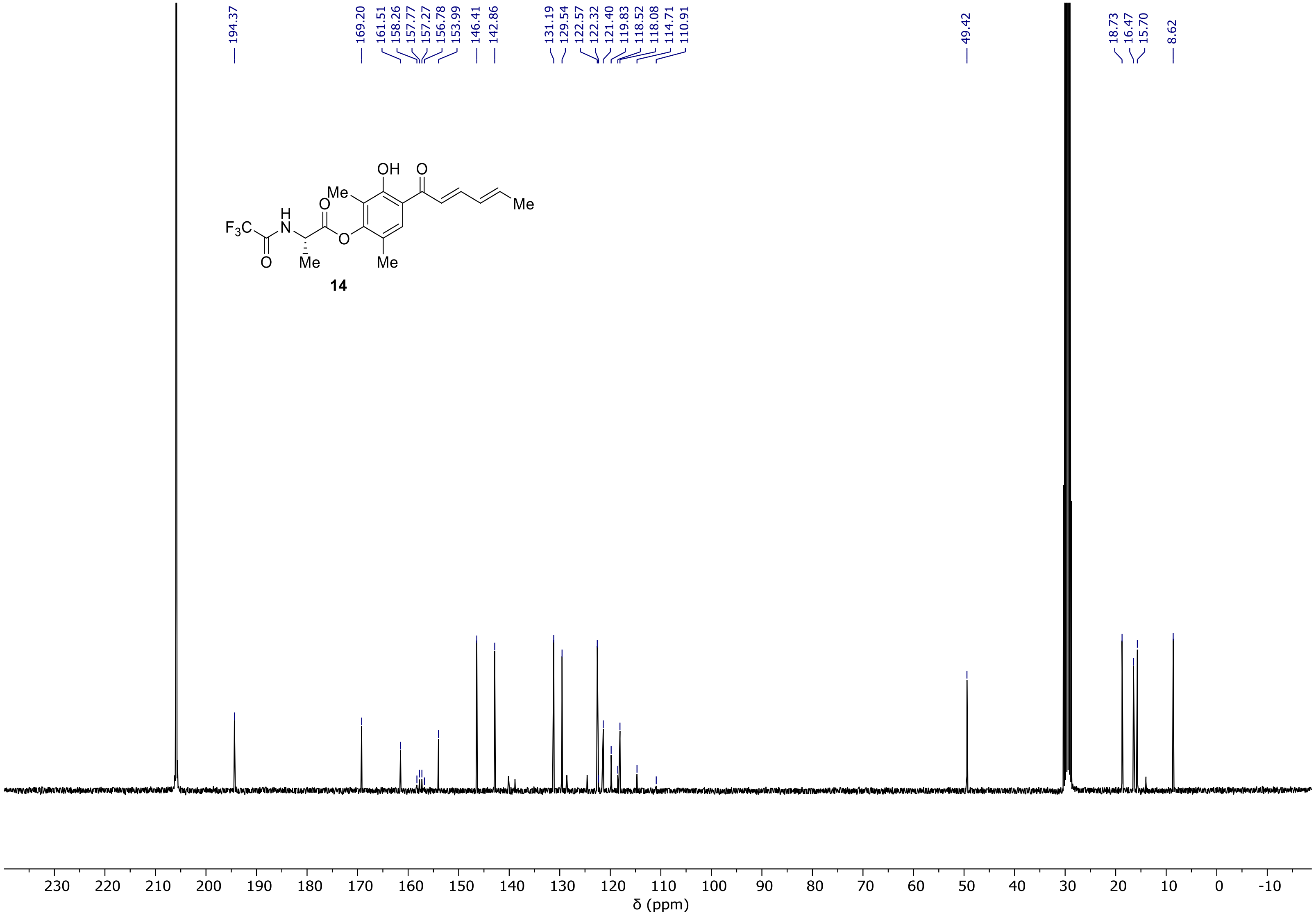

Fig. S41 ^{13}C-NMR spectrum of sorbicillin-*L*-Ala-TFA (**25**), measured in acetone-d_6 at 151 MHz.

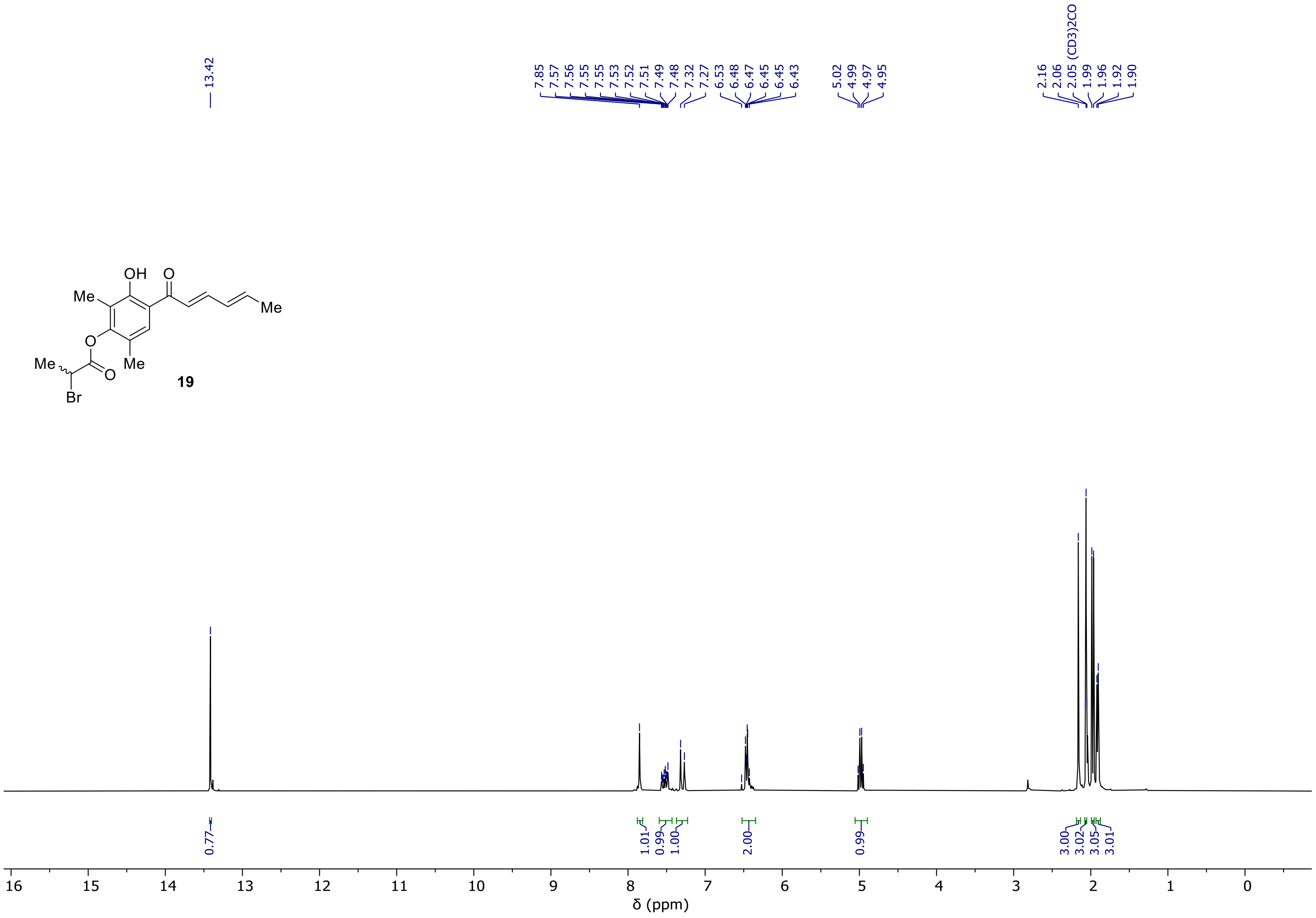

Fig. S42 ^{1}H-NMR spectrum of sorbicillin-bromopropanat (**19**), measured in acetone-d_6 at 300 MHz.

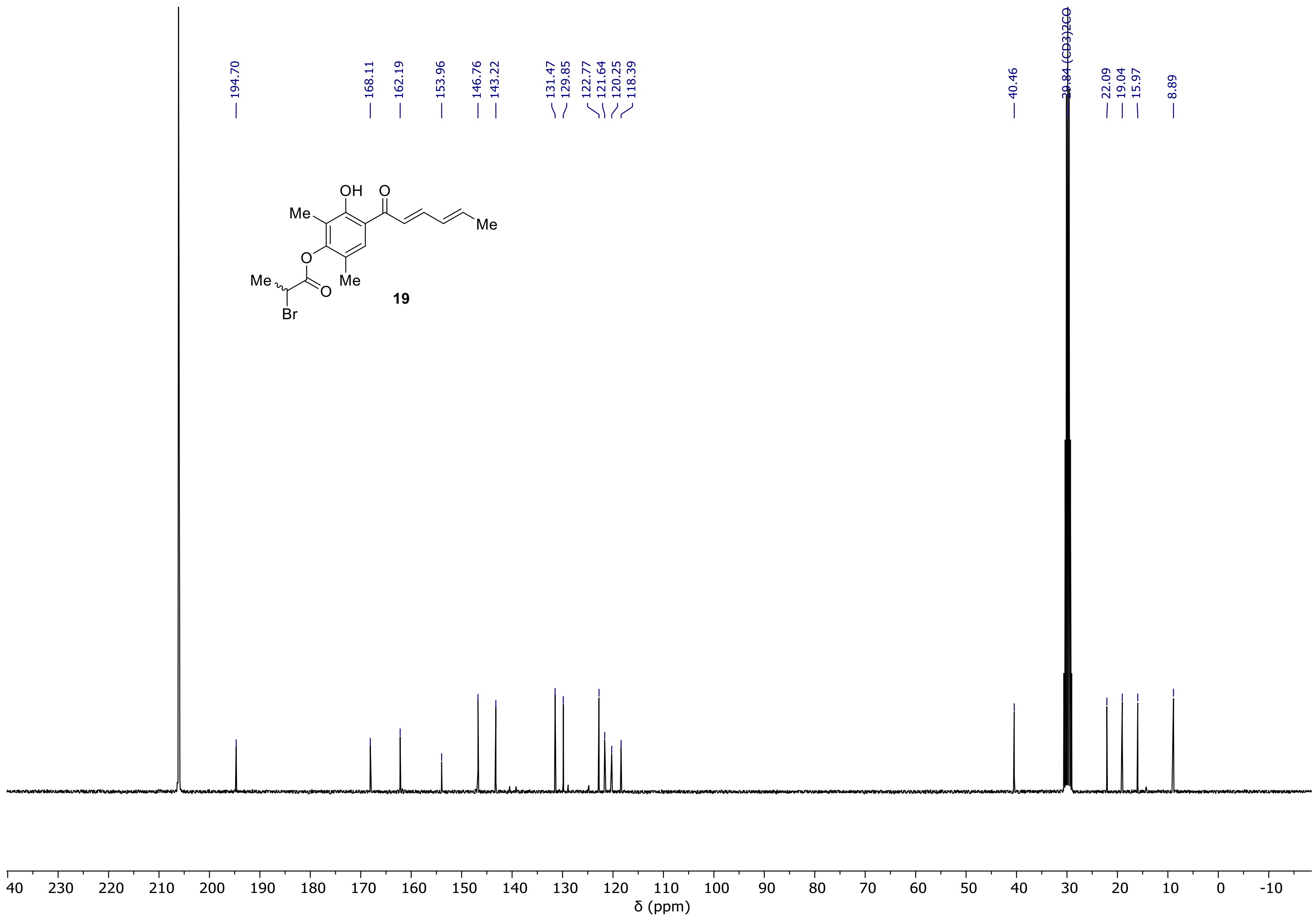

Fig. S43 ^{13}C-NMR spectrum of sorbicillin-bromopropanat (**19**), measured in acetone-d_6 at 75 MHz.

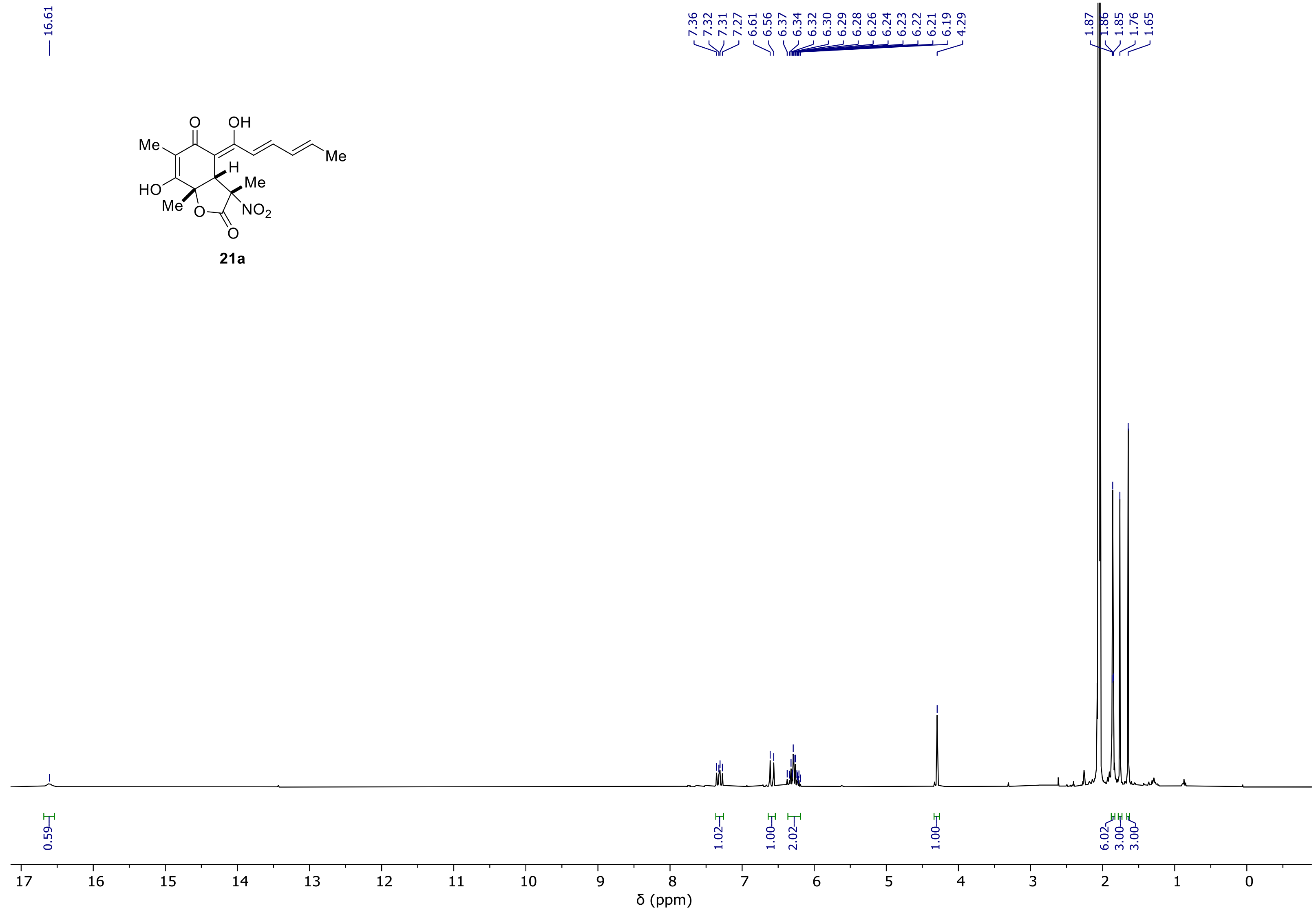

Fig. S44 ^{1}H-NMR spectrum of nitrosorbicillactone (**21a**), measured in acetone-d_6 at 300 MHz.

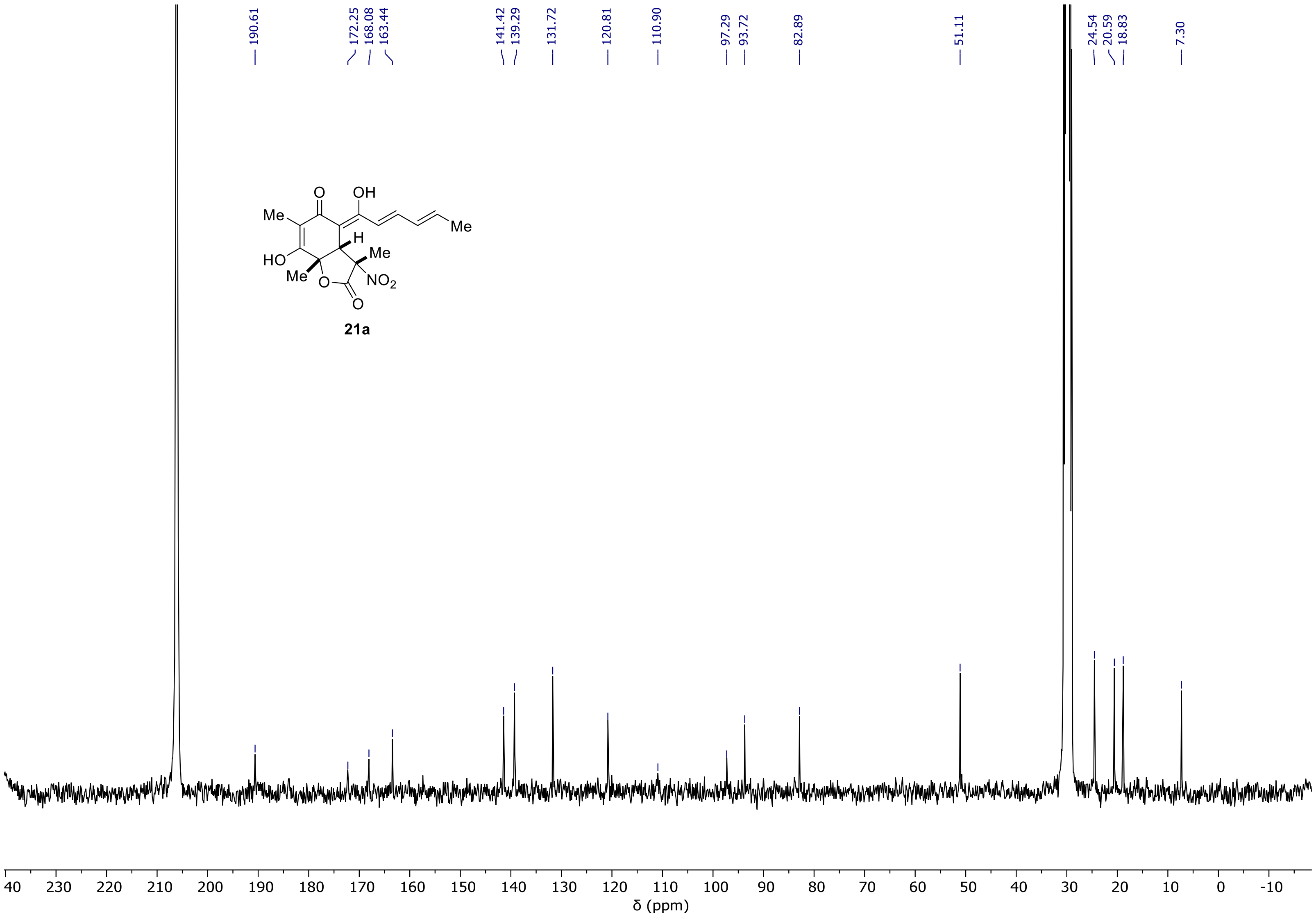

Fig. S45 ^{13}C-NMR spectrum of nitrosorbicillactone (**21a**), measured in acetone-d_6 at 75 MHz.

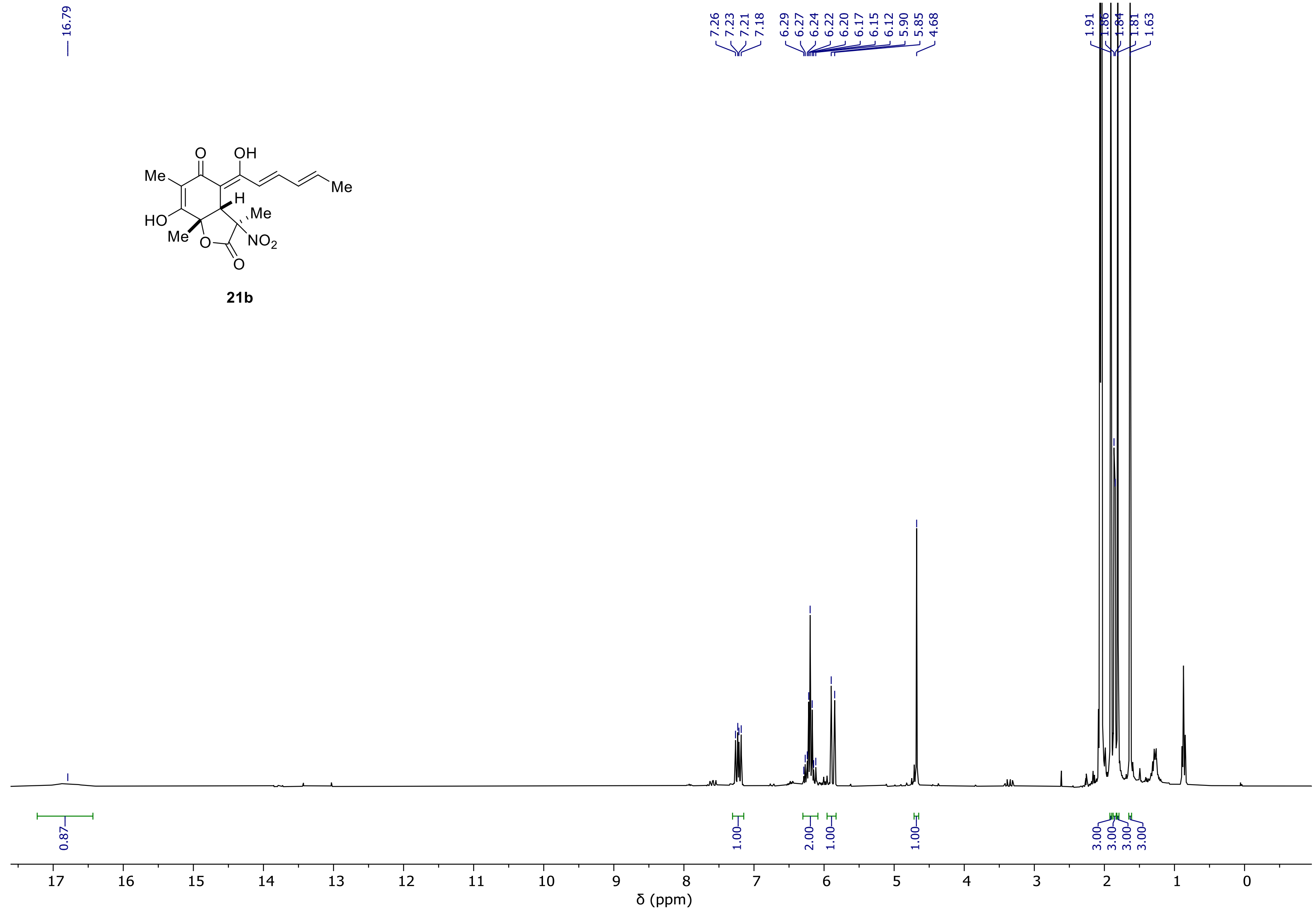

Fig. S46 ^{1}H-NMR spectrum of epi-nitrosorbicillactone (**21b**), measured in acetone-d_6 at 300 MHz.

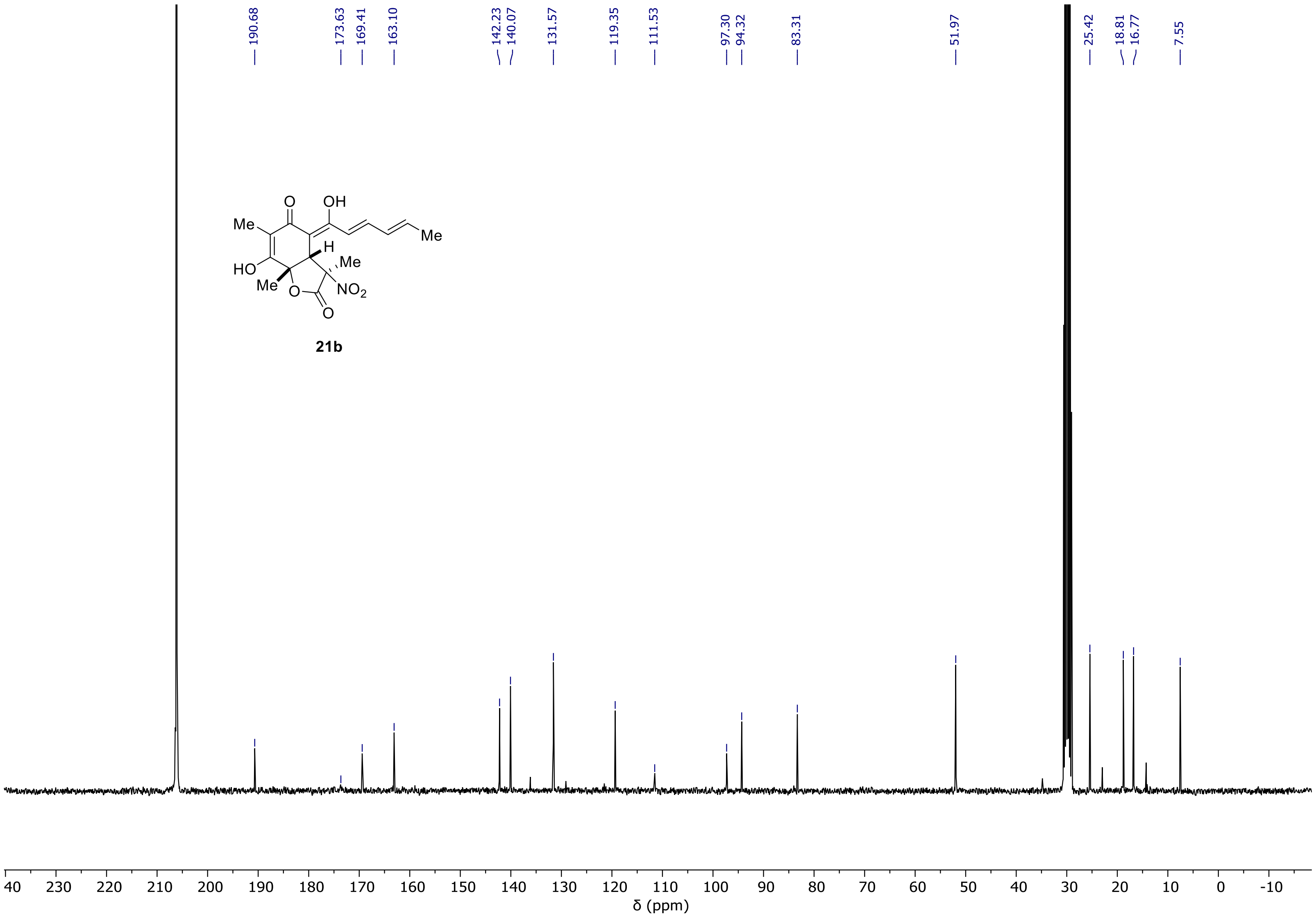

Fig. S47 ^{13}C-NMR spectrum of epi-nitrosorbicillactone (**21b**), measured in acetone-d_6 at 75 MHz.

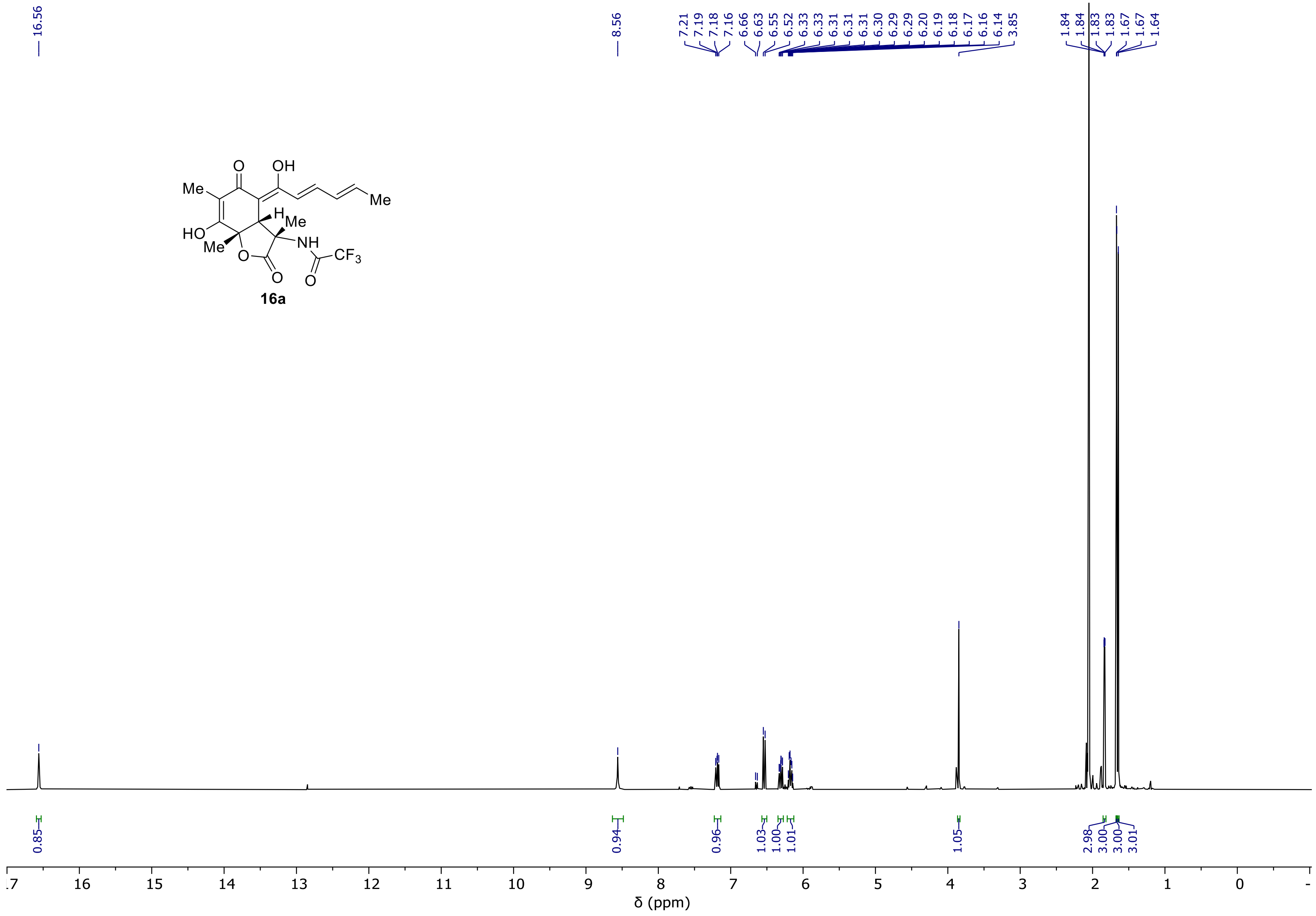

Fig. S48 ^{1}H-NMR spectrum of (-)-sorbicillactoneTFA (**16a**), measured in acetone-d$_6$ at 600 MHz.

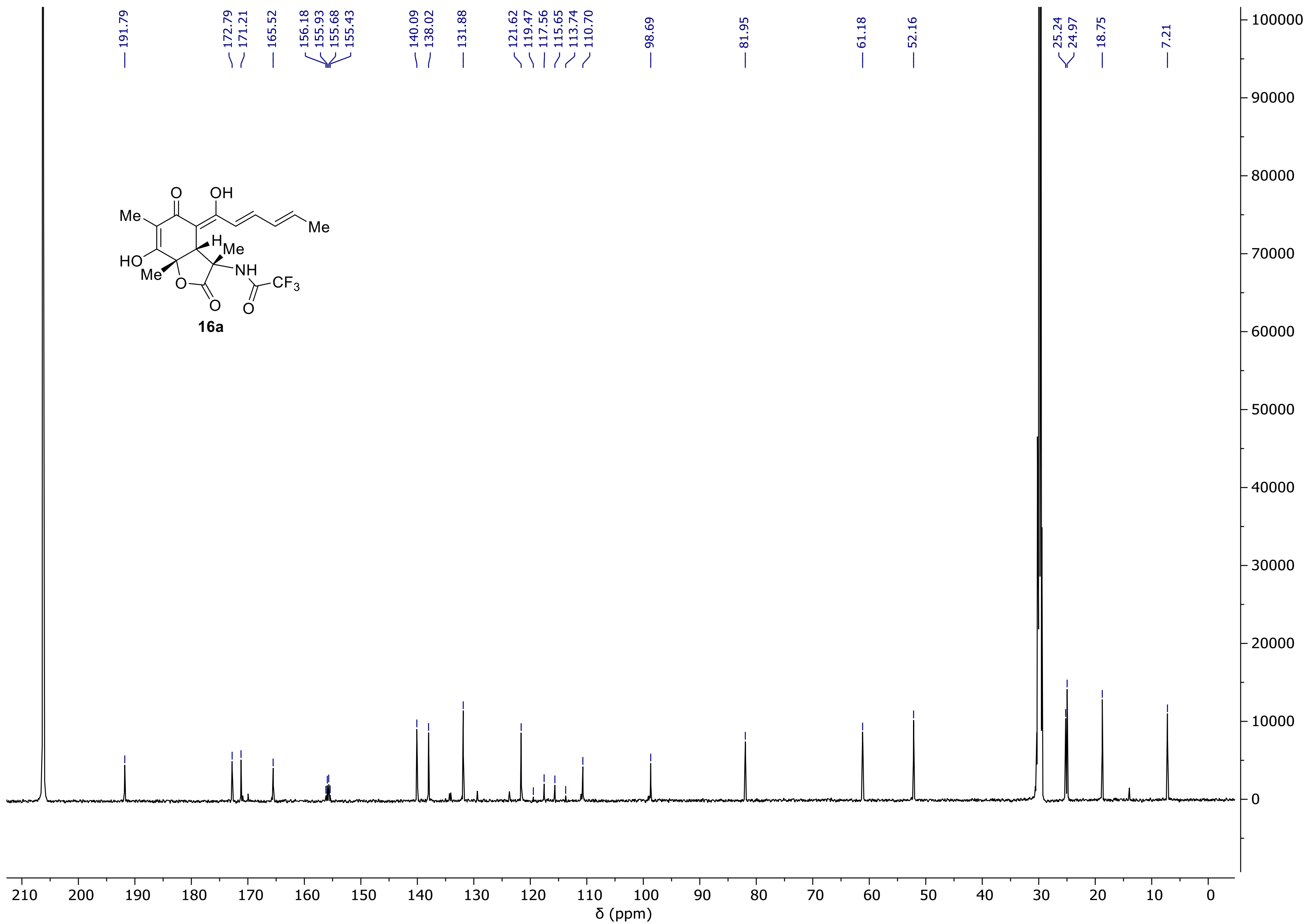

Fig. S49 ^{13}C-NMR spectrum of (-)-sorbicillactoneTFA (**16a**), measured in acetone-d_6 at 151 MHz.

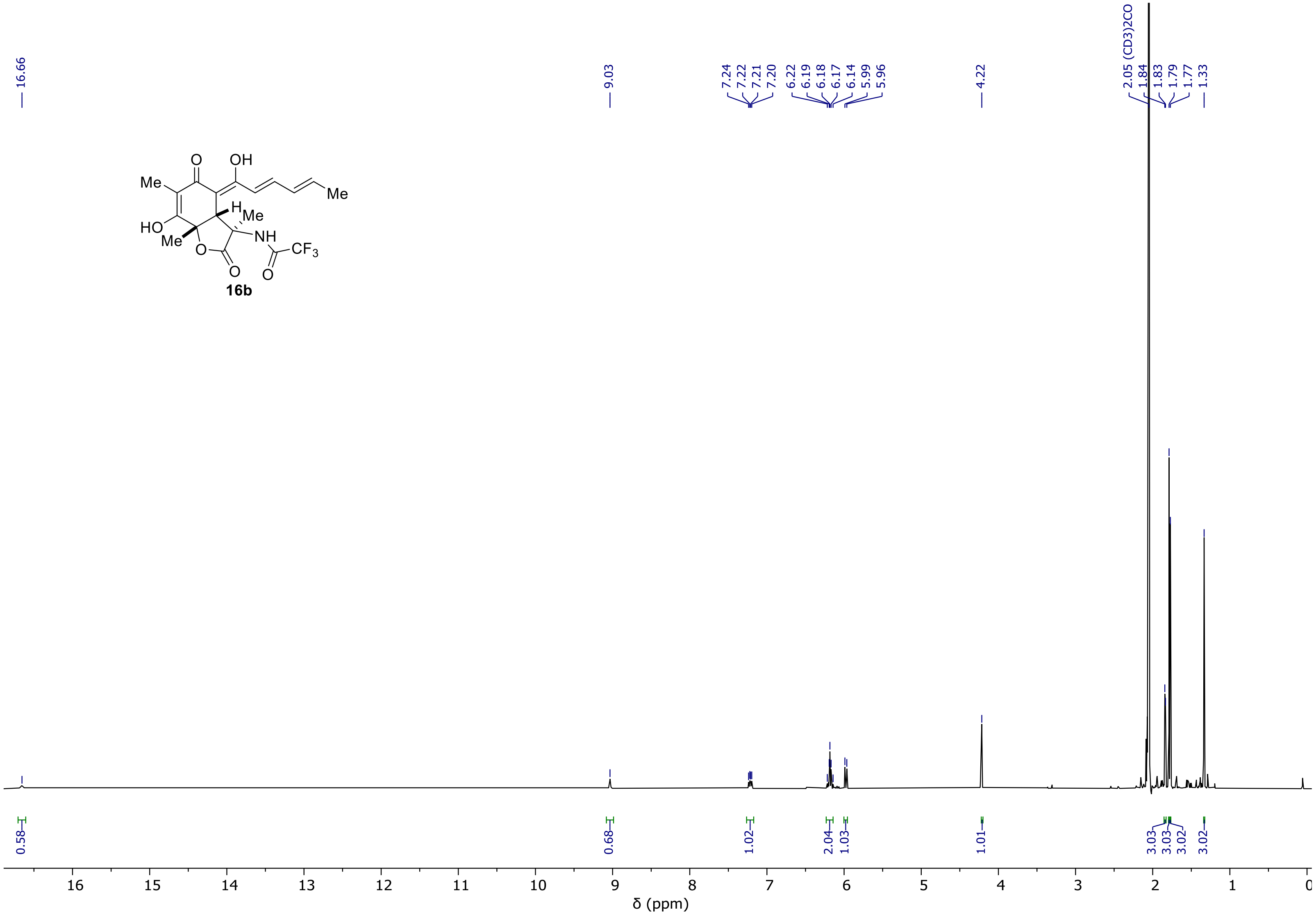

Fig. S50 ^{1}H-NMR spectrum of epi-sorbicillactoneTFA (**16b**), measured in acetone-d_6 at 600 MHz.

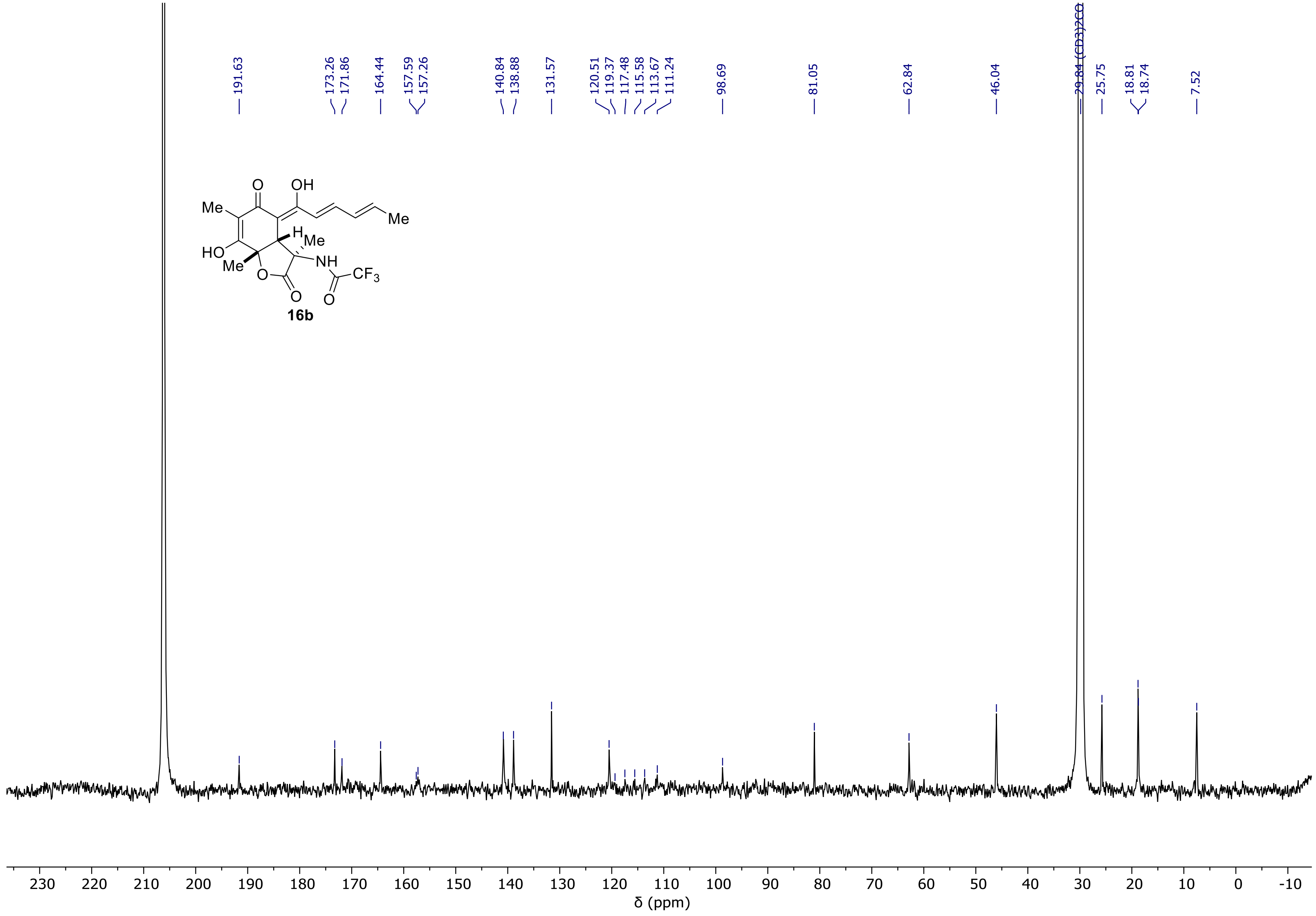

Fig. S51 ^{13}C-NMR spectrum of epi-sorbicillactoneTFA (**16b**), measured in acetone-d_6 at 151 MHz.

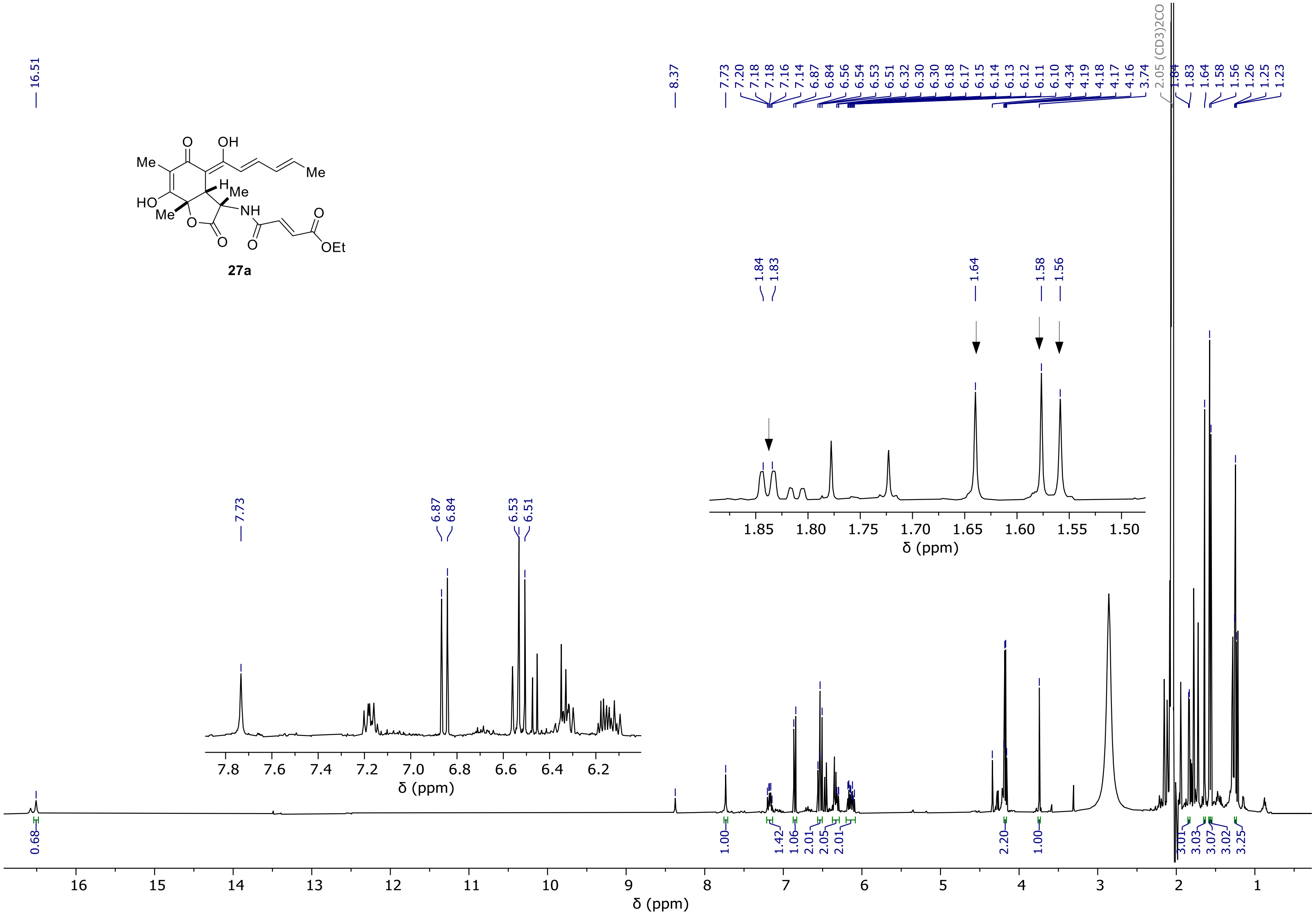

Fig. S52 ^{1}H-NMR spectrum of sorbicillactone ethyl ester (**27a**), measured in acetone-d_6 at 600 MHz.

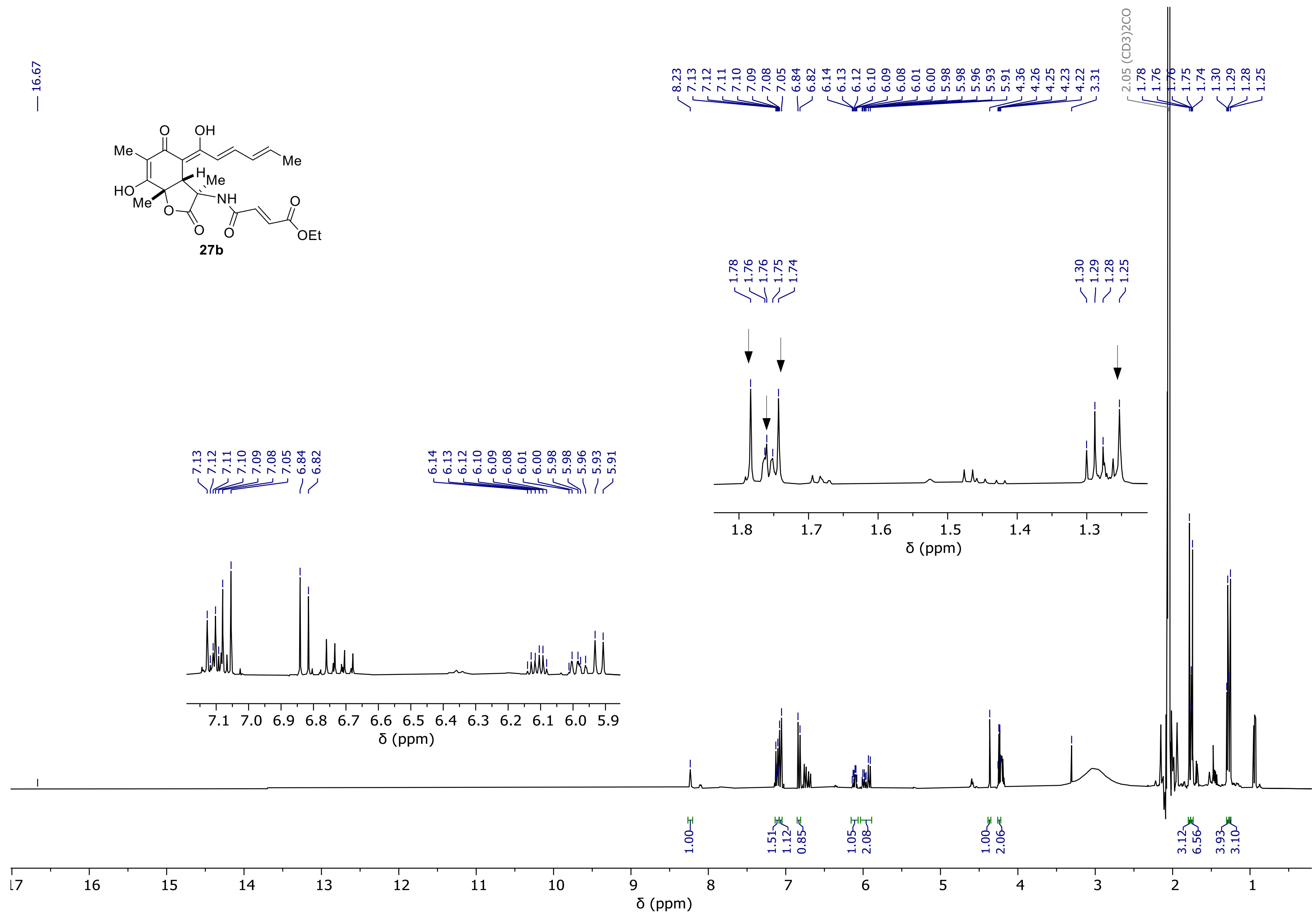

Fig. S53 ^{1}H-NMR spectrum of epi-sorbicillactone ethyl ester (**27b**), measured in acetone-d$_6$ at 600 MHz.

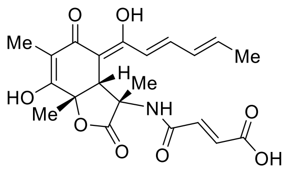

7

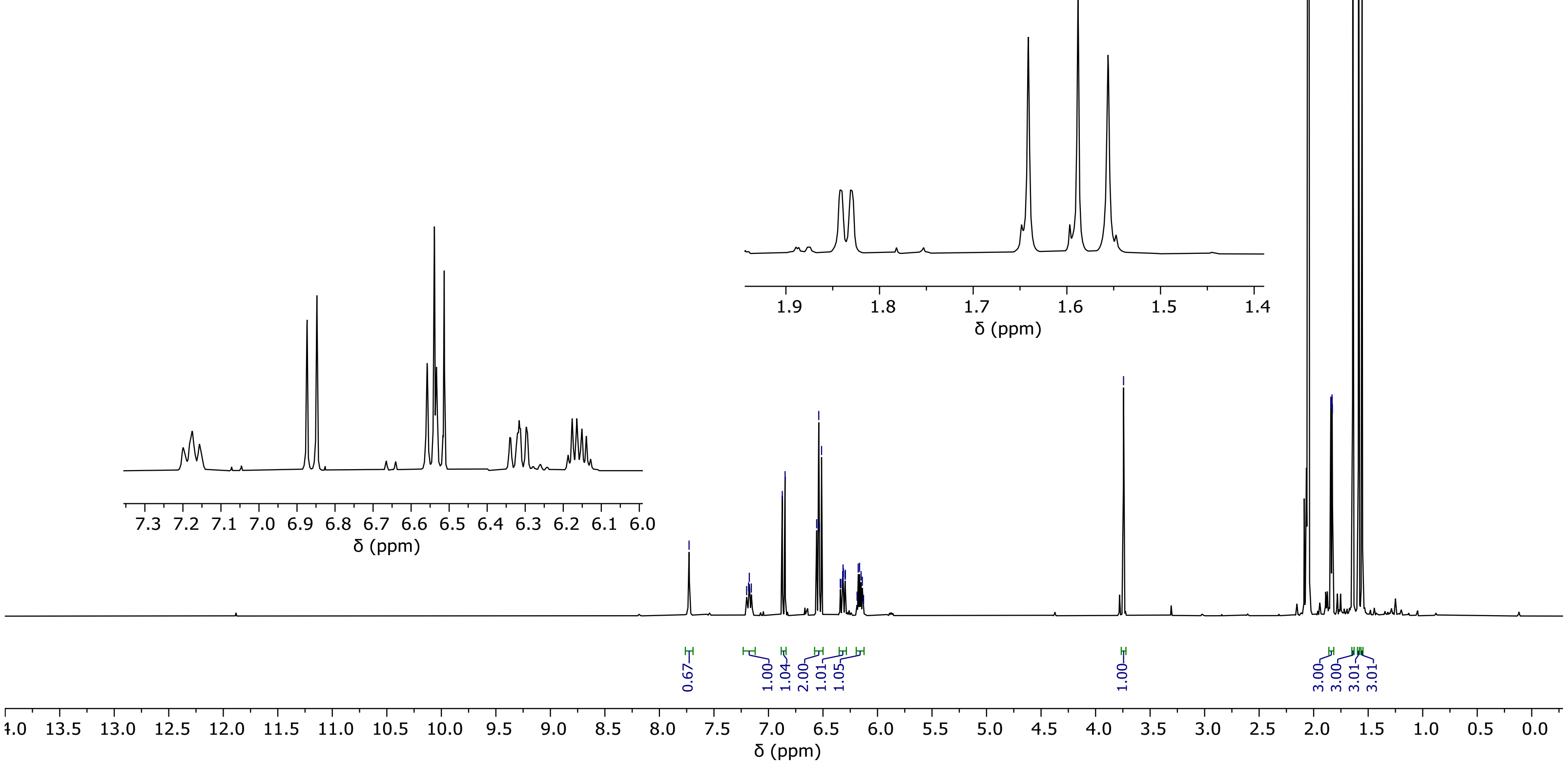

Fig. S54 ^{1}H-NMR spectrum of sorbicillactone A (**7**), measured in acetone-d_6 at 600 MHz.

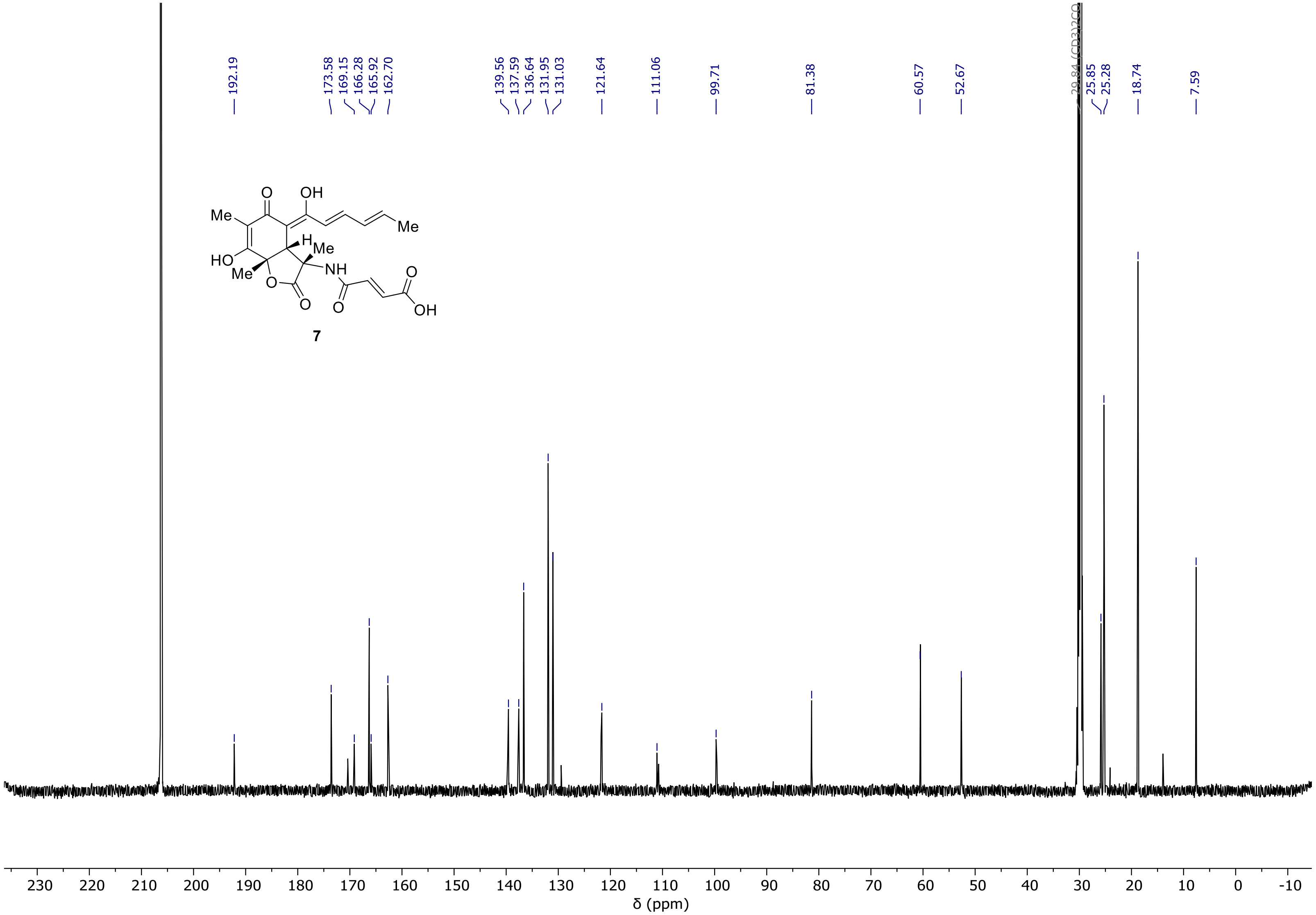

Fig. S55 ^{13}C-NMR spectrum of sorbicillactone A (**7**), measured in acetone-d_6 at 151 MHz.

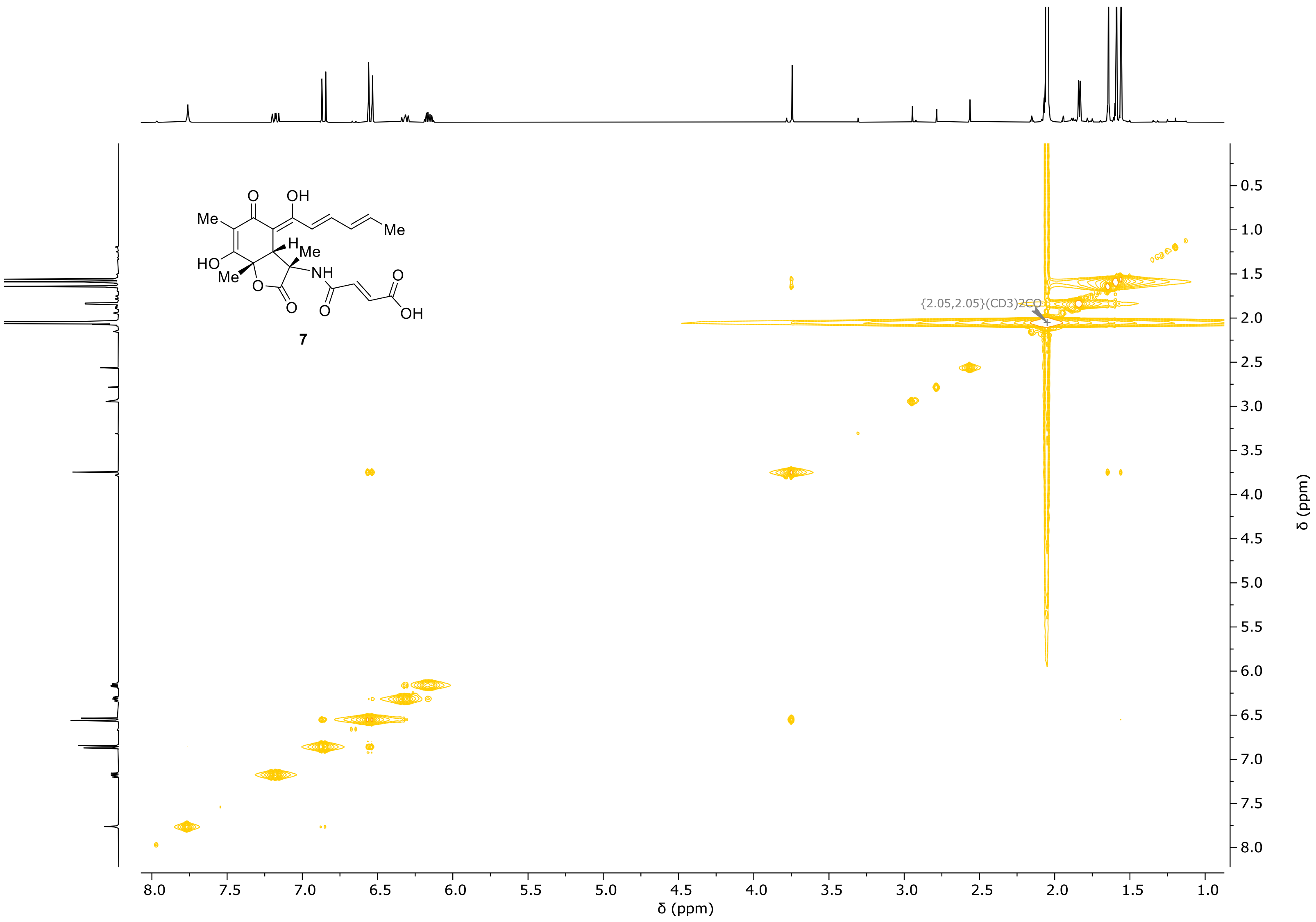

Fig. S56 NOESY spectrum of sorbicillactone A (**7**), measured in acetone-d_6.

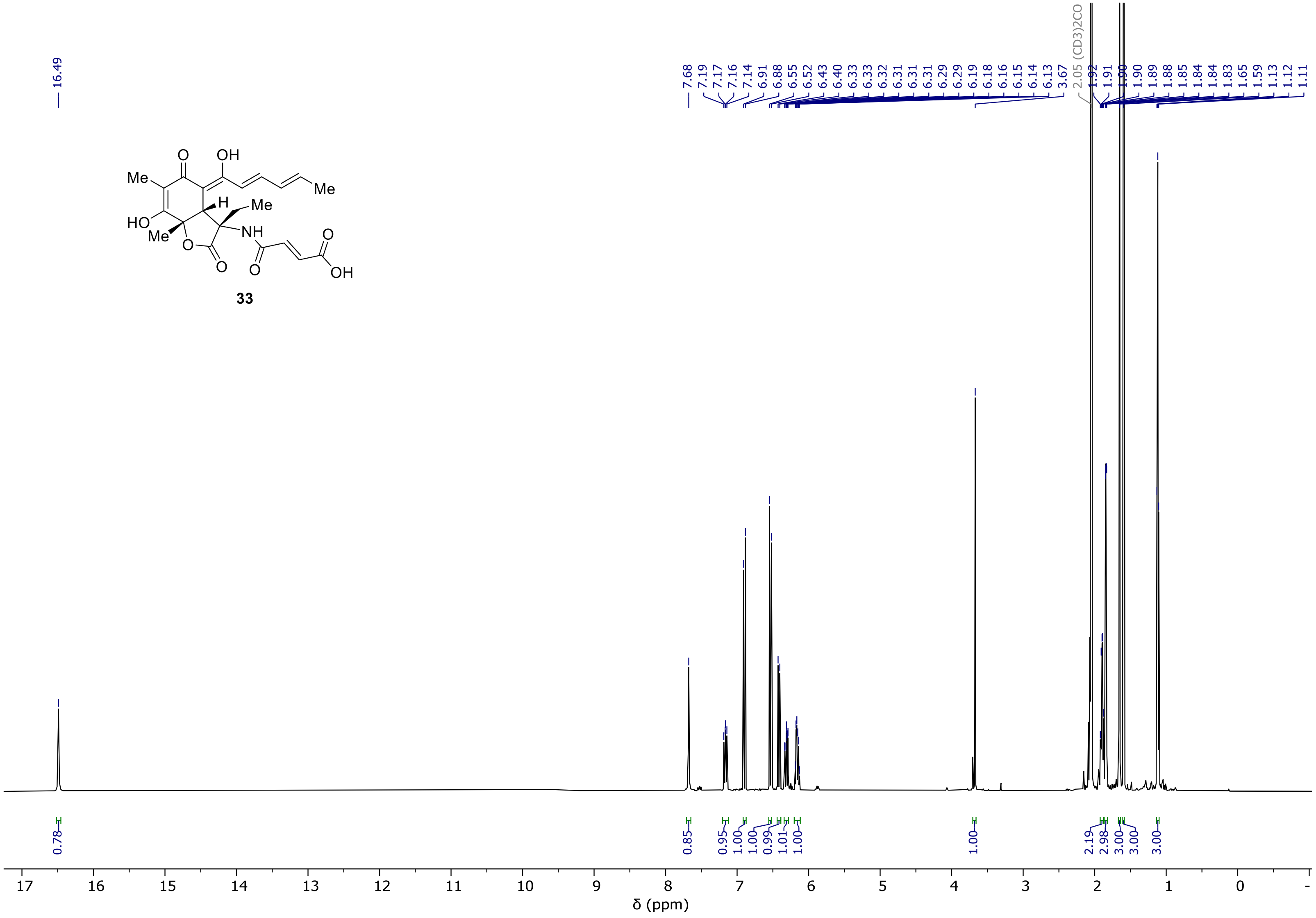

Fig. S57 ^{1}H-NMR spectrum of 9-ethyl sorbicillactone A (**33**), measured in acetone-d_6 at 600 MHz.

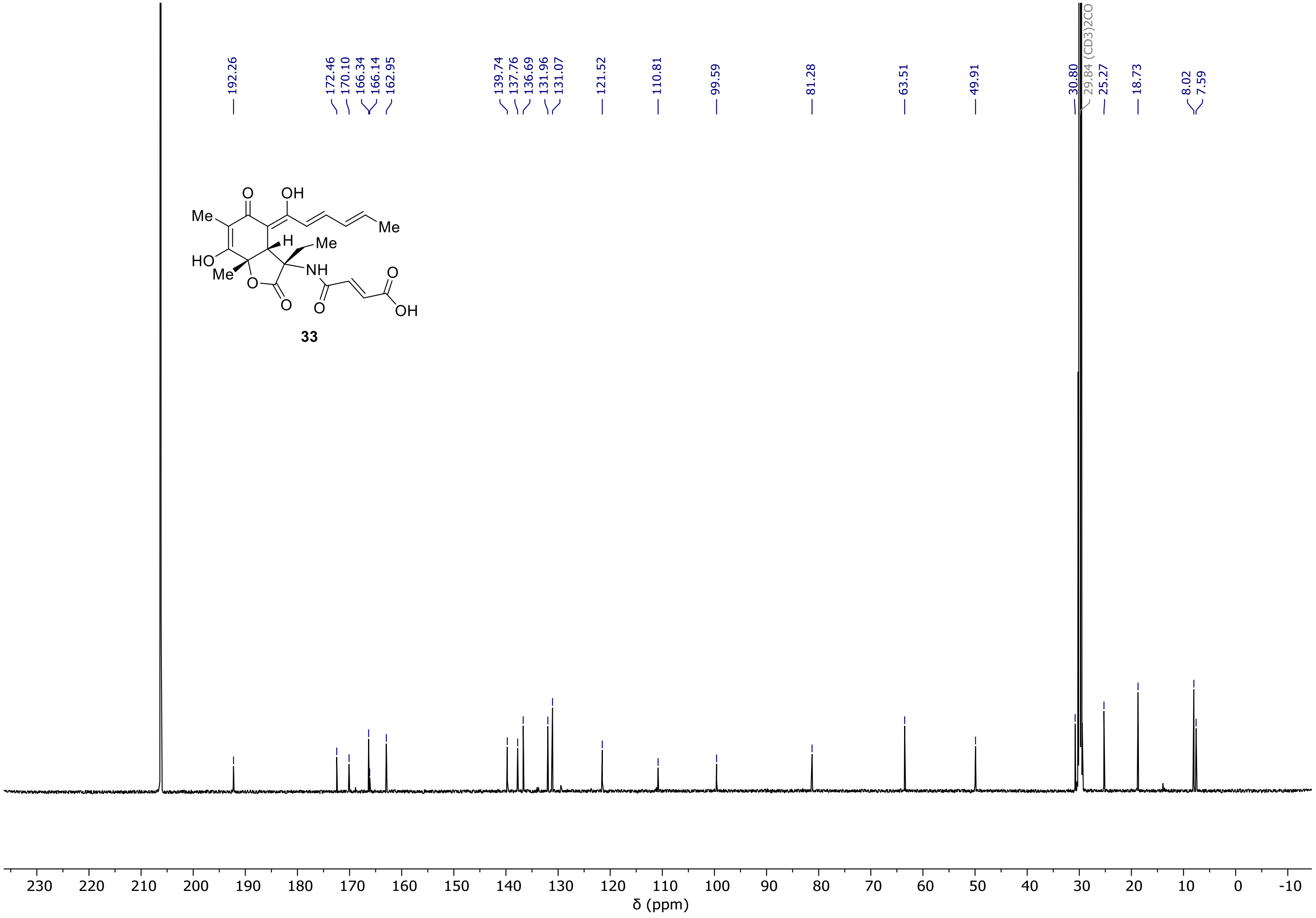

Fig. S58 ^{13}C-NMR spectrum of 9-ethyl sorbicillactone A (**33**), measured in acetone-d_6 at 151 MHz.

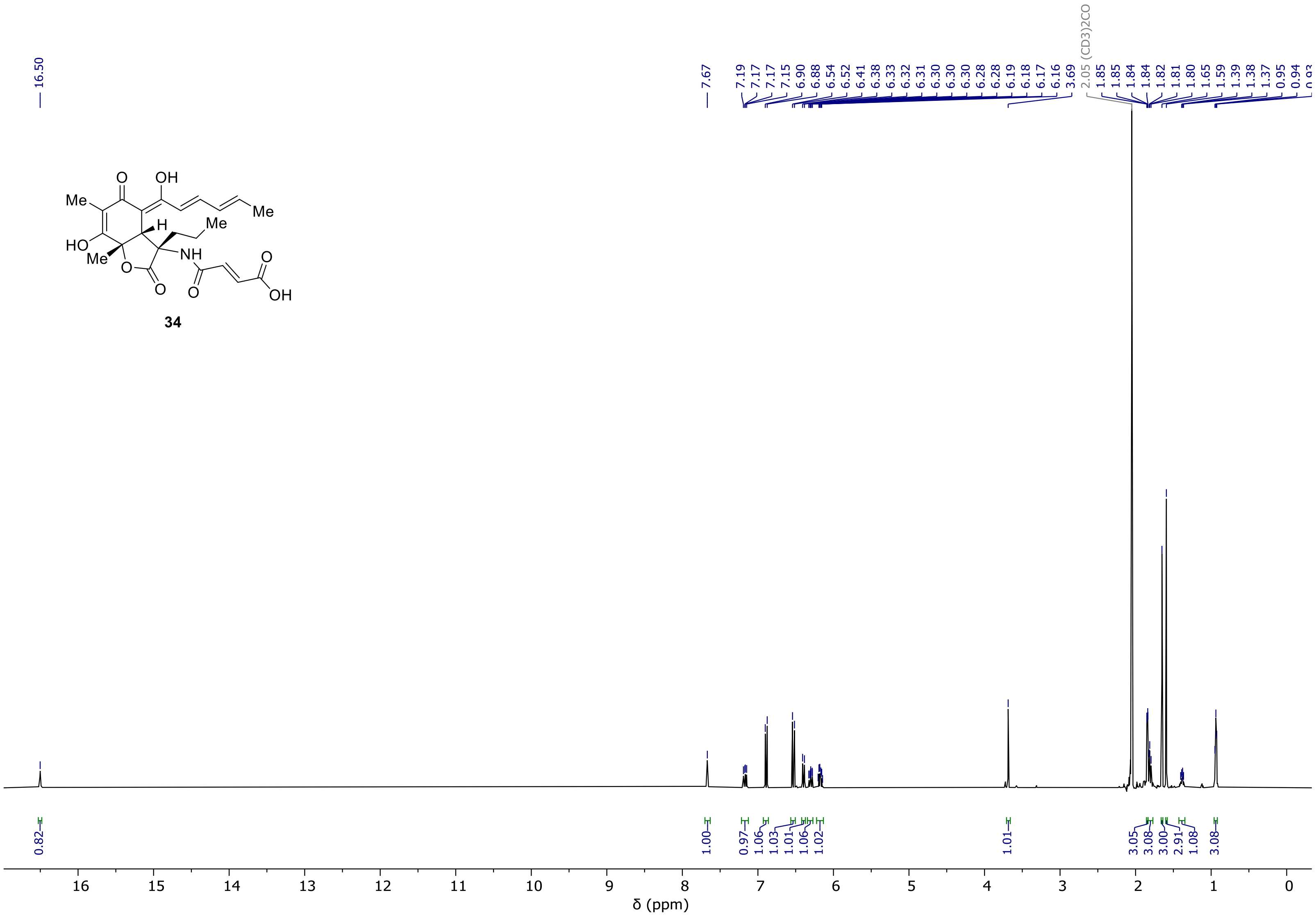

Fig. S59 ^{1}H-NMR spectrum of 9-propyl sorbicillactone A (**34**), measured in acetone-d_6 at 600 MHz.

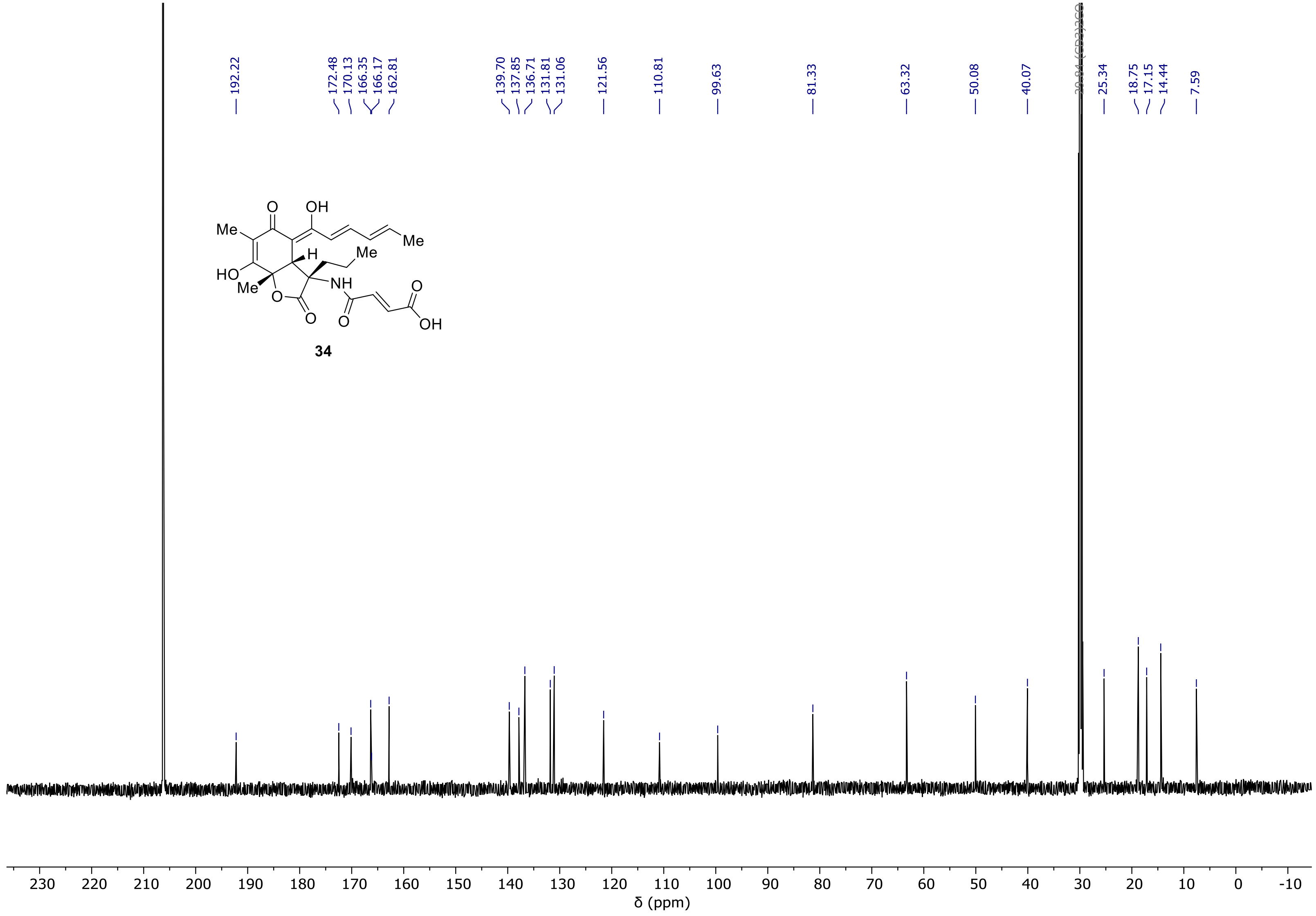

Fig. S60 ^{13}C-NMR spectrum of 9-propyl sorbicillactone A (**34**), measured in acetone-d_6 at 151 MHz.

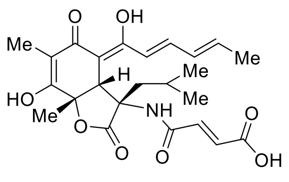

35

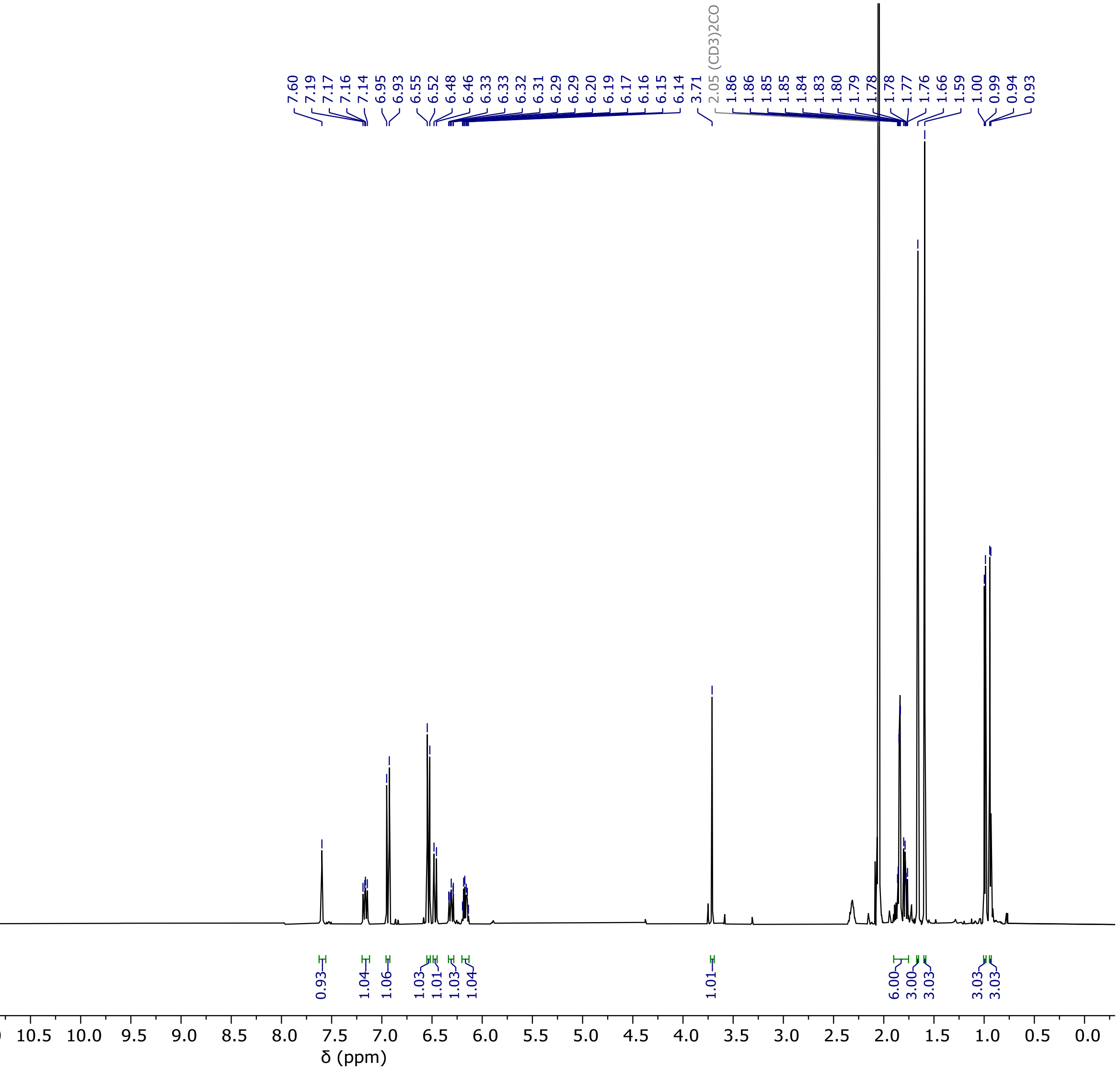

Fig. S61 ^{1}H-NMR spectrum of 9-isobutyl sorbicillactone A (**35**), measured in acetone-d_6 at 600 MHz.

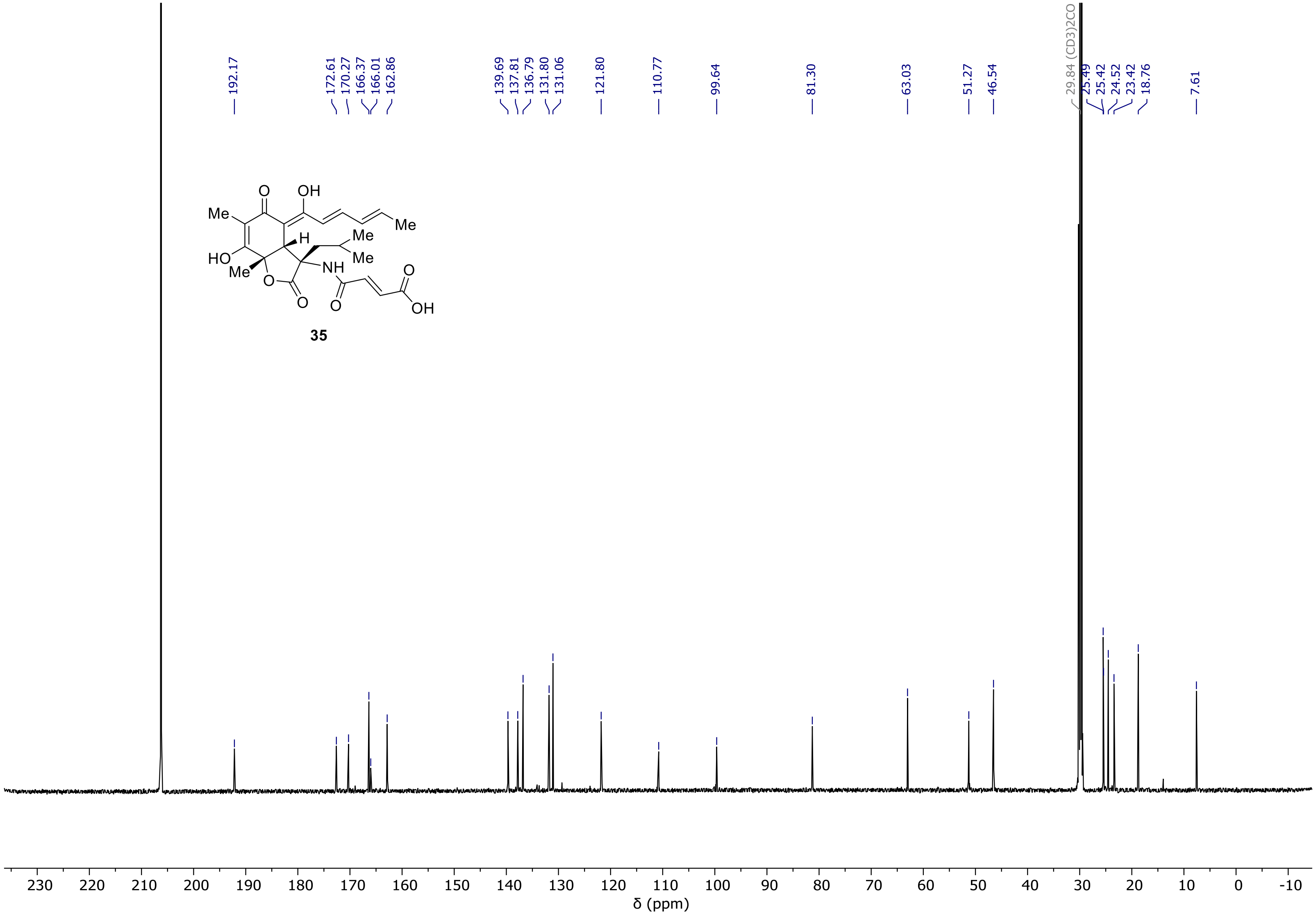

Fig. S63 ^{13}C-NMR spectrum of 9-isobutyl sorbicillactone A (**35**), measured in acetone-d_6 at 151 MHz.

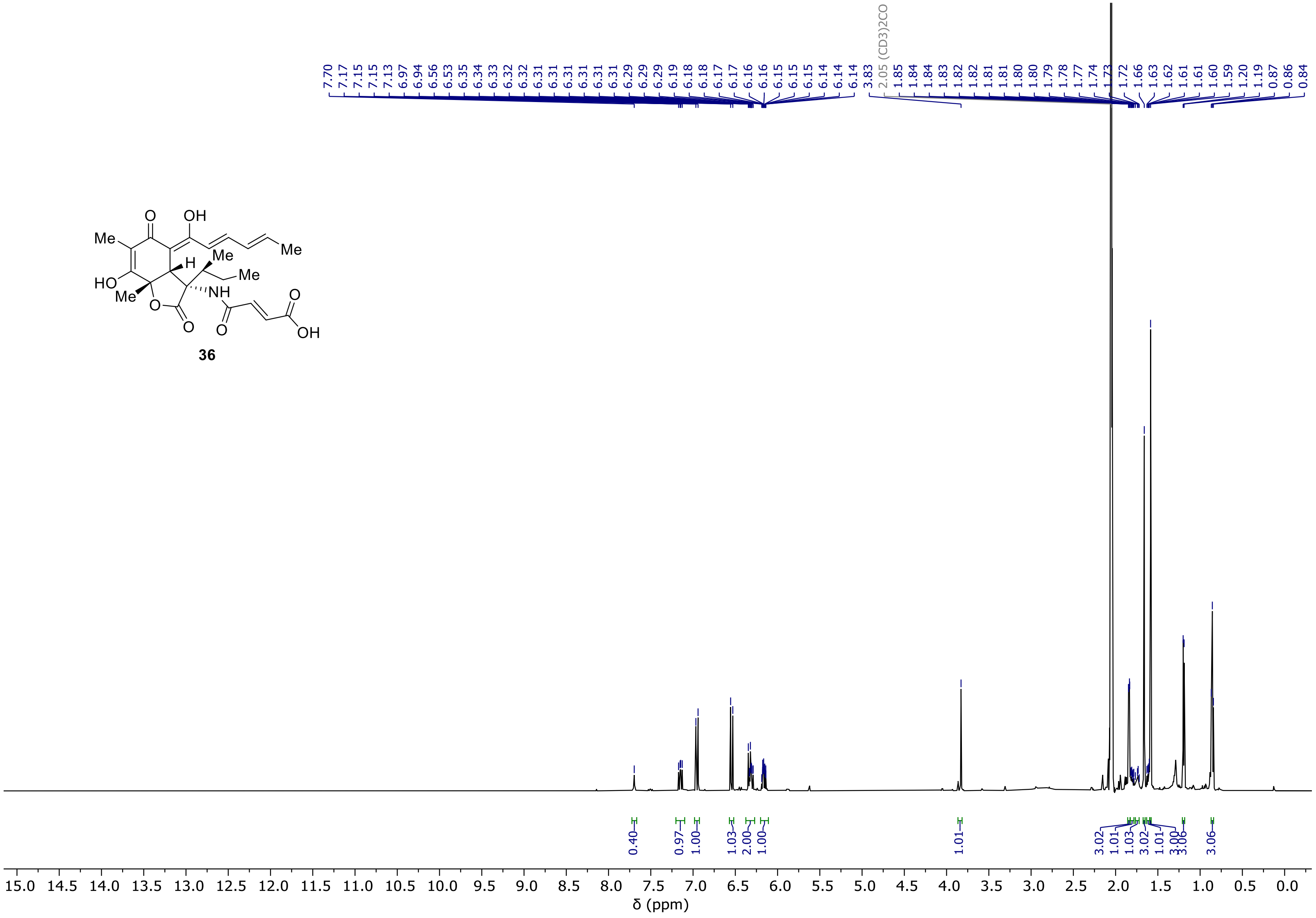

36

Fig. S62 ^{1}H-NMR spectrum of 9-*sec*-butyl sorbicillactone A (**36**), measured in acetone-d_6 at 600 MHz.

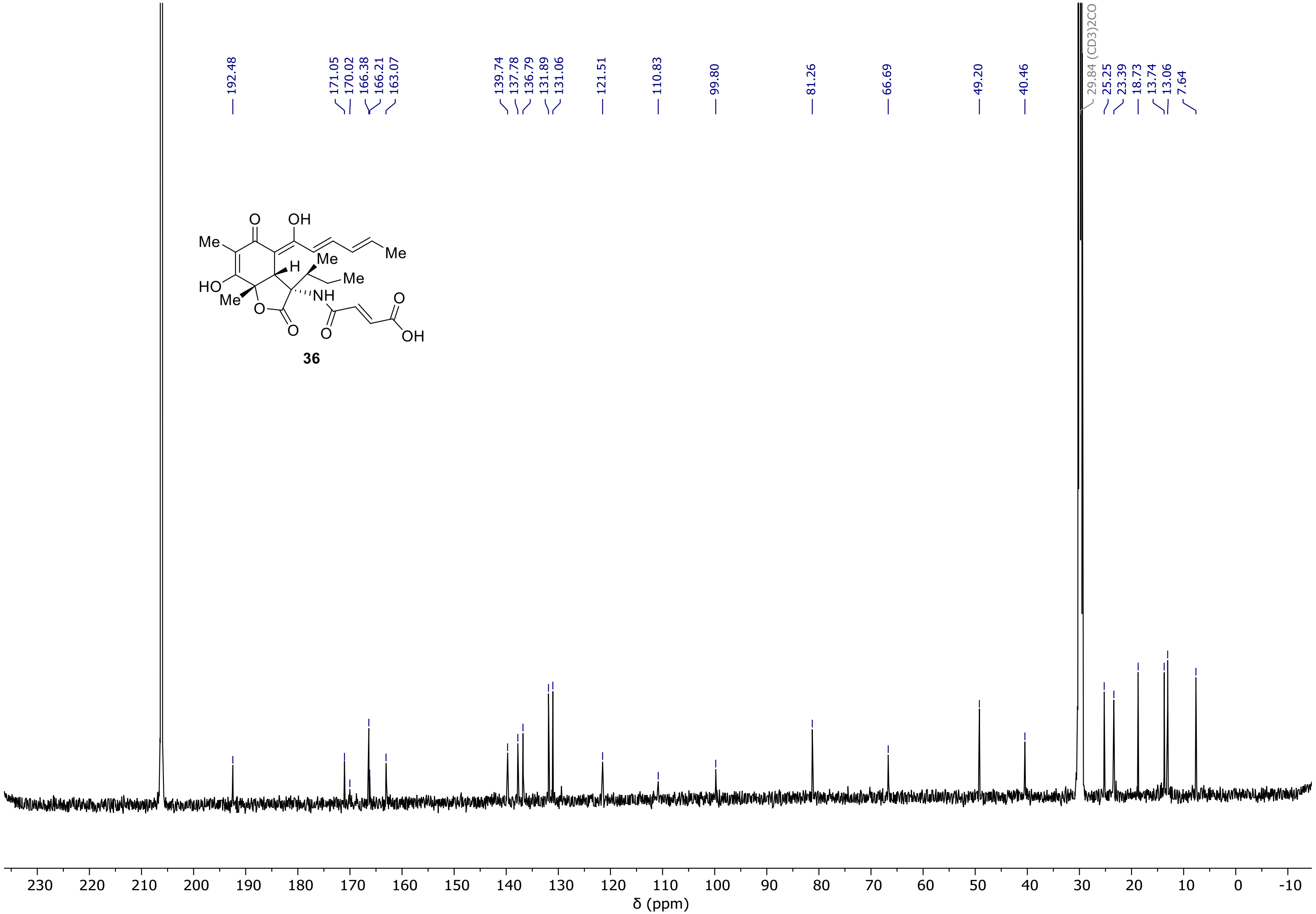

Fig. S64 ^{13}C-NMR spectrum of 9-*sec*-butyl sorbicillactone A (**36**), measured in acetone-d_6 at 151 MHz.
